# Plasmon-Determined Selectivity in Photocatalytic Transformations on Gold and Gold–Palladium Nanostructures

**DOI:** 10.1021/acsphotonics.3c00893

**Published:** 2023-08-30

**Authors:** Zhandong Li, Sadaf Ehtesabi, Siddhi Gojare, Martin Richter, Stephan Kupfer, Stefanie Gräfe, Dmitry Kurouski

**Affiliations:** † Department of Biochemistry and Biophysics, Texas A&M University, College Station, Texas 77843, United States; ‡ Institute of Physical Chemistry and Abbe Center of Photonics, Friedrich Schiller University Jena, Helmholtzweg 4, 07743 Jena, Germany; § Department of Biomedical Engineering, Texas A&M University, College Station, Texas 77843, United States

**Keywords:** gold nanostructures, gold@palladium nanoplates, plasmonics, photocatalysis, TERS imaging, DFT

## Abstract

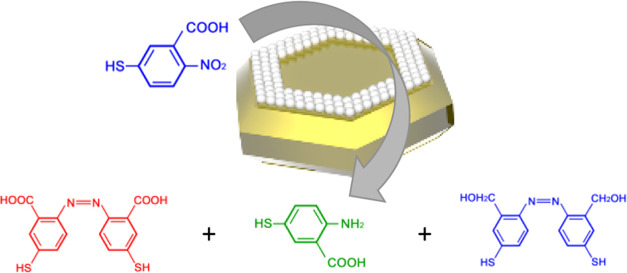

Noble metal nanostructures absorb light producing coherent oscillations of the metal’s electrons, so-called localized surface plasmon resonances (LSPRs). LSPRs can decay generating hot carriers, highly energetic species that trigger chemical transformations in the molecules located on the metal surfaces. The number of chemical reactions can be expanded by coupling noble and catalytically active metals. However, it remains unclear whether such mono- and bimetallic nanostructures possess any sensitivity toward one or another chemical reaction if both of them can take place in one molecular analyte. In this study, we utilize tip-enhanced Raman spectroscopy (TERS), an emerging analytical technique that has single-molecule sensitivity and sub-nanometer spatial resolution, to investigate plasmon-driven reactivity of 2-nitro-5-thiolobenzoic acid (2-N-5TBA) on gold and gold@palladium nanoplates (AuNPs and Au@PdNPs). This molecular analyte possesses both nitro and carboxyl groups, which can be reduced or removed by hot carriers. We found that on AuNPs, 2-N-5TBA dimerized forming 4,4′-dimethylazobenzene (DMAB), the bicarbonyl derivative of DMAB, as well as 4-nitrobenzenethiol (4-NBT). Our accompanying theoretical investigation based on density functional theory (DFT) and time-dependent density functional theory (TDDFT) confirmed these findings. The theoretical analysis shows that 2-N-5TBA first dimerized forming the bicarbonyl derivative of DMAB, which then decarboxylated forming DMAB. Finally, DMAB can be further reduced leading to 4-NBT. This reaction mechanism is supported by TERS-determined yields on these three molecules on AuNPs. We also found that on Au@PdNPs, 2-N-5TBA first formed the bicarbonyl derivative of DMAB, which is then reduced to both bihydroxyl-DMAB and 4-amino-3-mercaptobenzoic acid. The yield of these reaction products on Au@PdNPs strictly follows the free-energy potential of these molecules on the metallic surfaces.

## Introduction

Noble metal nanostructures can absorb light generating localized surface plasmon resonances (LSPRs).^1−3^ LSPRs are coherent oscillations of conductive electrons that can either dissipate producing heat or decay into hot carriers via direct inter band, phonon-assisted intra band, and geometry-assisted transitions.^4−7^ Hot carriers include hot electrons and holes, highly reactive species that^1,8,9^ can be directly or indirectly injected into molecular orbitals of analytes located in the close proximity to the metal surfaces.^10−12^ This triggers chemical reactions in such molecules, a phenomenon known as plasmon-driven catalysis.^13−15^ Hot electrons and holes also have unequal rates of dissipation, which results in the accumulation of hot carriers with lower transfer rates between a metal surface and the surrounding medium.^8^ This asymmetry in dissipation rates creates an electrostatic potential, which, in turn, can also trigger chemical reactions.^16,17^

Plasmon-driven chemistry on monometallic nanostructures is an active area of research for a large number of chemists and physicists.^16−22^ It has been shown that 4-nitrobenzenethiol (4-NBT) can be reduced to 4,4′-dimethylazobenzene (DMAB) on both gold and silver nanostructures.^23,24^ Furthermore, under acidic conditions, DMAB could be split into two molecules of 4-aminothiophenol (4-ATP), whereas under alkaline conditions, 4-NBT instead of 4-ATP could be expected. The Tian group demonstrated experimental evidence of plasmon-driven oxidation of 4-ATP to DMAB on noble metal nanostructures,^25^ whereas the Yoon group found that 4-mercaptobenzoic acid (4-MBA) could be decarboxylated on such nanostructures producing benzenethiol.^26^ Our research group as well as the group of El-Khoury discovered that 4-NBT could be ionized on gold and silver nanostructures producing 4-nitrobenzenethiolate.^27−30^ Recently, the Jain group demonstrated that CO_2_ could be reduced on gold nanostructures into C_1_–C_3_ hydrocarbons.^31,32^ Furthermore, a direct relationship between the intensity of the incident laser light and the product selectivity was observed. Specifically, an increase in the light intensity enhanced the selectivity of the C–C formation reaction in the order of C1 > C2 > C3.^31,32^

The number of plasmon-driven reactions can be expanded by coupling noble and catalytically active metals.^33,34^ In this case, localized surface plasmons generated by plasmonic metals are passed onto their catalytic counterparts, such as platinum and palladium.^23,35−38^ The Halas group found that bimetallic nanostructures could be used for a light-driven Suzuki coupling reaction, as well as for dry reforming of methane with carbon dioxide, a reaction that yields syngas.^15^ It should be noted that the yield of such bimetallic nanostructures is directly determined by the interplay between plasmonic and catalytic metals at the nanoscale.^39−42^ This nanoscale architecture of bimetallic structures could be revealed using tip-enhanced Raman spectroscopy (TERS).^43,44^ In TERS, LSPRs are generated at the apex of the metallized scanning probe by its illumination with a laser.^45^ LSPRs enhance the Raman scattering from molecules located directly under the probe.^46,47^ Consequently, if the probe is rastered above the sample surface, a corresponding chemical map can be acquired.^22^ TERS has single-molecule sensitivity and sub-nanometer spatial resolution.^43,48^ These two critically important parameters make TERS suitable for site-specific elucidation of the rate and yield of plasmon-driven reactions. Using TERS, Jiang’s group was able to perform site-specific catalysis with angstrom spatial resolution^49^ while the groups of Zenobi and Ren revealed that TERS can be also used to probe photocatalytic transformations at the interface of plasmonic and catalytic metals, as well as to unravel molecular conformations on metallic surfaces.^50^ In this context, our group previously utilized TERS to investigate plasmon-driven reactivity and selectivity on gold-platinum nanoplates (Au@PtNPs).^23,35^ We found that Au@PtNPs couple could be used to oxidize 4-ATP directly to 4-NBT, whereas only a partial oxidation of the corresponding reactant to DMAB was observed on their monometallic analogues, gold nanoplates (AuNPs).^23,35^ Gold-palladium nanoplates Au@PdNPs were capable of reducing 4-NBT to both 4-ATP and DMAB, whereas only DMAB was found as a plasmon-driven reduction product on AuNPs.^35^ We also found that Au@PdNPs could be used to reduce 4-MBA to 4-mercapto-phenyl-methanol (4-MPM), whereas these molecular analytes simply decarboxylates on AuNPs at the same experimental conditions.^51^

## Results and Discussion

**Scheme 1:**
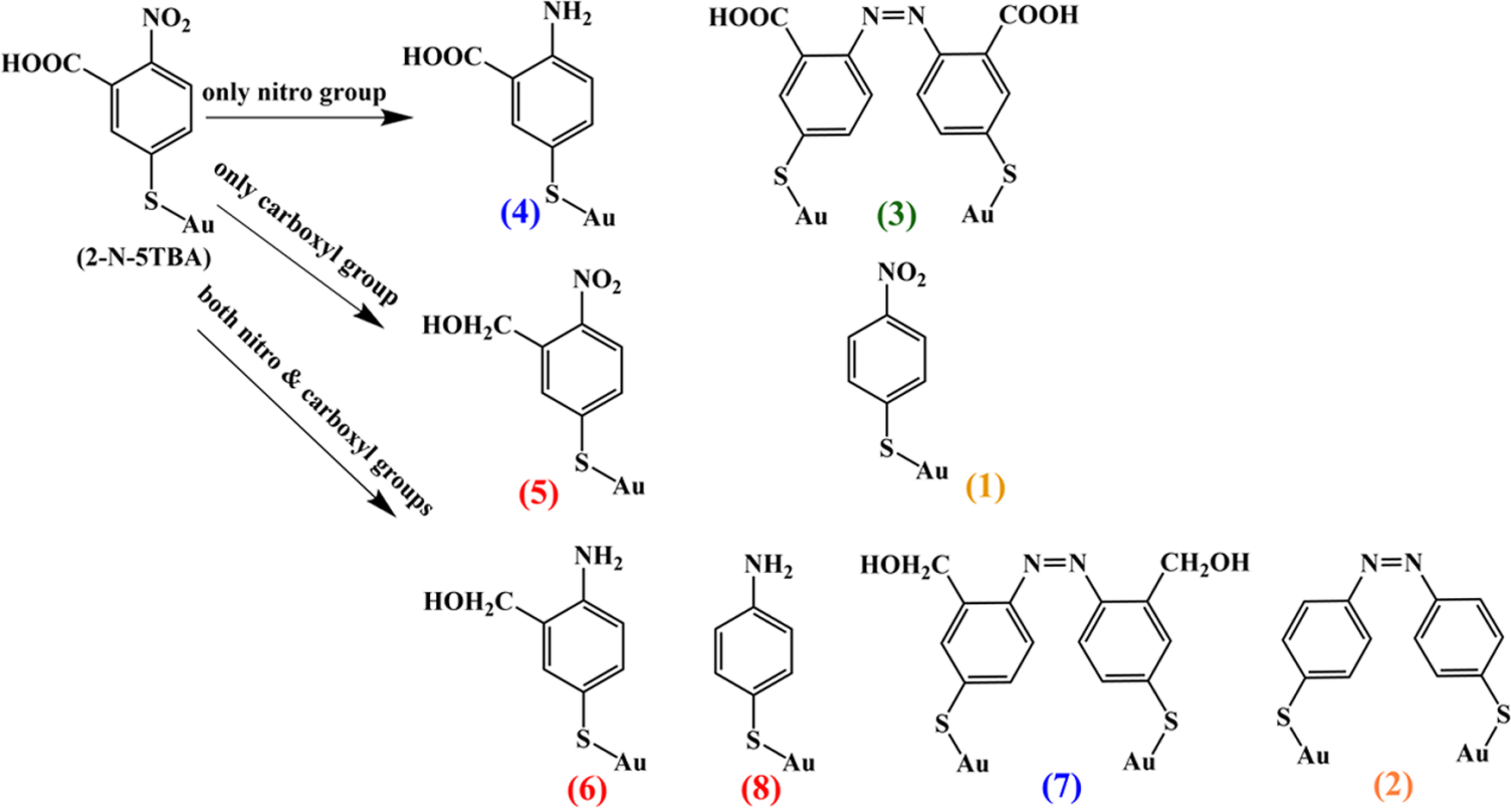
Possible Reaction Products That May Be Observed upon Plasmon-Driven Reduction of 2-N-5TBA The molecules labeled in blue and orange were observed in the presence of Au@PdNPs and AuNPs, respectively. The green-labeled molecule was produced on both Au@PdNPs and AuNPs. The red-labeled molecules were not observed in the experiment.

The question arises whether plasmons could selectively trigger one *vs* another chemical transformation if both can take place in the same molecular analyte. To address this question, we investigated the reactivity of 2-nitro-5-thiolobenzoic acid (2-N-5TBA) on AuNPs and Au@PdNPs. This molecule can undergo a plasmon-driven reduction of its nitro group and yields molecules with amino and azo groups, respectively (see Scheme 1). In parallel, one can also expect to observe plasmon-driven transformations involving exclusively the carboxyl group that will yield 4-NBT (product 1) or its derivative (product 5). Finally, simultaneous transformations involving both the amino and the carboxyl group could be expected for this molecular analyte. In this case, four reaction products are to be expected (i.e., products 2 and 6–8), shown in Scheme 1.

**Figure 1:**
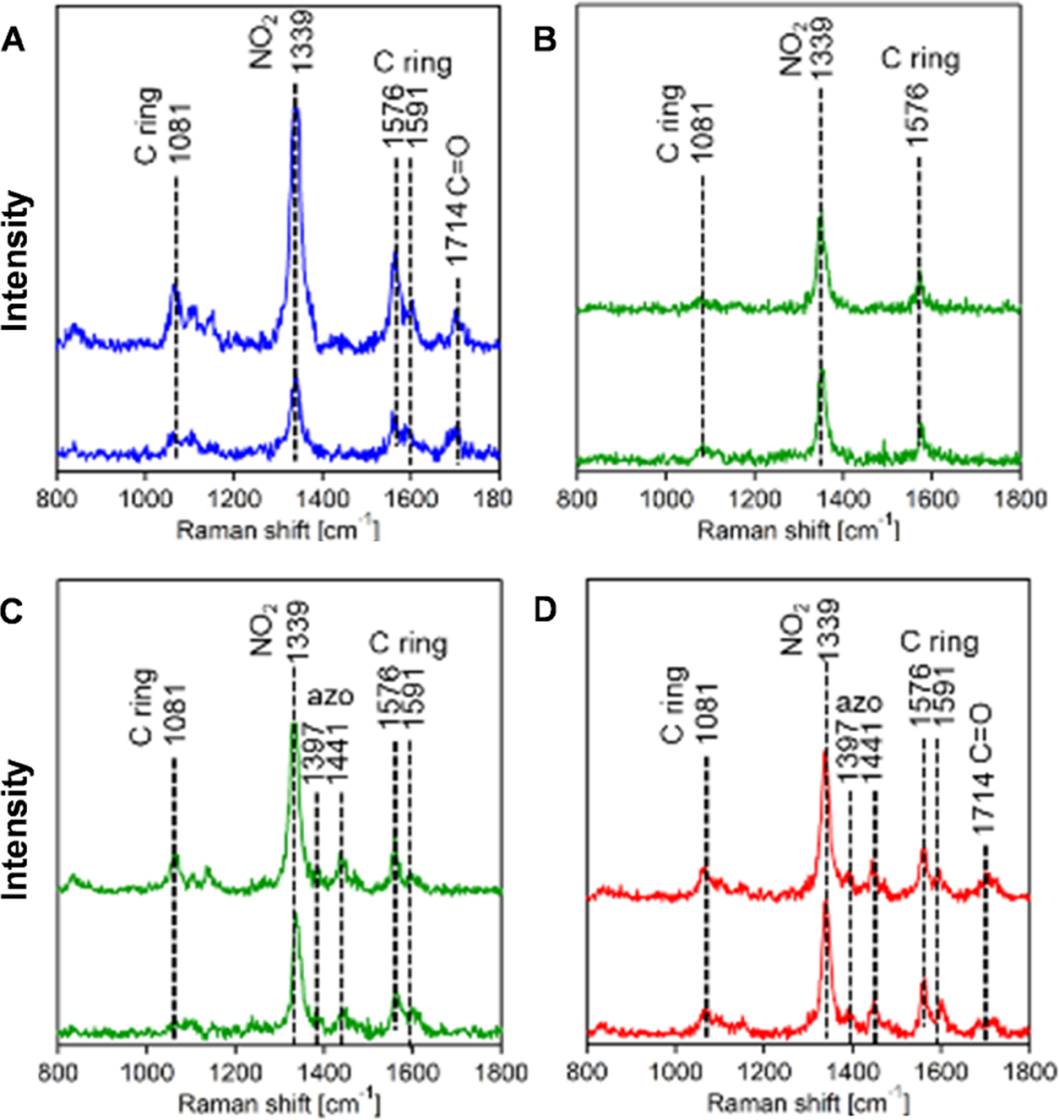
TERS spectra of (A) 2-N-5TBA, (B) product (1), (C) products (2 and 7), and (D) product (3).

TERS is uniquely suitable to disentangle such chemical transformations because the molecular analytes discussed in Scheme 1 exhibit drastically different vibrational fingerprints (Table 1). This will allow for unambiguous identification of these molecules based on the TERS spectra acquired from the surface of AuNPs and Au@PdNPs decorated with a monolayer of 2-N-5TBA. Specifically, the TERS spectrum of 2-N-5TBA features five distinct vibrational bands at 1081 cm^–1^ (ring), 1339 cm^–1^ (NO_2_), 1576–1591 cm^–1^ (ring), and 1714 cm^–1^ (COOH) (Figure 1 and Table 1). Product (1) (4-NBT) has only three Raman-active vibrational bands at 1083, 1339, and 1576 cm^–1^, whereas the product (2) (DMAB) has a set of vibrational bands at 1081, 1339, 1397, 1441, and 1576 cm^–1^ (Figure 1). The carboxylated-DMAB (3) can be identified by the presence of a COOH band at 1714 cm^–1^ in addition to the vibrational signatures of DMAB; see Scheme 1 as well as Figure 1 and Table 1. Product (4) could be identified by its amino and carboxyl vibrations. We would expect to detect NO_2_ vibration in product (5), whereas product (7) can be identified by the characteristic azo (1397 and 1441 cm^–1^) vibrations. Finally, product (8) would exhibit TERS spectra with only amino group vibration. It should be noted that TERS is not capable of disentangling between products (2) and (7) because the vibrational spectra of both will be identical. However, our previously reported results demonstrate that carboxyl groups could be reduced only in the presence of Pd.^51^ On AuNPs, exclusively decarboxylation is taking place. Thus, on AuNPs, we could expect only product (2), whereas on Au@PdNPs, product (7) was formed from 2-N-5TBA. One can also expect that products (6) and (8) are indistinguishable based on their TERS spectra. However, our results showed that none of these products were observed upon TERS imaging of the plasmon-driven reactions on both AuNPs and Au@PdNPs.

**Table 1:** Vibrational Bands Expected for 2-N-5TBA and Molecular Products (1-8) (Scheme )1

compound	NH_2_ (1485,1586 cm^–1^)	NO_2_ (1339 cm^–1^)	COOH (1714 cm^–1^)	N=N (1397, 1441 cm^–1^)
(2-N-5TBA)	N	Y	Y	N
(1)	N	Y	N	N
(2)	N	N	N	Y
(3)	N	N	Y	Y
(4)	Y	N	Y	N
(5)	N	Y	N	N
(6)	Y	N	N	N
(7)	N	N	N	Y
(8)	Y	N	N	N

TERS analysis of plasmon-driven reduction of 2-N-5TBA on both AuNPs revealed the formation of only three molecular analytes. Specifically, we observed the formation of products (1), (2), and (3).

Experimental results reported recently by our group demonstrated that TERS could be used for the quantitative measurements of the yield of plasmon-driven reactions.^52−54^ Furthermore, the yield can be directly altered by the light intensity.^52^ Expanding upon this, we also aim to determine the relationship between the light intensity and the yield of the above discussed molecular analytes on AuNPs (Scheme 1).

**Figure 2:**
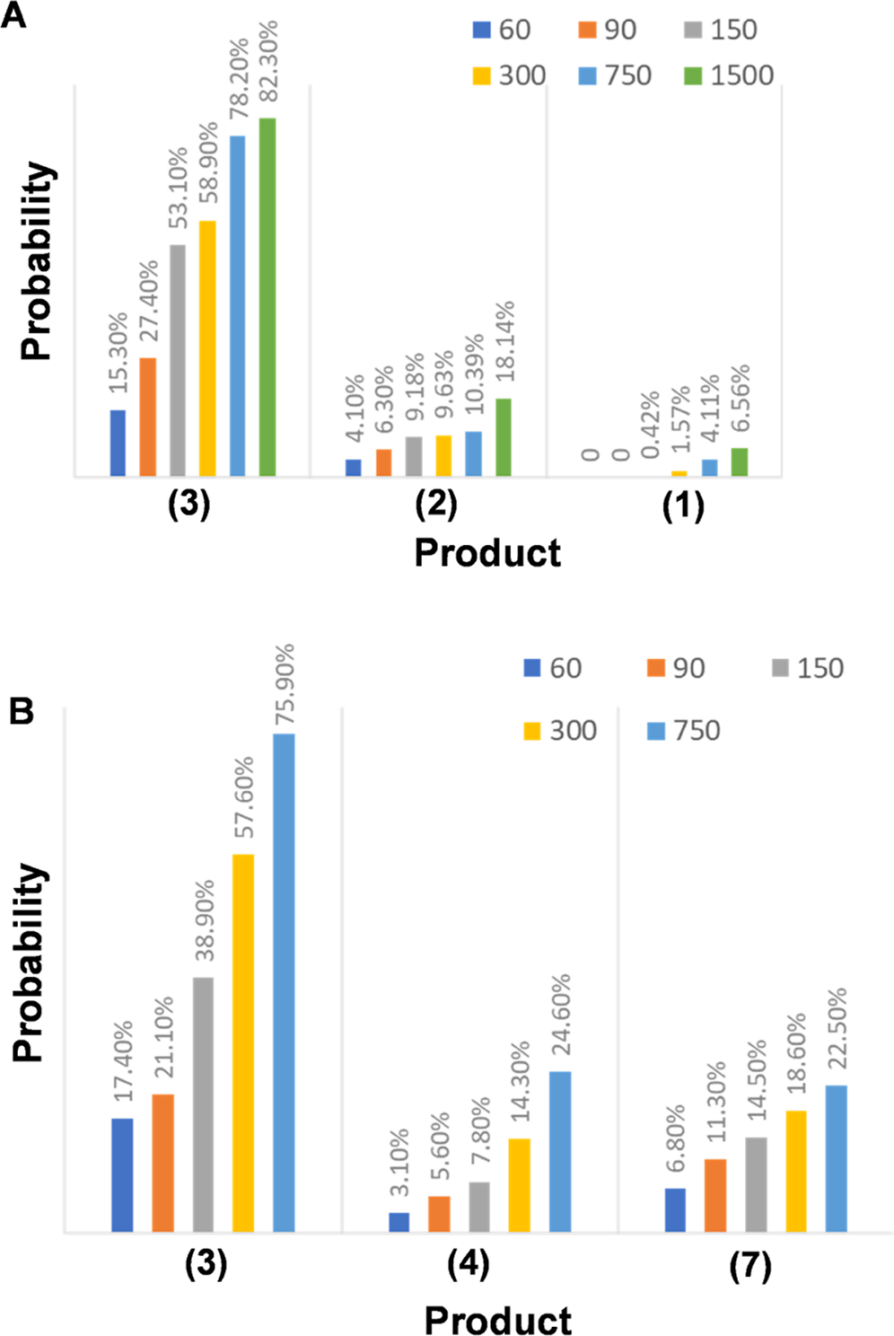
Histograms of the yield of the reduction products of 2-N-5TBA on (a) AuNPs and (b) Au@PdNPs at different light intensities (60–1500 μW of 633 nm laser light).

**Figure 3:**
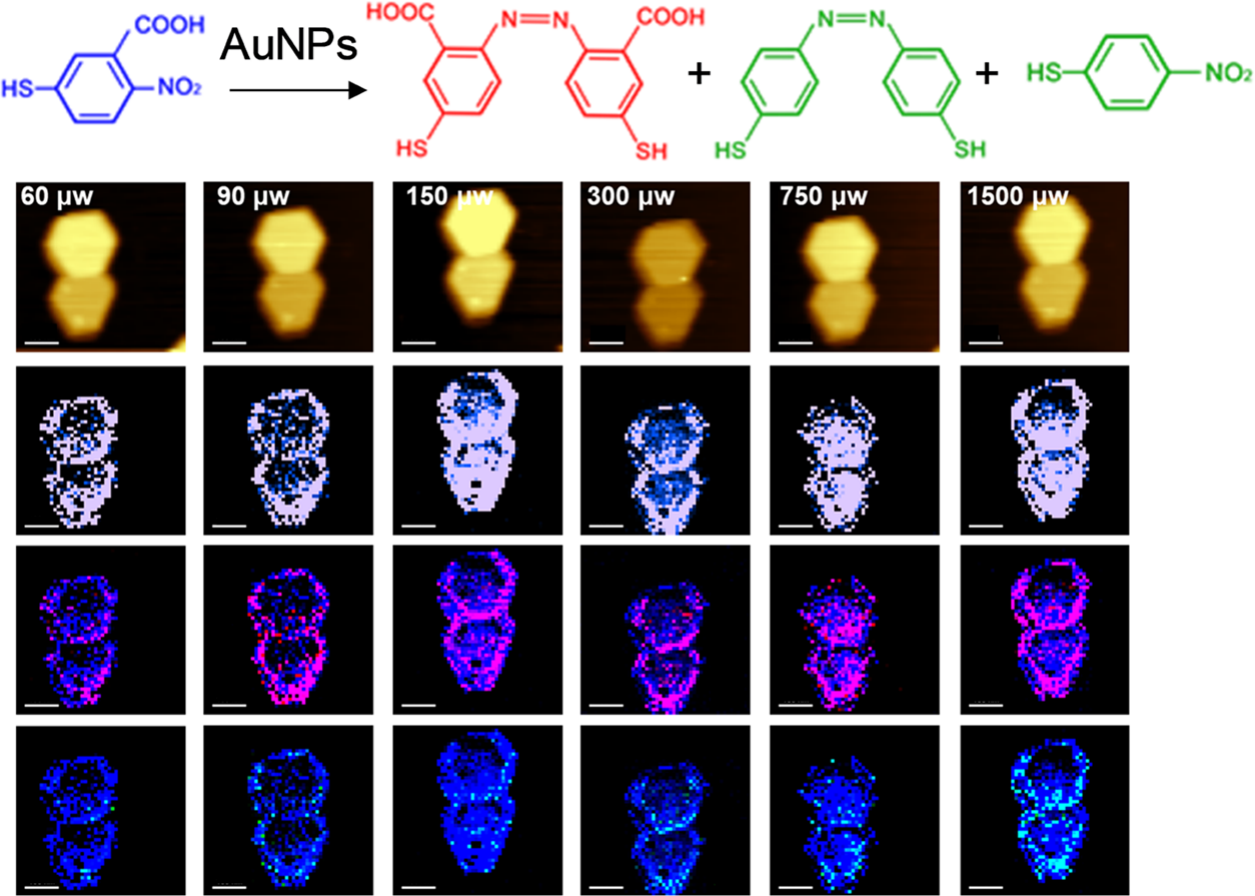
TERS imaging of 2-N-5TBA reduction on AuNPs at different light intensities (60–1500 μW of 633 nm laser light). In situ AFM images (top row) of AuNPs and corresponding TERS images of 2-N-5TBA (1339 cm^–1^) shown in blue (middle row), as well as the overlapped TERS images of 4-NBT (blue) and DMAB (1397 and 1441 cm^–1^) (red; bottom row). Scale bars are 100 nm.

We found that reaction products (1–3) increased their yields with an increase in light intensity. Specifically, we observed an increase in the yield of product (1) from 0% at 60–90 μW to 0.42% at 150 μW and 1.57% at 300 μW, respectively (Figures 2 and 3. Even higher yields of product (1) were observed at 633 nm (4.1%) and 1500 μW (6.6%). In a similar fashion to the observations made for product (1), we observed a gradual increase in the yield of product (2) from 4.1% (60 μW) to 18.1% (1500 μW). The same conclusion could be drawn from the product (3). We determined that the yield of this product increased from 15.3% (60 μW) to 82.3% (1500 μW); see Figures 2 and 3. Based on these results, a stronger preference for the formation of product (3) from 2-N-5TBA in comparison to products (2) and (3) is apparent at all applied light intensities. Furthermore, the formation of product (1) seems to be highly unfavorable.

We utilized theoretical modeling in order to rationalize the formation of the various products and their respective yields assisted by AuNPs. Thereby, the aims of our quantum chemical study were set on elucidating the thermodynamic properties of the respective intermediates and products as well as their individual TERS signatures by applying our recently introduced computational protocol.^55−58^ In particular, we focused on the reactivity of each moiety in 2-N-5TBA individually. In the following, we will begin with the nitro and proceed by the carboxyl group. In the present case, the nitro group can undergo reductive processes along two main pathways: either via a 6-electron reduction to the respective amine (NH_2_) or based on a four-electron reduction of two neighboring 2-N-5TBA (each) and subsequent dimerization to yield the azo-compound (N=N). In a similar fashion, two prominent reaction pathways are available for the carboxyl group, i.e., decarboxylation or reduction to alcohol (−CH_2_OH). From a mechanistic point of view, decarboxylation is not straightforward as this requires cleavage on a rather strong C–C bond. For this reason, efficient charge-transfer processes among the NP and the analyte must be available upon photoexcitation. Elucidating the product formation stemming from 2-N-5TBA under plasmonic conditions is further far from trivial as any combination of these four reaction pathways associated with the nitro and carboxyl groups may proceed simultaneously.

Initially, 2-N-5TBA was studied theoretically: The molecule was surface-immobilized on an Au slab and investigated using periodic density functional theory (DFT) to describe the structure and properties of the electronic ground state of the combined system. Subsequently, employing (nonperiodic) time-dependent DFT (TDDFT) calculations, the corresponding properties and signatures of the electronically excited states of the hybrid system were assessed (see the Supporting Information for details) to corroborate the experimental results. The lowest 600 electronic states of the plasmonic hybrid system were examined concerning their respective electronic characters, i.e., molecule-centered, metal-centered, metal-to-molecule, and molecule-to-metal charge transfer. It is necessary to investigate this large number of electronic states in order to address the electronic transitions that are accessible within the vicinity of the irradiating laser wavelength of 633 nm (1.96 eV). In the following, we restrain from the discussion of specific electronic excitations as the plasmonic hybrid system model features a plethora of highly mixed weakly absorbing transitions. This is in particular evident for the case of charge-transfer processes involving the Au slab and the surface-immobilized substrate. Further details regarding the computational setup and the electronic transitions can be found in Supporting Information and Tables S1–S7, respectively.

**Figure 4:**
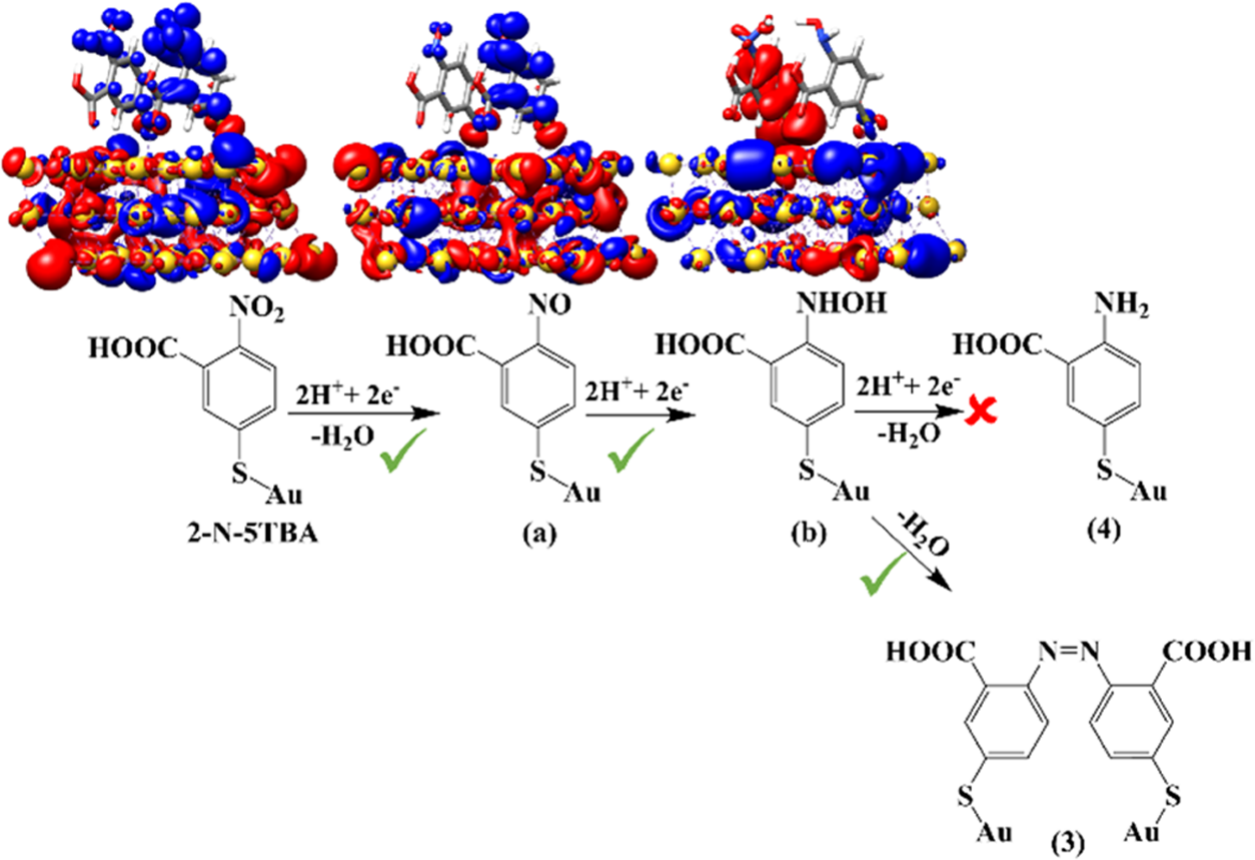
Charge-density differences (CDDs) of two sample molecules (left to right: 2-N-5TBA, a, and b) immobilized on an Au slab illustrate the nature of the low-lying dipole-allowed excitations in the vicinity of the 633 nm excitation. Charge transfer takes place from red to blue. Potential reaction pathways connecting 2-N-5TBA to products (4) and (3) are indicated. Pathways accessible by light-driven processes (at 633 nm, see CDDs) are highlighted by green ticks; energetically inaccessible pathways are marked by a red cross.

In general, pronounced charge-transfer processes between the gold slab and the surface-immobilized molecule are predicted, in particular of metal-to-molecule charge-transfer character. Such charge transfer leads ultimately to the reduction of the nitro and carboxyl groups – followed by dimerization and decarboxylation, respectively. The low (photo)reduction potential of the nitro group favors −NO_2_-related branching pathways concerning competing carboxyl-associated processes as confirmed by our quantum chemical calculations. Charge-density differences (CDDs) of optically accessible excited states of 2-N-5TBA@Au in the range of the laser excitation (Figure 4 and Table S1) reveal that the electronic transitions originating from the Au slab towards the nitro group contribute substantially more to these excited states than the excitations towards the carboxyl group. This computational finding demonstrates that initially the reduction of the nitro group occurs, which will be investigated in more detail in the following.

**Figure 5:**
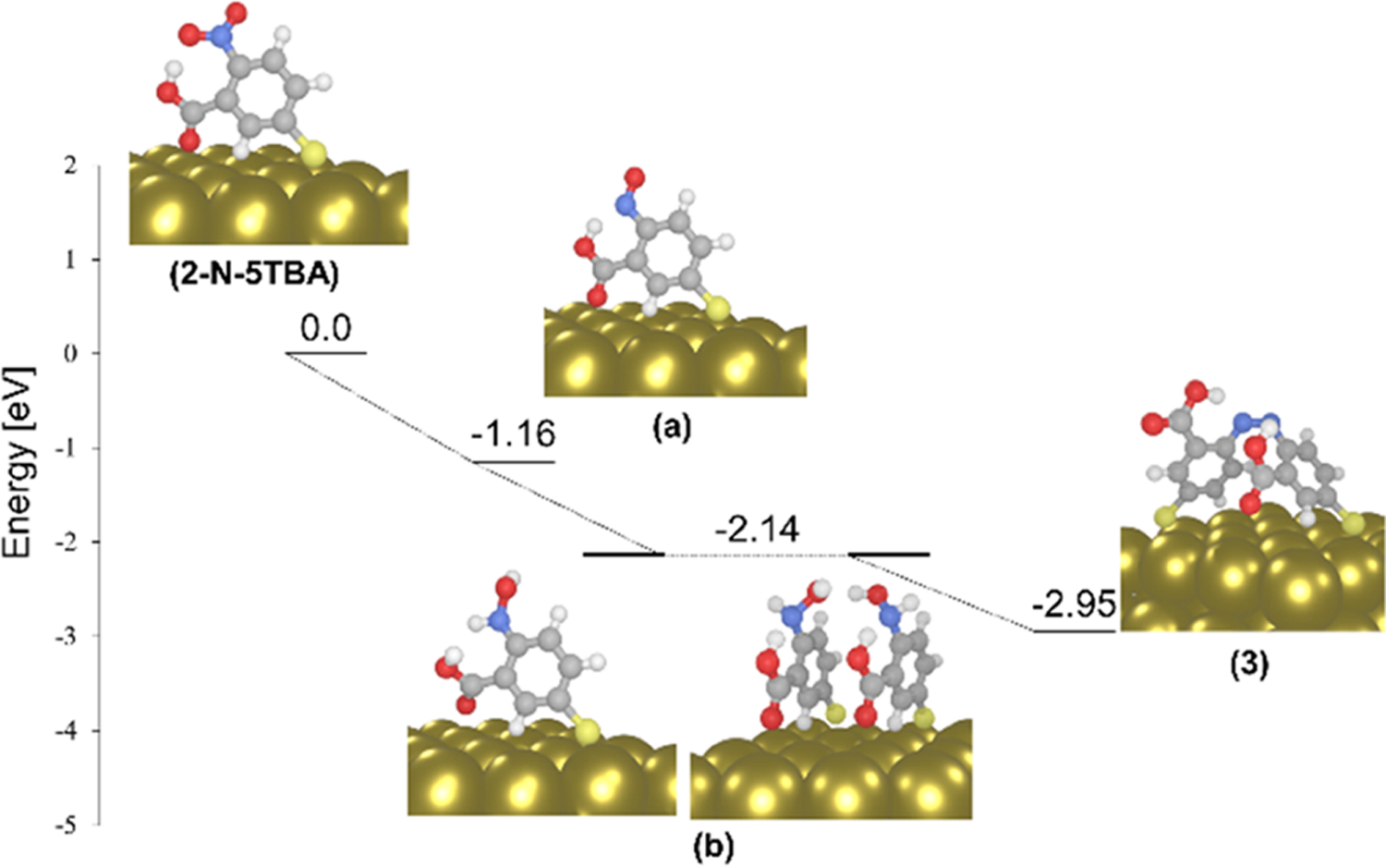
Ground-state reaction pathways for the formation of product (3) from 2-N-5TBA as predicted by periodic DFT calculations. The driving forces for each stepwise redox reaction. Structures of the surface-immobilized intermediates formed in the reaction pathways (a) and (b) are illustrated.

As shown in Scheme 1, the reduction of the nitro group in 2-N-5TBA could lead to products (3) and (4). As illustrated in Figure 4, the prominent metal-to-molecule charge-transfer transitions at ∼1.96 eV lead to the formation of intermediate (a), whereas the NO_2_ group is reduced to NO. Notably, this process requires a proton source, e.g., water under ambient conditions. This two-electron, two-proton transfer process yielding (a) is eventually followed by a consecutive light-driven metal-to-molecule charge transfer, which yields under ambient conditions (i.e., H^+^ source) intermediate (b). Based on our TDDFT simulations, a further reduction of (b) to the respective amine is impossible at the given excitation energy as further metal-to-molecule charge transfer transitions are energetically unfavorable and would be only observed at significantly higher excitation energies. This explains why the product (4) was not observed experimentally. However, two neighboring electron-rich intermediates (b) may dimerize to form product (3) by releasing two water molecules. In addition, the thermodynamical quantities (Figure 5) confirm that, under the experimental conditions, the formation of product (3) from the stepwise reduction of 2-N-5TBA is energetically feasible.

**Figure 6:**
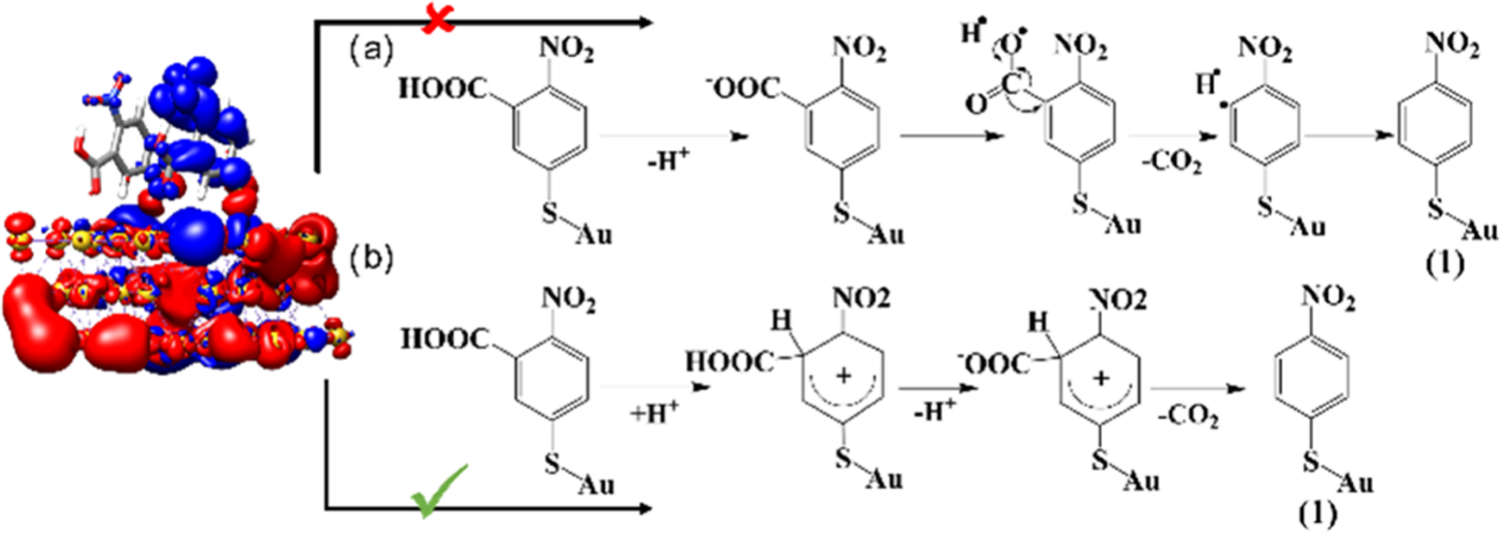
Charge-density difference for two 2-N-5TBA molecules on an Au slab in the range of 1.96 eV; charge transfer occurs from red to blue. Two general mechanisms of decarboxylation are shown: (a) via hole transfer and (b) via electron transfer. A green tick mark indicates the accessible process (@633 nm excitation).

**Figure 7:**
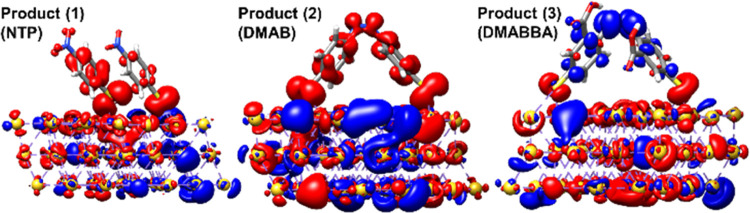
Charge-density differences (CDDs) of the low-lying dipole-allowed excitations at 633 nm of product (1), product (2), and product (3). Charge transfer takes place from red to blue.

As described in the literature, decarboxylation processes may proceed via two general mechanisms (Figure 6).^26,51,53^ One of these pathways takes place through hole transfer while the other occurs via an electron-transfer process. According to the excited state’s electronic structure of 2-N-5TBA in the range of laser radiation (CDD in Figure 6), an electron transfer-assisted mechanism is highly likely in the present case as electrons are transferred upon light interaction/excitation from the Au slab to the carboxyl group, specifically to the C–C bond, which facilitates bond breaking and consequently decarboxylation. In consequence, product (1) is obtained. The formation of product (1) via decarboxylation of 2-N-5TBA is also energetically feasible, forasmuch as the calculated driving force for this reaction is −0.13 eV. In addition to the decarboxylation of 2-N-5TBA, there is another possible pathway for the formation of product (1) which is the oxidation of product (2). The electronic character of the product (2) (Figure 7) confirms this hypothesis, as dipole-allowed molecule-to-metal charge-transfer transitions at ∼1.96 eV lead to the photooxidation of the product (2). Thus, the oxidation of product (2) can be performed entirely by light-driven processes. This is more likely to take place than the direct decarboxylation of 2-N-5TBA because, as long as the nitro group is present in the molecular structure, a significant amount of charge is transferred to the nitro group. This, in turn, will lead to the reduction and the formation of the azo moiety.

For now, our discussion was focused on the formation of products (3) and (1), which are mediated by either nitro or carboxyl group reactions. In the following, we will investigate the formation of product (2) via two pathways involving both carboxyl and nitro groups. The first pathway proceeds by reduction of the product (1) and dimerization to form product (2) while the second channel involves the decarboxylation of product (3). Under the given reaction condition, the electronic character of (1) (Figure 7) undoubtedly hampers the reductive pathway since an electron can only be transferred from the molecule to the Au slab – leading to the oxidation of NTP. As a result, there is no possibility of reducing product (1) at an excitation energy of 1.96 eV. As shown in Figure 7, the metal-to-molecule charge-transfer character of the product (3) allows the second mechanism to be realized in the same way as discussed previously regarding the decarboxylation of 2-N-5TBA. The favorable driving force for product (2) formation (−0.14 eV) via the decarboxylation of (3) is in line with the argumentation based on the key excited state processes. However, the electron transfer to the carboxyl group contributes only modestly to the optically accessible excited states in the range of 1.96 eV (see Table 4 in the supporting information). This finding demonstrates that such decarboxylation requires a high intensity of laser radiation.

Based on our theoretical investigations, we can conclude that under 633-nm excitation and the presence of AuNPs, 2-N-5TBA converts to product (3), then increased laser power leads to the decarboxylation of product (3), which results in the formation of product (2). Subsequently, and as a result of its electronic (excited state) structure, product (2) can be oxidized and converted to product (1).

**Figure 8:**
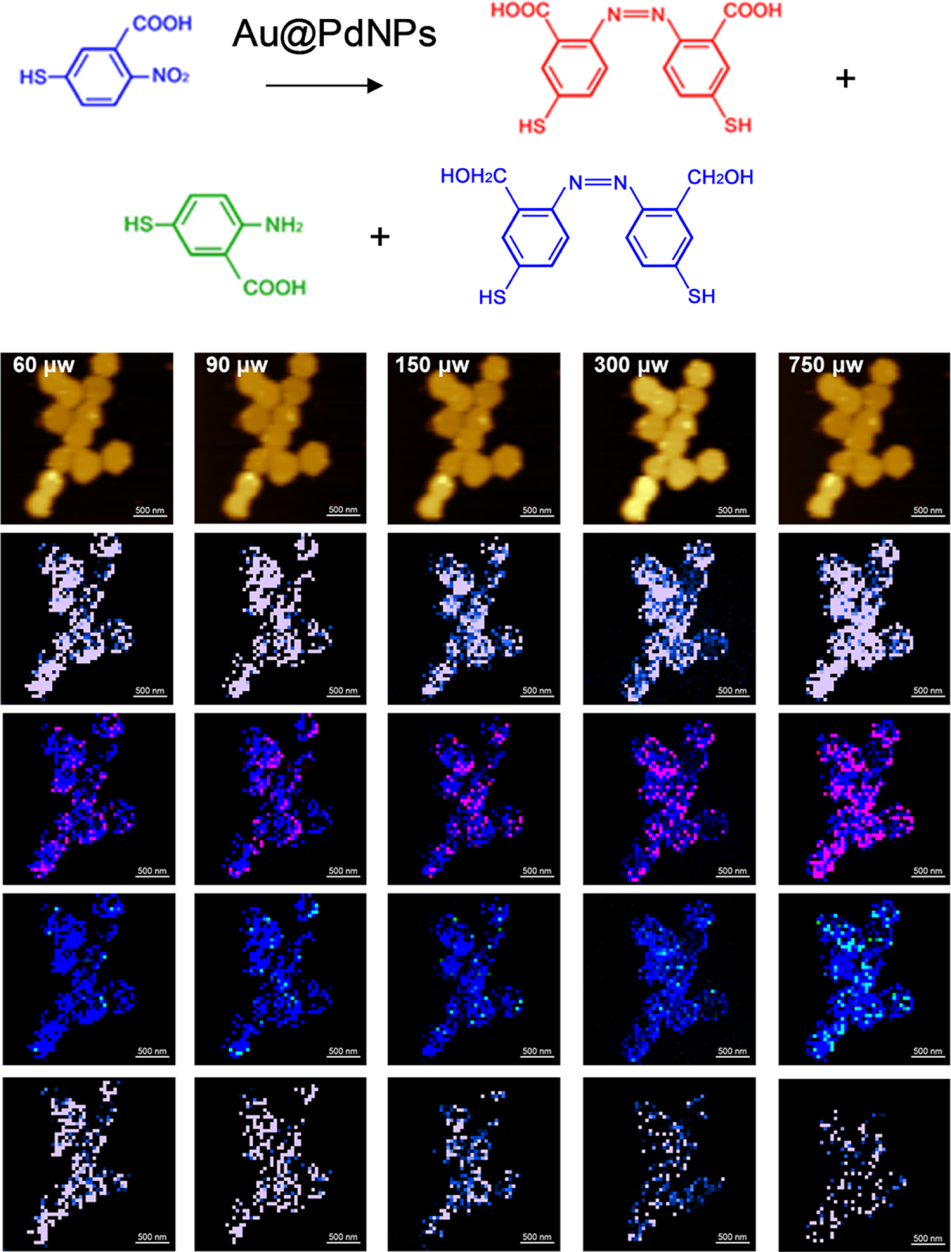
TERS imaging of 2-N-5TBA reduction on Au@PdNPs at different light intensities (60–1500 μW of 633 nm laser light). In situ AFM images (top row) of Au@PdNPs and corresponding TERS images of 4-NBT (1339 cm^–1^) shown in blue (middle row), as well as the overlapped TERS images of 4-NBT (blue), DMAB (1397 and 1441 cm^–1^) (red) and 4-ATP (1486 cm^–1^) (green; bottom row). Scale bars are 500 nm.

We also performed a TERS analysis of the plasmon-driven reduction of 2-N-5TBA on Au@PdNPs (Figure 8). In addition to product (3) on AuNPs, products (4) and (7) were observed. However, in contrast to the AuNPs, no indication regarding the formation of the products (1) and (2) was evident on Au@PdNPs. TERS was also used to examine the yield of products (3), (4) and (7) at different laser intensities (633 nm) see (Figure 2). We found a graduate increase in the yield of all three products with an increase in the light intensity. Specifically, the yield of the product (3) increased from 17.4% at 60 μW to 75.9% at 750 μW. Our results demonstrate a graduate increase in the yield of product (4) from 3.1% at 60 μW to 24.6% at 750 μW. Finally, a similar increase was observed for the product (7), which changed from 6.8% at 60 μW to 22.5% at 750 μW.

**Figure 9:**
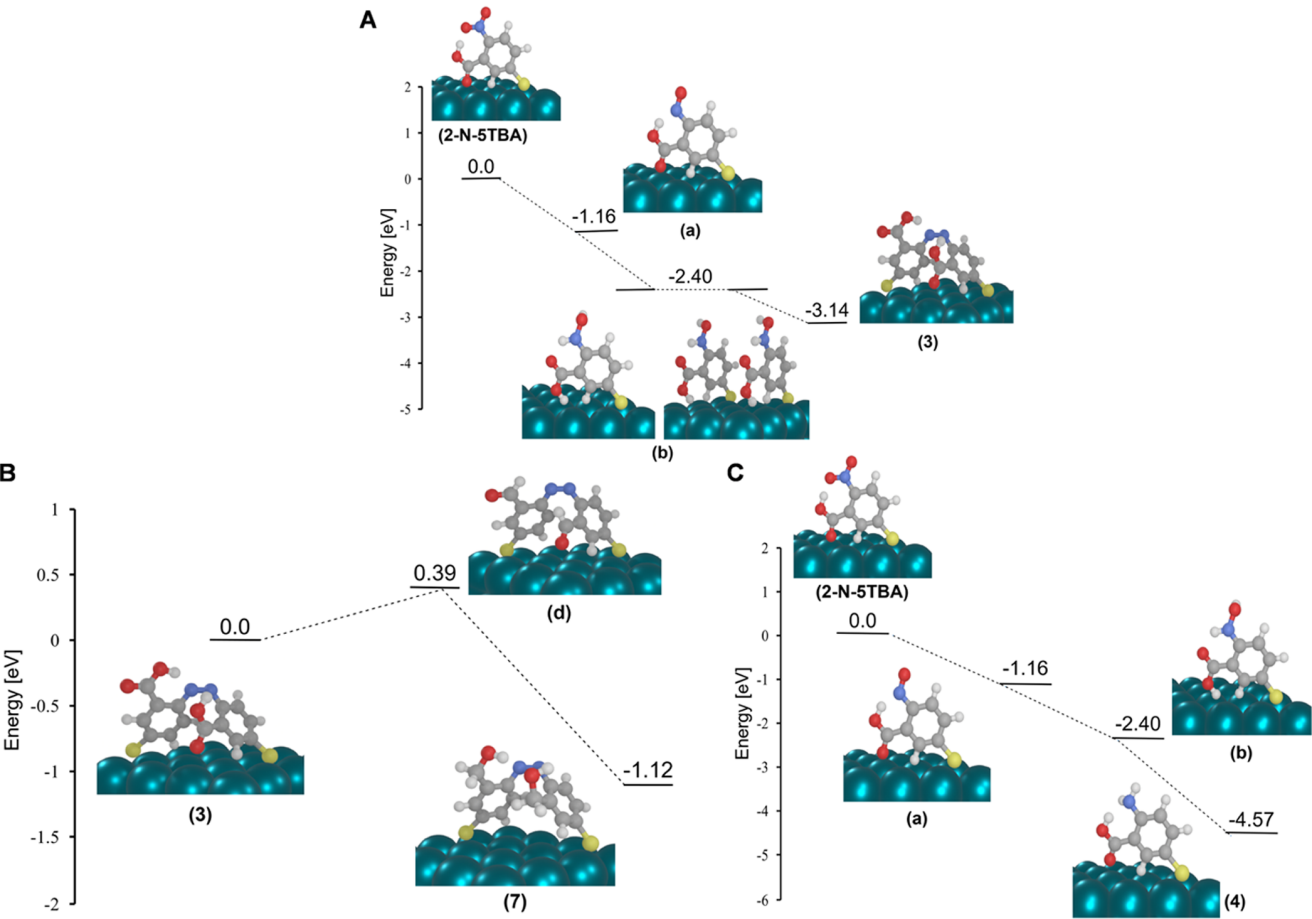
Ground-state reaction pathways on Pd surface for the formation of (A) product (3) from 2-N-5TBA; (B) product (4) form 2-N-5TBA; (C) product (7) from product (3) as predicted by periodic DFT calculations. The driving forces for each stepwise redox reaction are shown. Structures of the surface-immobilized intermediates formed in the different reaction pathways are illustrated.

In contrast to the theoretical insights obtained in the scope of AuNPs, a totally different scenario arises in the presence of Au@PdNPs. Zhang et al. already investigated the plasmon-induced hot-electron transfer at Au–metal interfaces and its effects on photocatalysis reactions using theoretical and experimental methods.^59^ A three-step mechanism was determined to be responsible for the transfer of hot electrons at the Au–Pd interface: Initially, hot electrons are generated at the Au core as a result of incident light excitation and are subsequently at the Au–Pd interface to the Pd shell. In order to trigger the reaction, hot electrons must migrate through the Pd shell and arrive at the surface. Since Pd has a continuous electronic band structure, hot electrons have a very short lifetime, resulting in short distances traveled. As a result, we expect to see less charge transfer between 2-N-5TBA and the slab while using Au@PdNPs in the experiment which hampers the efficiency of the decarboxylation reaction. Thus, reduction to the respective alcohol proceeds dominantly, which requires less charge transfer. As shown in Figure 9A, converting 2-N-5TBA to product (3) follows the equivalent thermodynamically feasible mechanism as discussed previously for AuNPs. Once the product (3) is formed, conversion to (7) via the reduction of the carboxyl group to the aldehyde and further to the alcohol follows (Figure 9B). In comparison to the driving force involved in the formation of product (3), the driving force associated with the generation of product (2) is smaller. Therefore, the preference of producing (3) over (2) is reasoned. Figure 9C demonstrates the reaction pathway of converting 2-N-5TBA to product (4). Based on the driving forces of all reaction pathways (Figure 9), it is tempting to conclude that (1) features a higher yield than the other reaction products, which is, however not in-line with the experimental findings. This at first glance apparent contradiction can be easily untangled as the product (1) can be converted into (3) by transferring charge from the molecule to Pd.

## Conclusions

Our experimental and theoretical results show that two chemical groups of the same molecular analyte exhibit drastically different reactivity in plasmon-driven reactions. We found that nitro groups are far more reactive than carboxyl groups of 2-N-5TBA. Our results showed that nitro groups reduced forming di-carboxylazobenzene (product 3), which then yielded DMAB (product (2)). Finally, the plasmon-driven split of DMAB yielded 4-NBT. These findings helped to explain the experimentally observed prevalence of di-carboxylazobenzene over DMAB and 4-NBT on AuNPs. Our findings also demonstrated that the presence of Pd on Au surface drastically changes catalytic pathways of plasmon-driven reduction of 2-N-5TBA. Specifically, di-carboxylazobenzene was found to form product (7), in which both carboxyl groups were reduced to alcohols (Scheme 1). Our results also showed that this reactant then split in a similar manner to DMAB forming 2-amino-3-mercaptobenzoic acid (product 4). Thus, 2-N-5TBA on AuNPs yielded 4-NBT (product 1), DMAB (product 2), and di-carboxylazobenzene (product 3). At the same time, this molecular analyte was transformed into di-carboxylazobenzene (product 3), 2-amino-3-mercaptobenzoic acid (product 4), and product (7) on Au@PdNPs.

## Supporting Information Available

Experimental details can be seen in the Supporting Information (PDF)
