# Supplementary material for: Plasmon-Determined Selectivity in Photocatalytic Transformations on Gold and Gold–Palladium Nanostructures
Source: ACS Photonics. 2023 Aug 30;10(9):3390–400. doi: 10.1021/acsphotonics.3c00893 (PMC10863388; doi:10.1021/acsphotonics.3c00893)
Supplement: Supplementary file 1 — ph3c00893_si_001.pdf [file ph3c00893_si_001.pdf]

Plasmon-Determined Selectivity in Photocatalytic Transformations on Gold and Gold-Palladium Nanostructures

Zhandong Li[1†], Sadaf Ehtesabi[2†], Siddhi Gojare[2], Martin Richter[2#], Stephan Kupfer[2], Stefanie Gräfe[2] and Dmitry Kurouski[1,3]*

[1]Department of Biochemistry and Biophysics, Texas A&M University, College Station, Texas 77843, United States
[2]Institute of Physical Chemistry and Abbe Center of Photonics, Friedrich Schiller University Jena, Helmholtzweg 4, 07743 Jena, Germany
[3]Department of Biomedical Engineering, Texas A&M University, College Station, Texas, 77843, USA

*Corresponding Author: Dmitry Kurouski; email: dkurouski@tamu.edu

†These authors contributed equally.

#Current address: DS Deutschland GmbH, Am Kabellager 11-13, 51063 Cologne, Germany

Supporting Information

Experimental Details.

Chemicals

Gold (III) chloride trihydrate ($HAuCl_4{\cdot}3H_2O$, 99.9%), Palladium (II) chloride solution (H_2PdCl_4), hexadecyltrimethylammonium bromide (CTAB, 99%), 4-nitrothiolphenol (4-NTP), sodium hydroxide (NaOH, 98%), potassium iodide (KI, 99%), L-ascorbic acid (AA, 99%), sodium borohydride ($NaBH_4$, 99%) were purchased from Sigma-Aldrich (St. Louis, MO). Sodium citrate dihydrate (Na-Cit, 99%) was purchased from Fisher scientific (Waltham, MA). Ethanol was purchased from Decon Labs (King of Prussia, PA). All chemicals were used as received without purification. 2-nitro-5-thiolobenzoic acid (2-N-5TBA) or 5-Mercapto-2-nitro-benzoic acid was purchased from PrepChem.

Preparation of the AuNPs and Au@PdNPs.

The AuNPs were prepared by first seed-mediated growth method following by the isotropical growth. Au seeds solution was first prepared by add 0.01 M, 1 mL of $HAuCl_4$ and 0.01 M, 1 mL of Na-Cit solutions into 36 mL of water. Then, under vigorous stirring, 0.1 M, 1 mL of ice-cold $NaBH_4$ solution was introduced and kept stirring for 2 min. Next, the mixture solution was kept at room temperature without disturbing, aging for 2-6 h. Au nanoplates (AuNPs) seed solution with ~15 nm thickness with triangle or hexagonal was then prepared by three-step seed-mediated growth of the Au seeds after aging. Briefly, the growing solutions 1, 2 and 3 were prepared as following methods. Firstly, growing solution 1 and 2 were prepared the same where 0.01 M, 0.25 mL of $HAuCl_4$, 0.1 M, 0.05

mL of NaOH, 0.01 M, 0.05 mL of KI, and 0.1 M, 0.05 mL of Ascorbic Acid (AA) was added into 0.05 M, 9 mL of CTAB solution by order. Then, the third growing solution was prepared similarly by introducing 0.01 M, 2.5 mL of $HAuCl_4$, 0.1 M, 0.5 mL of NaOH, 0.01 M, 0.5 mL of KI, and 0.1 M, 0.5 mL of AA into 0.05 M, 90 mL of CTAB solution. Subsequently, 1 mL of the Au seeds stocking solution was added into growing solution 1, followed by gently shaking for 5 s. Then, 1 mL of the Au seeds and growing solution 1 mixture were added to the growth solution 2, followed by gently shaking for another 5 s. Finally, all of the Au seeds and growth solution 1, 2 mixtures were added to the growing solution 3 following by gently shaking for 5 s. The final solution was then kept at room temperature without any disturbing for overnight. Then, the ~15 nm thickness AuNPs seeds were collected by precipitating at 5000 rpm for 2 min and dissolved in 5 mL of CTAB solution for the next step of growth. The thicker Au nanoplates (AuNPs) were then synthesized by conducting isotropical growth on the ~15 nm thickness AuNPs in diluted CTAB solution. For isotropical growth, briefly, the growth solution was prepared by mixing 0.25 M, 1 mL of $HAuCl_4$, 0.1 M, 0.055 mL of AA and water (8 mL) with 0.1 M, 1 mL of CTAB solution by order. Then, the isotropical growth reaction was initialized by adding 0.3 ml of the AuNPs seeds solution (15 nm) into the above freshly prepared isotropical growth solution. After this step, the expecting thickness of AuNPs was at least 60 nm. Next, Au@PdNPs were prepared by this recipe, 250 µL of the AuNPs (60 nm) stock solution was first mixed with 60 µL of 20 mM AA. Subsequently, the solution was brought to be mixed by vortexing for 10 s. Next, 15 µL, 10 mM of H_2PdCl_4 solution was introduced following by vortexing for 10 s again. The solution was kept at room temperature for 1 h without disturbing until the completion of bimetallic nano-plates growth. This synthetic procedure results in the formation of sub-monolayer of Pd on the surface of AuNPs (doi.org/10.1021/acsphotonics.1c00561; Figures S5 and S6). For purification, the solution was centrifuged at 8000 rcf 2 min for twice. Finally, after removal of the supernatant, the Au@PdNPs were dissolved in 1.0 mL of water and sonicated for 20 s.

Formation of the 2-N-5TBA monolayer on the AuNPs and Au@PdNPs. A drop of the as-synthesized nano-particle stock solution was first deposited on precleaned Si wafer, incubated for 0.5 h. Subsequently, the nanoplates deposited Si wafer was immersed in a 2 mM ethanolic solution of 2-N-5TBA solution for 1 h to form monolayer of 2-N-5TBA on different type of particles. Finally, the modified sample was sonicated in ethanol for 3 min for removing the uncoordinated 2-N-5TBA molecules.

TERS Probe Fabrication Silicon AFM probes with related parameters force constant 2.7 N/m and resonance frequency 50-80 kHz were purchased from Appnano (Mountain View, CA). Then, the metal evaporation was carried out for coating the AFM tips with a layer of gold. Briefly, two of the probes were fixed onto each of the clamped device and ten of the devices were put in the thermal evaporator chamber (MBrown, Stratham, NH). During metal deposition, the pressure was kept at $\sim 1\times10^{-6}$ mbar. Then, gold pellets (Kurt J. Lesker, Efferson Hills, PA) thermally evaporate at constant 0.2 $A\cdot s^{-1}$ rate. After 70 nm of Au was deposited on the AFM tips, the evaporation was stopped and kept cooling down to room temperature. The temperature at the tip surface and deposition chamber was ~ 50 °C.

TERS measurement AFM-TERS and AFM scanning was carried out on the AIST-NT-HORIBA system equipped with a 632.8 nm continuous wavelength (CW) laser. Laser light was brought to the sample surface in a side-illumination geometry with a 100X Mitutoyo microscope objective. The scattering electromagnetic radiation was also collected with the same objective and directed introduced to a fiber-coupled Horiba iHR550 spectrograph that equipped with a Synapse EM-CCD camera (Horiba, Edison, NJ).

Ground state simulations

Quantum chemical simulations were performed in order to elucidate the mechanism underlying the plasmon-induced conversion from 2-nitro-5-thiolobenzoic acid (2-N-5TBA) branching into eight different products at the molecular level. Therefore, all geometry optimizations for the singlet ground states of educt (2-N-5TBA), intermediates, and products (1-8) surface-immobilized on an Au or a Pd cluster, respectively, were performed at the density functional level of theory (DFT). Periodic DFT calculations were based on the projector-augmented wave (PAW) method employing the optB88-vdW functional in a real-space grid of 0.2 Å resolution, implemented in the GPAW program package in cooperation with ASE interface.[1-4] Au and Pd slabs are represented by a 4×4×3 fcc(111) cluster, resulting in 3 layers each of 16 atoms using an optimized lattice constant of 4.178 and 3.957 Å, respectively. All molecules were anchored via the strongly bonding sulfur atom of the thiol-moiety to the respective metal cluster. The subsequent partial structural relaxation was performed employing two-dimensional periodic boundary conditions (*x*- and *y*-direction), while second and third layers of the metal slab were frozen to reduce computational costs.

In the case of the immobilized substrates comprising merely one aromatic ring, several conformations are investigated, *e.g.*, with respect to the relative orientation of the substituents with respect to the metallic surface. In addition, we predict a pronounced impact on the orientation with respect to the surface coverage. Hence, in case of high surface coverage, where in addition to the *i*) strong chemical interaction arising from the sulfur-metal bond, weaker *ii*) substrate-metal interactions based on physisorption, as well as on *iii*) dispersive interactions between the substrate molecules, favor configurations with perpendicular orientation of the aromatic planes relative to the metal surface. Regarding the 2-N-5TBA educt, two configurations of the respective hybrid system were investigated, in which the aromatic plane of the molecule is orthogonal to the metal surface (Figure S1). In the first orientation – denoted 2-N-5TBA$_1$ – the ^{1}C's hydrogen atom and the oxygen atom of the carboxyl group interact with the metal surface, while in the second orientation – denoted 2-N-5TBA$_2$ – the ^{5}C's hydrogen is responsible for the interaction. Since the 2-N-5TBA$_1$ orientation was predicted to be energetically favored with respect to 2-N-5TBA$_2$ by 0.23 and 0.46 eV on Au and Pd cluster, all calculations were performed exclusively for 2-N-5TBA$_1$. Product (4) was oriented in a similar fashion as 2-N-5TBA$_1$; however, instead of the oxygen atom of the carboxyl group interacting with the surface, the OH interacting with it (Figure S2). For product (1), we focused our simulations on an orientation in which ^{1}C's hydrogen atom interacts with the metal surface. In order to evaluate the dispersive intermolecular interactions among neighboring surface-immobilized substrate molecules in more detail, two conformers with parallel as well as with antiparallel orientation were assessed. The simulations of the educt on the gold surface revealed that parallel structures are approximately 0.08 eV more favorable in

comparison to antiparallel orientations (Figure S3). Consequently, parallel structures were employed in all following calculations. As the dimerization, yielding products (2), (3), and (7), is restricted to neighboring molecules, the structure of these products features a more pronounced degree of rigidity, *e.g.*, the strong metal-sulfur bond of the monomers allows exclusively the formation of the cis-isomer. Therefore, only cis-isomers were investigated for products (2) and (3). In product (3), two cis-isomer configurations were investigated (Figure S4), namely the thermodynamically favored structure (by 0.20 and 0.15 eV on Au and Pd surface, respectively) with both carboxyl groups pointing to the same direction (3_1) and the respective structure with the two carboxyl groups pointing in opposing directions (3_2). In contrast, product (7) has an energetically more favorable configuration with the two hydroxymethyl groups pointing in opposing directions (7_2) than the configuration with both groups pointing to the same side (7_1) (Figure S5).

Excited state simulations

Subsequently, non-periodic DFT and time-dependent DFT (TDDFT) simulations were performed using Gaussian 16 program.[5] Frequency calculations were carried out for each hybrid system (educt, products, and intermediates) at the CAM-B3LYP/def2-tzvp level of theory while the metal cluster was frozen.[6-8] These calculations not only confirmed that a minimum was obtained on the 3*N*-6-dimensional potential energy surface (PES), but also provided key thermochemical data (pressure of 1 atm and temperature of 298.15 K).
Furthermore, the electronic nature of light-driven processes in resonance upon 633-nm photoexcitation (1.96 eV) of the plasmonic hybrid system(s) were investigated at the TDDFT level of theory. In particular, the photoinduced redox chemistry between the metal cluster (Au or Pd) and the respective substrate (educt, intermediate, product state) were assessed. Therefore, the same XC functional was applied as for the preliminary ground state calculations, while the basis set was reduced to def2-svp. Such computational approach allows an adequate description of excited state properties of azobenzenes, as shown lately in comparison with high level multiconfigurational methods, i.e., with respect to excitation energies, excited state gradients and resonance Raman intensities. Electronic characters, *i.e.* local excitation of the substrate as well as charge transfer excitation between the substrate and the metal cluster, were evaluated based on charge density differences (CDDs) (See Table S1-S7).

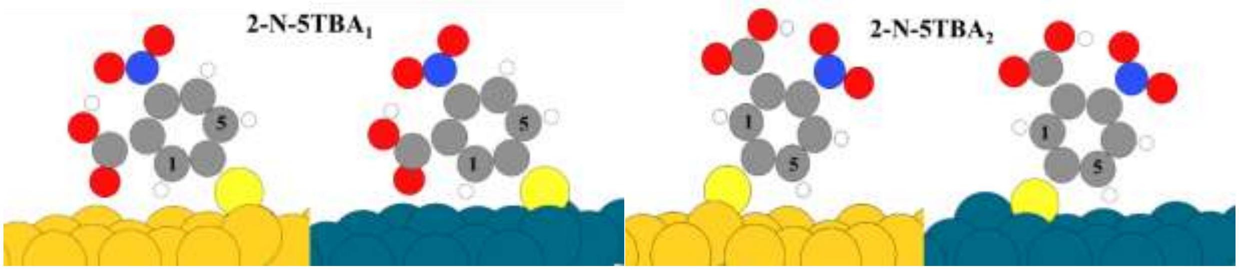

Figure S1. Two 2-N-5TBA configurations. In the first orientation – denoted 2-N-5TBA$_1$ – the ^{1}C's hydrogen atom and the oxygen atom of the carboxyl group interact with the metal surface. In the second orientation – denoted 2-N-5TBA$_2$ – the ^{5}C's hydrogen is responsible for the interaction. 2-N-5TBA$_1$ was predicted to be energetically favored with respect to 2-N-5TBA$_2$ by 0.23 and 0.46 eV on Au and Pd cluster.

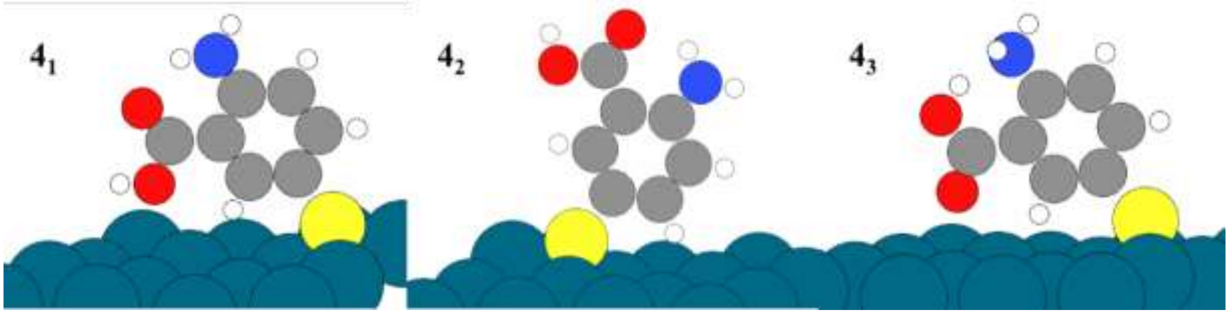

Figure S2. different configurations of product (4) on Pd cluster.

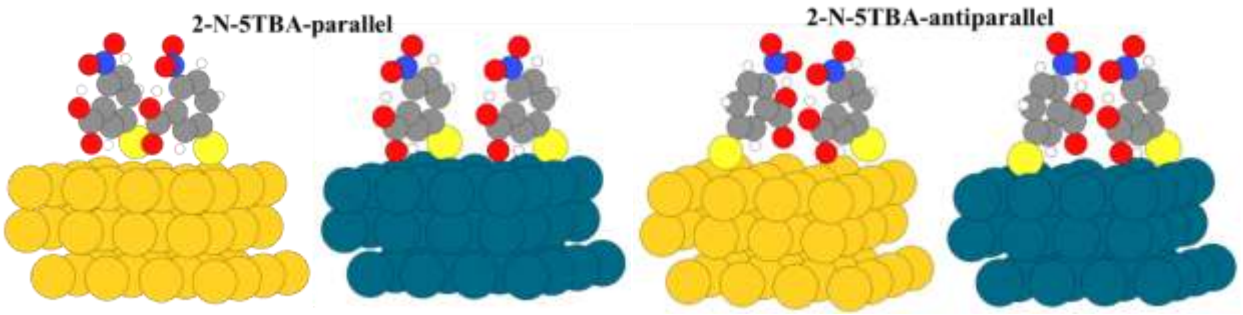

Figure S3. Two 2-N-5TBA conformers with parallel and antiparallel orientation. The simulations of the educt on gold surface revealed that parallel structures are approximately 0.08 eV more favorable in comparison to antiparallel orientations.

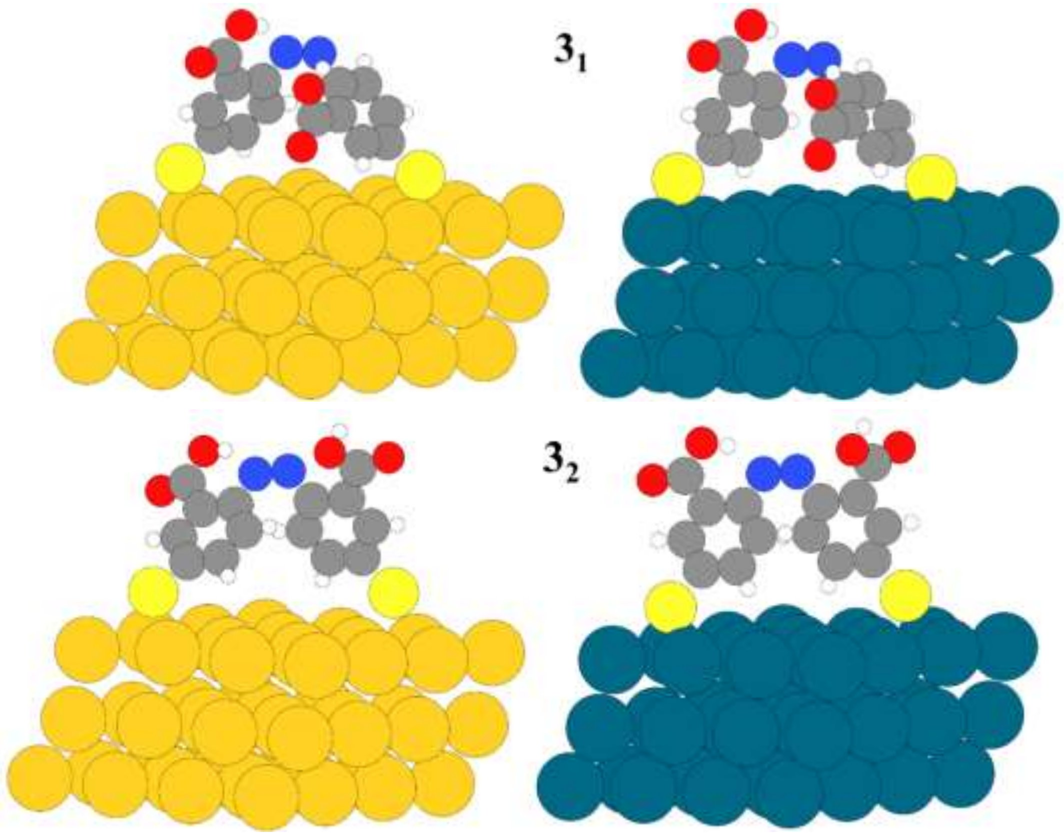

Figure S4. Cis-isomer configurations of product (3). 3_1 with both carboxyl groups pointing the same direction and 3_2 with the two carboxyl groups pointing in opposing directions.

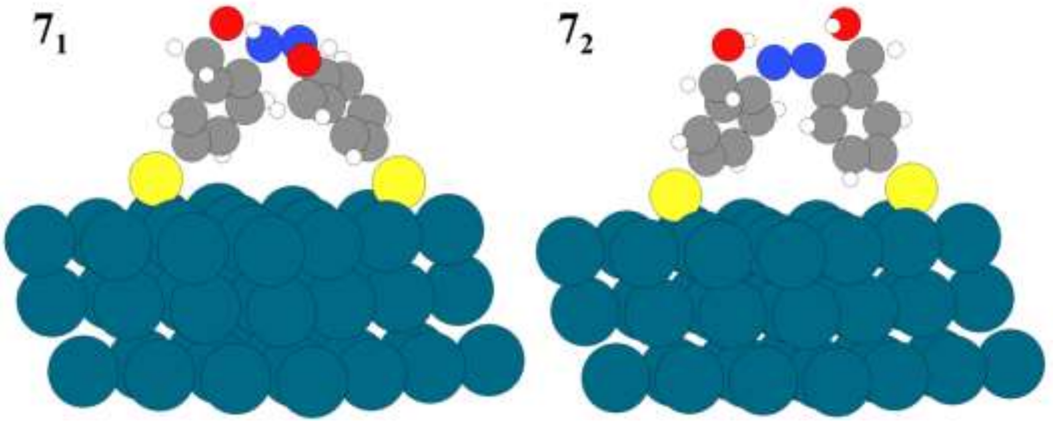

Figure S5. Cis-isomer configurations of product (7). 7_1 with both hydroxymethyl groups pointing to the same side and 7_2 with the two hydroxymethyl groups pointing in opposing direction.

Table S1: Charge density differences (CDDs) illustrating the nature of the low-lying bright excitations of 2-N-5TBA. Charge transfer takes place from red to blue.

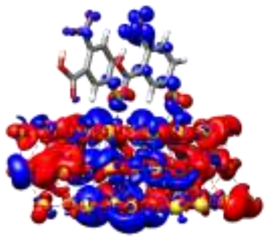<br>State 36<br>Energy: 1.406 eV<br>Osc.: 0.017	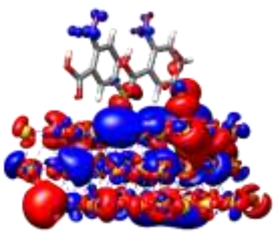<br>State 48<br>Energy: 1.641 eV<br>Osc.: 0.022	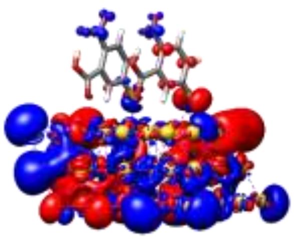<br>State 49<br>Energy: 1.663 eV<br>Osc.: 0.021	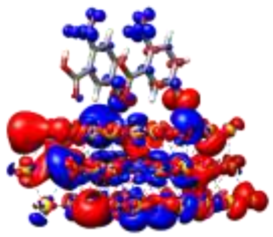<br>State 52<br>Energy: 1.730 eV<br>Osc.: 0.027
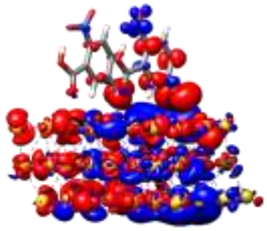<br>State 53<br>Energy: 1.738 eV<br>Osc.: 0.010	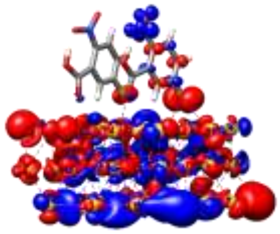<br>State 58<br>Energy: 1.829 eV<br>Osc.: 0.019	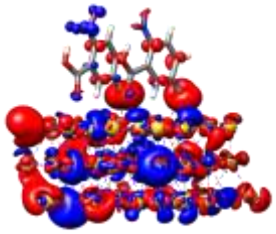<br>State 59<br>Energy: 1.834 eV<br>Osc.: 0.010	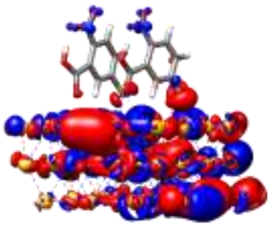<br>State 62<br>Energy: 1.886 eV<br>Osc.: 0.011
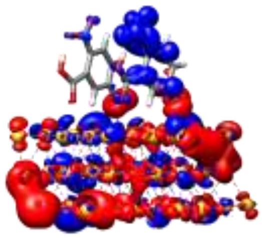<br>State 67<br>Energy: 1.951 eV<br>Osc.: 0.010	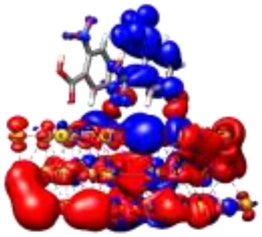<br>State 69<br>Energy: 1.974 eV<br>Osc.: 0.003	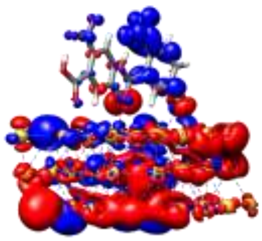<br>State 72<br>Energy: 2.007 eV<br>Osc.: 0.003	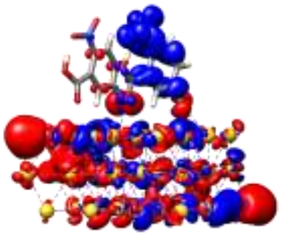<br>State 73<br>Energy: 2.026 eV<br>Osc.: 0.004

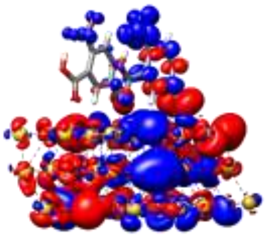<br>State 77<br>Energy: 2.079 eV<br>Osc.: 0.036	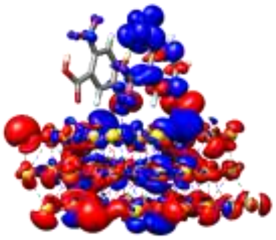<br>State 88<br>Energy: 2.207 eV<br>Osc.: 0.041	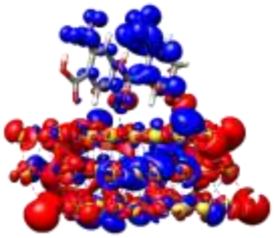<br>State 89<br>Energy: 2.224 eV<br>Osc.: 0.016	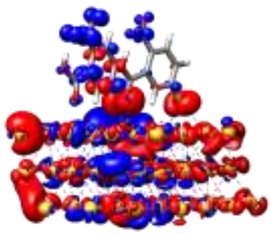<br>State 90<br>Energy: 2.241 eV<br>Osc.: 0.035
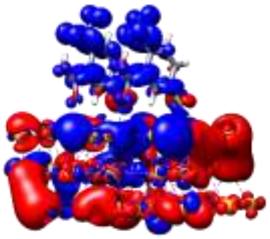<br>State 91<br>Energy: 2.253 eV<br>Osc.: 0.047	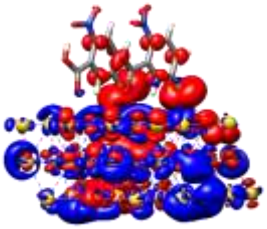<br>State 94<br>Energy: 2.283 eV<br>Osc.: 0.022	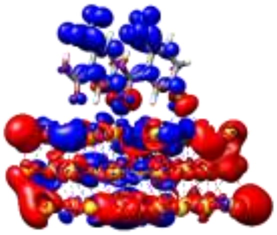<br>State 95<br>Energy: 2.299 eV<br>Osc.: 0.014	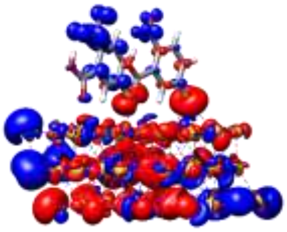<br>State 97<br>Energy: 2.330 eV<br>Osc.: 0.028
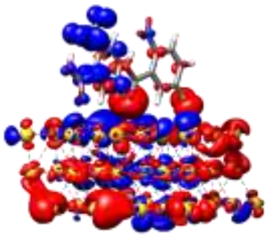<br>State 98<br>Energy: 2.336 eV<br>Osc.: 0.037	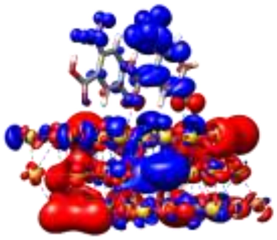<br>State 103<br>Energy: 2.401 eV<br>Osc.: 0.021	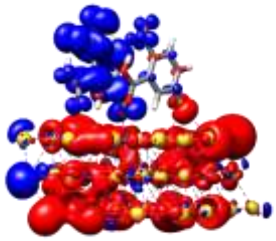<br>State 105<br>Energy: 2.421 eV<br>Osc.: 0.050	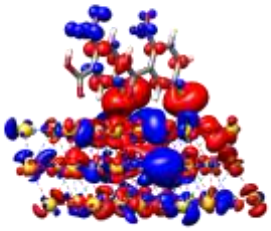<br>State 107<br>Energy: 2.438 eV<br>Osc.: 0.019

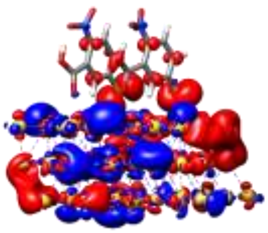<br>State 110<br>Energy: 2.479 eV<br>Osc.: 0.021	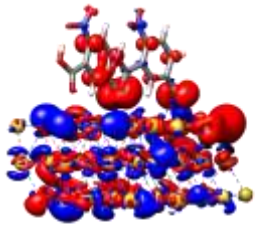<br>State 111<br>Energy: 2.489 eV<br>Osc.: 0.016	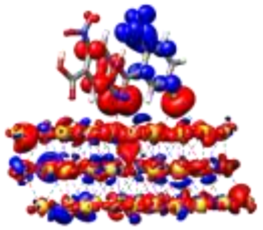<br>State 115<br>Energy: 2.542 eV<br>Osc.: 0.033	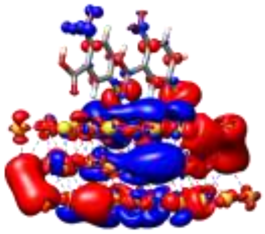<br>State 116<br>Energy: 2.548 eV<br>Osc.: 0.031
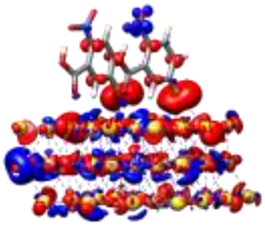<br>State 117<br>Energy: 2.561 eV<br>Osc.: 0.020	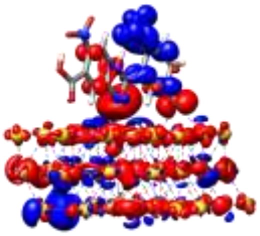<br>State 120<br>Energy: 2.588 eV<br>Osc.: 0.040	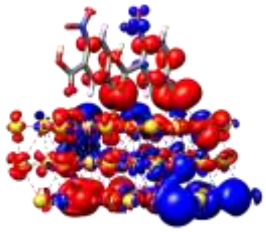<br>State 121<br>Energy: 2.598 eV<br>Osc.: 0.029	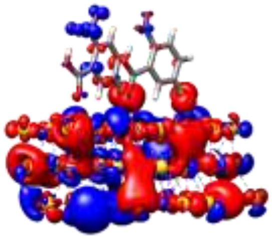<br>State 122<br>Energy: 2.600 eV<br>Osc.: 0.066
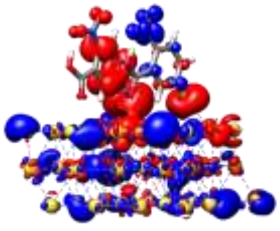<br>State 123<br>Energy: 2.619 eV<br>Osc.: 0.019	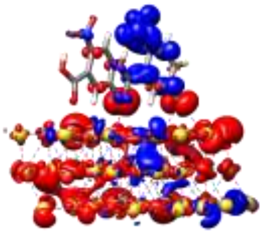<br>State 124<br>Energy: 2.622 eV<br>Osc.: 0.060	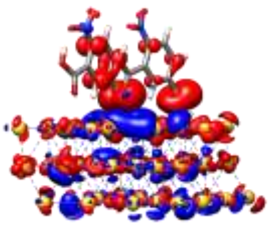<br>State 126<br>Energy: 2.629 eV<br>Osc.: 0.039	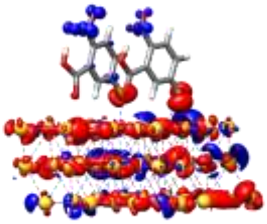<br>State 128<br>Energy: 2.655 eV<br>Osc.: 0.185

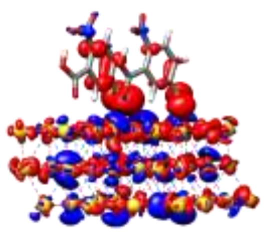<br>State 129<br>Energy: 2.664 eV<br>Osc.: 0.011	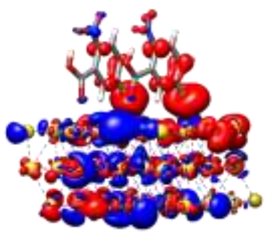<br>State 130<br>Energy: 2.669 eV<br>Osc.: 0.019	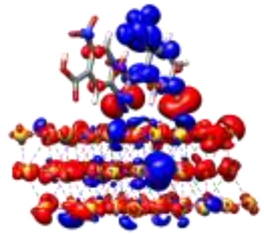<br>State 132<br>Energy: 2.692 eV<br>Osc.: 0.012	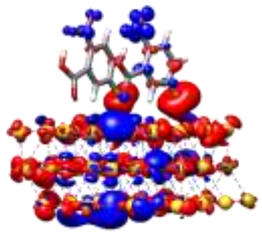<br>State 133<br>Energy: 2.696 eV<br>Osc.: 0.010
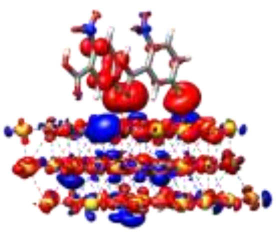<br>State 134<br>Energy: 2.710 eV<br>Osc.: 0.041	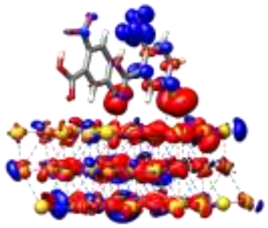<br>State 136<br>Energy: 2.733 eV<br>Osc.: 0.132	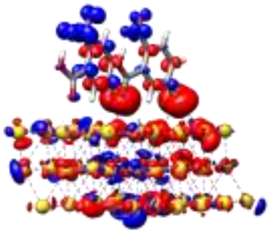<br>State 137<br>Energy: 2.736 eV<br>Osc.: 0.023	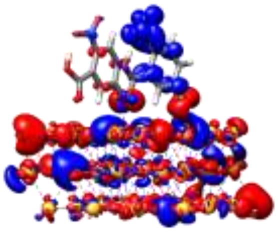<br>State 138<br>Energy: 2.739 eV<br>Osc.: 0.056
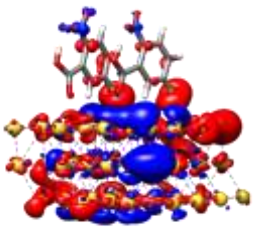<br>State 139<br>Energy: 2.742 eV<br>Osc.: 0.013	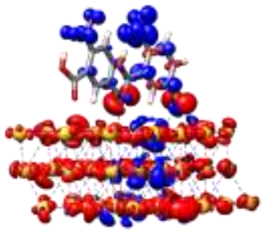<br>State 140<br>Energy: 2.754 eV<br>Osc.: 0.018	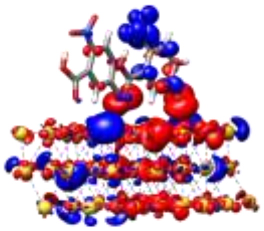<br>State 141<br>Energy: 2.761 eV<br>Osc.: 0.033	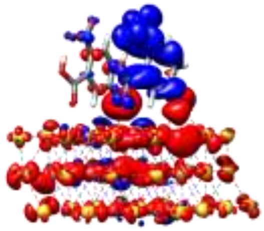<br>State 142<br>Energy: 2.772 eV<br>Osc.: 0.069

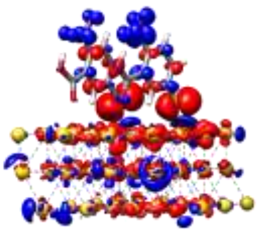

State 143
Energy: 2.781 eV
Osc.: 0.011

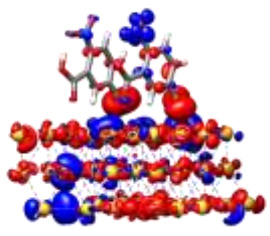

State 144
Energy: 2.788 eV
Osc.: 0.037

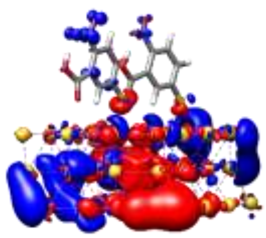

State 145
Energy: 2.800 eV
Osc.: 0.077

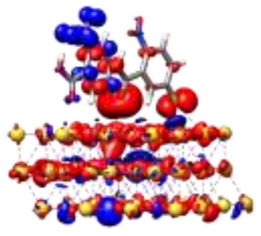

State 146
Energy: 2.807 eV
Osc.: 0.045

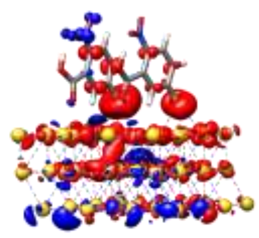

State 147
Energy: 2.821 eV
Osc.: 0.121

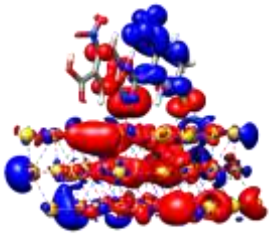

State 148
Energy: 2.825 eV
Osc.: 0.031

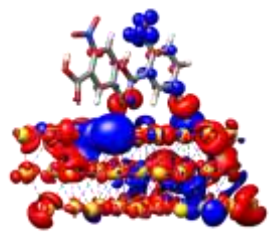

State 150
Energy: 2.836 eV
Osc.: 0.026

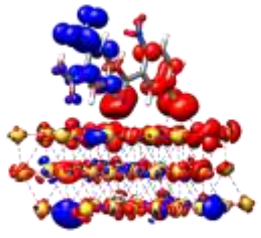

State 151
Energy: 2.845 eV
Osc.: 0.122

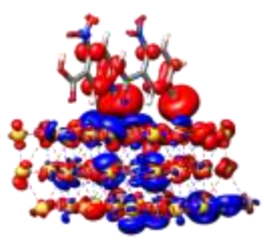

State 154
Energy: 2.865 eV
Osc.: 0.055

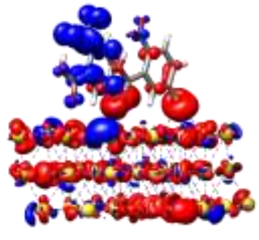

State 155
Energy: 2.867 eV
Osc.: 0.060

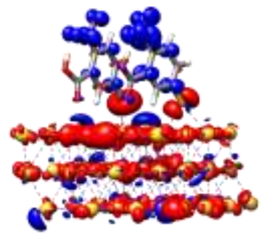

State 156
Energy: 2.873 eV
Osc.: 0.131

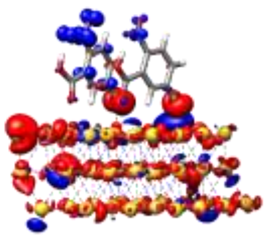

State 157
Energy: 2.885 eV
Osc.: 0.093

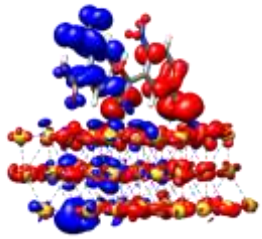<br>State 158<br>Energy: 2.890 eV<br>Osc.: 0.053	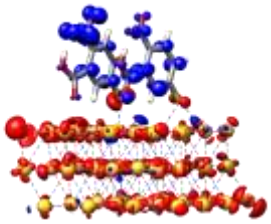<br>State 159<br>Energy: 2.900 eV<br>Osc.: 0.033	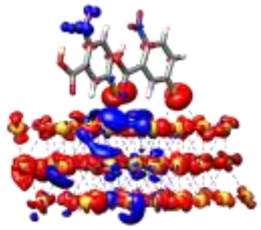<br>State 160<br>Energy: 2.913 eV<br>Osc.: 0.043	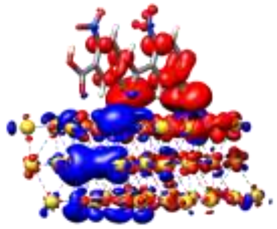<br>State 161<br>Energy: 2.927 eV<br>Osc.: 0.011
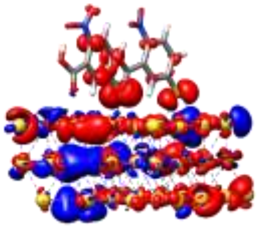<br>State 162<br>Energy: 2.929 eV<br>Osc.: 0.039	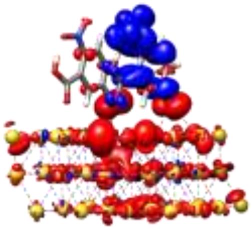<br>State 164<br>Energy: 2.940 eV<br>Osc.: 0.046	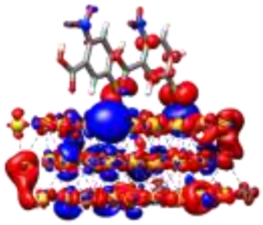<br>State 165<br>Energy: 2.947 eV<br>Osc.: 0.078	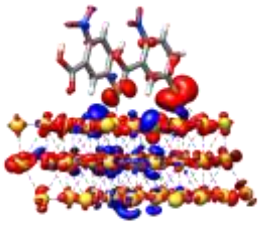<br>State 166<br>Energy: 2.957 eV<br>Osc.: 0.166
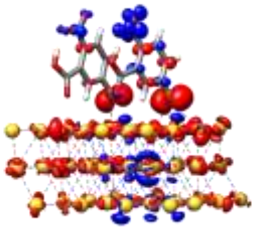<br>State 167<br>Energy: 2.970 eV<br>Osc.: 0.227	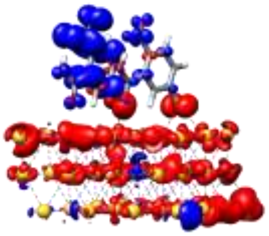<br>State 168<br>Energy: 2.977 eV<br>Osc.: 0.017	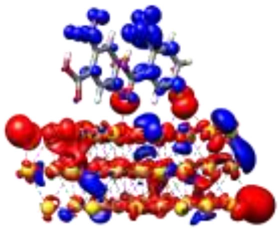<br>State 169<br>Energy: 2.977 eV<br>Osc.: 0.099	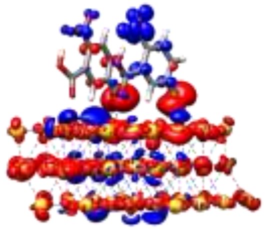<br>State 170<br>Energy: 2.984 eV<br>Osc.: 0.064

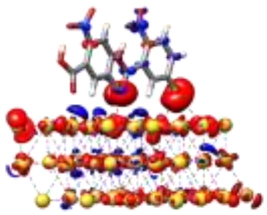 State 171<br>Energy: 2.991 eV<br>Osc.: 0.029			

Table S2: Charge density differences (CDDs) illustrating the nature of the low-lying bright excitations of product 1 (NTP). Charge transfer takes place from red to blue.

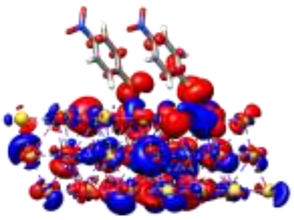<br>State 46<br>Energy: 1.580 eV<br>Osc.: 0.019	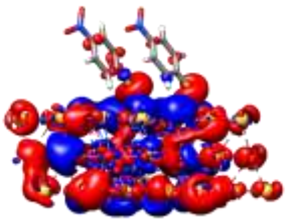<br>State 50<br>Energy: 1.660 eV<br>Osc.: 0.011	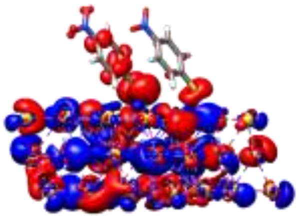<br>State 53<br>Energy: 1.708 eV<br>Osc.: 0.014	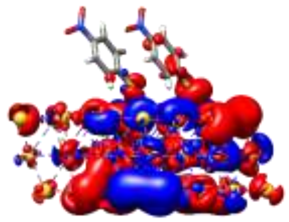<br>State 57<br>Energy: 1.769 eV<br>Osc.: 0.010
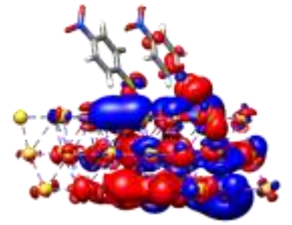<br>State 59<br>Energy: 1.795 eV<br>Osc.: 0.015	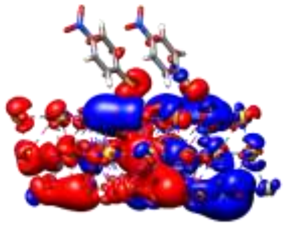<br>State 62<br>Energy: 1.841 eV<br>Osc.: 0.035	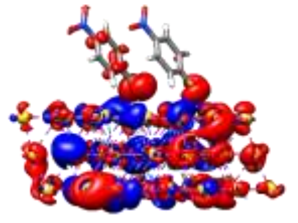<br>State 63<br>Energy: 1.873 eV<br>Osc.: 0.034	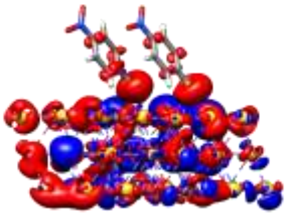<br>State 67<br>Energy: 1.925 eV<br>Osc.: 0.014
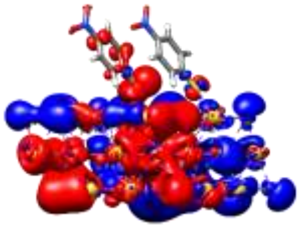<br>State 69<br>Energy: 1.941 eV<br>Osc.: 0.014	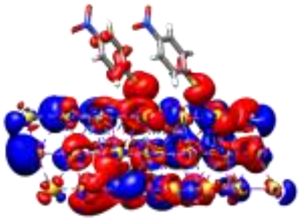<br>State 70<br>Energy: 1.970 eV<br>Osc.: 0.010	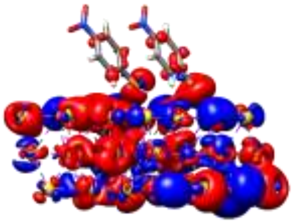<br>State 72<br>Energy: 2.002 eV<br>Osc.: 0.010	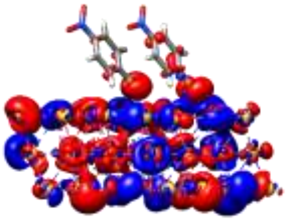<br>State 74<br>Energy: 2.032 eV<br>Osc.: 0.015

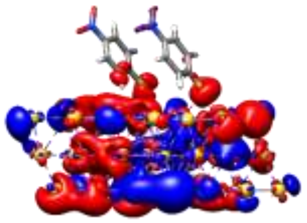

State 79
Energy: 2.088 eV
Osc.: 0.014

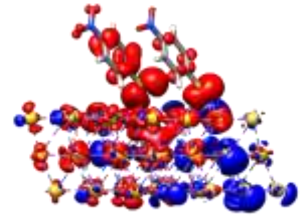

State 84
Energy: 2.157 eV
Osc.: 0.053

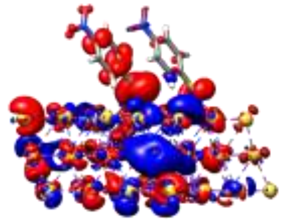

State 85
Energy: 2.174 eV
Osc.: 0.017

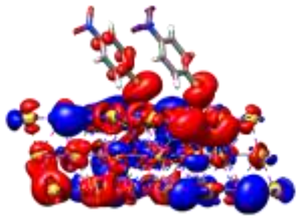

State 87
Energy: 2.207 eV
Osc.: 0.018

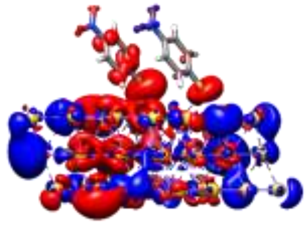

State 91
Energy: 2.248 eV
Osc.: 0.020

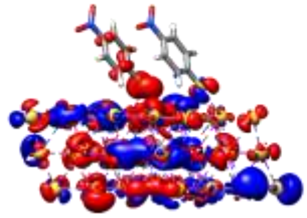

State 96
Energy: 2.312 eV
Osc.: 0.025

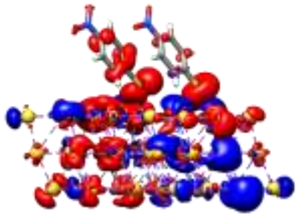

State 99
Energy: 2.352 eV
Osc.: 0.012

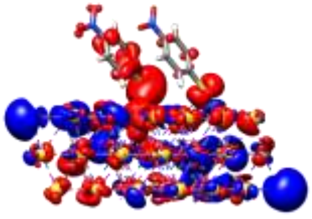

State 103
Energy: 2.404 eV
Osc.: 0.032

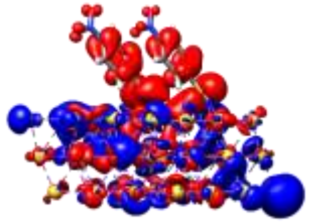

State 104
Energy: 2.411 eV
Osc.: 0.021

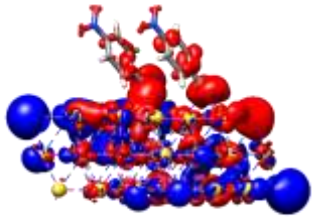

State 106
Energy: 2.442 eV
Osc.: 0.045

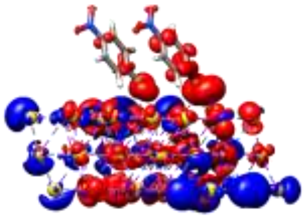

State 111
Energy: 2.484 eV
Osc.: 0.016

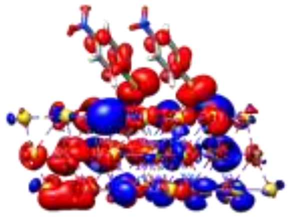

State 113
Energy: 2.501 eV
Osc.: 0.015

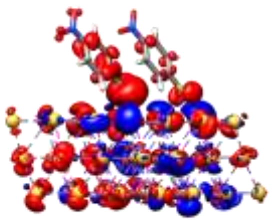

State 114
Energy: 2.512 eV
Osc.: 0.016

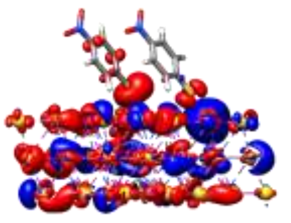

State 115
Energy: 2.522 eV
Osc.: 0.042

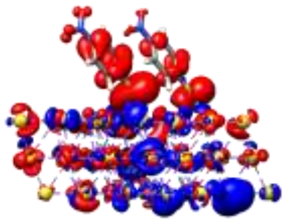

State 117
Energy: 2.538 eV
Osc.: 0.029

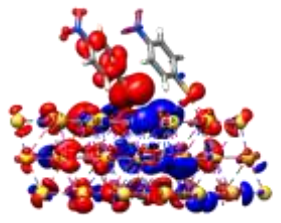

State 118
Energy: 2.546 eV
Osc.: 0.028

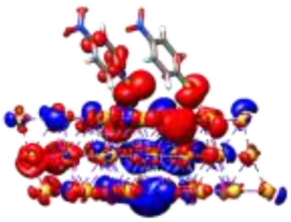

State 119
Energy: 2.559 eV
Osc.: 0.015

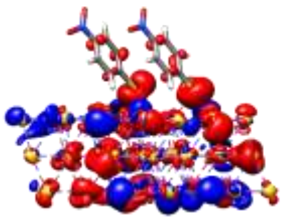

State 120
Energy: 2.562 eV
Osc.: 0.012

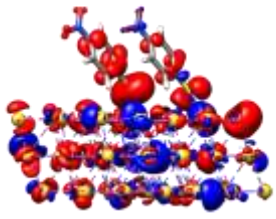

State 122
Energy: 2.585 eV
Osc.: 0.021

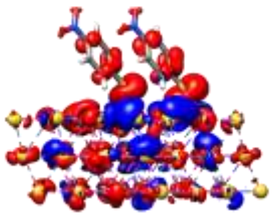

State 125
Energy: 2.616 eV
Osc.: 0.016

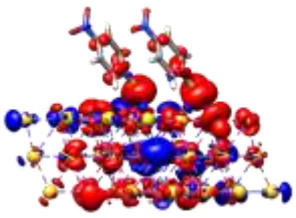

State 128
Energy: 2.649 eV
Osc.: 0.086

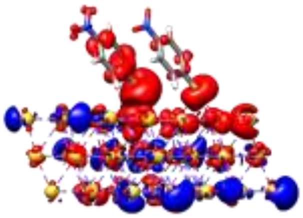

State 130
Energy: 2.675 eV
Osc.: 0.018

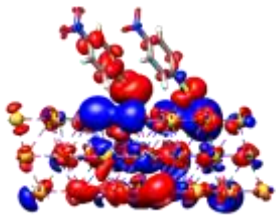

State 132
Energy: 2.696 eV
Osc.: 0.044

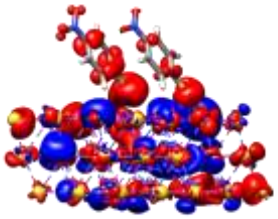

State 133
Energy: 2.698 eV
Osc.: 0.044

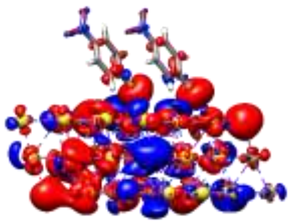<br>State 134<br>Energy: 2.717 eV<br>Osc.: 0.034	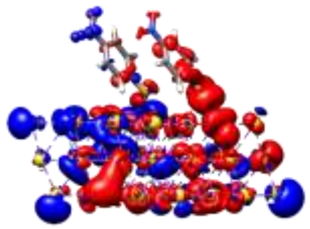<br>State 135<br>Energy: 2.723 eV<br>Osc.: 0.040	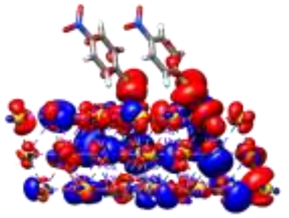<br>State 136<br>Energy: 2.731 eV<br>Osc.: 0.082	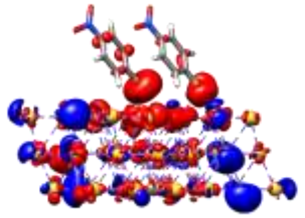<br>State 137<br>Energy: 2.737 eV<br>Osc.: 0.131
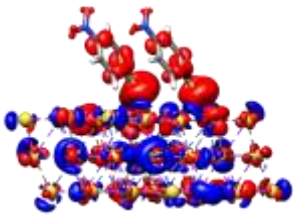<br>State 138<br>Energy: 2.745 eV<br>Osc.: 0.036	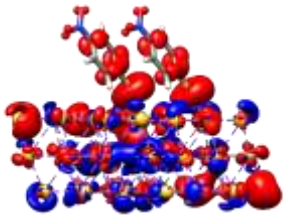<br>State 141<br>Energy: 2.770 eV<br>Osc.: 0.057	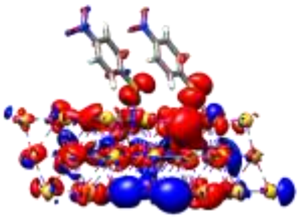<br>State 142<br>Energy: 2.774 eV<br>Osc.: 0.012	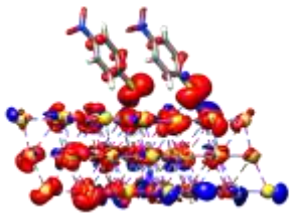<br>State 143<br>Energy: 2.790 eV<br>Osc.: 0.093
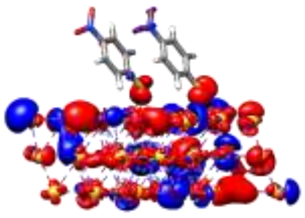<br>State 144<br>Energy: 2.812 eV<br>Osc.: 0.188	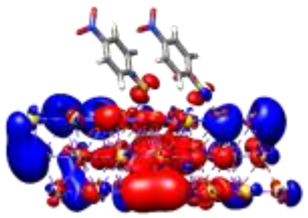<br>State 145<br>Energy: 2.819 eV<br>Osc.: 0.024	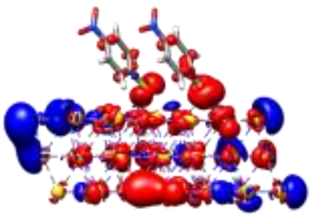<br>State 146<br>Energy: 2.823 eV<br>Osc.: 0.055	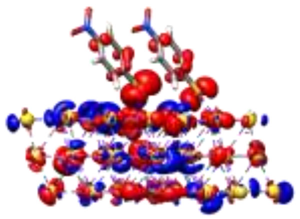<br>State 147<br>Energy: 2.831 eV<br>Osc.: 0.073

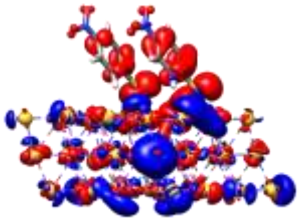<br>State 148<br>Energy: 2.838 eV<br>Osc.: 0.033	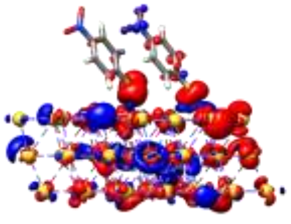<br>State 149<br>Energy: 2.846 eV<br>Osc.: 0.156	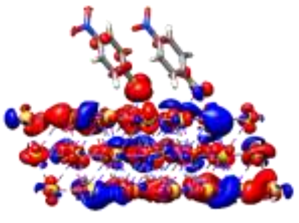<br>State 150<br>Energy: 2.855 eV<br>Osc.: 0.012	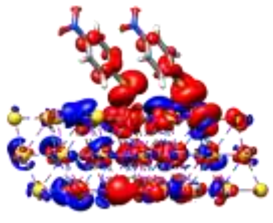<br>State 151<br>Energy: 2.857 eV<br>Osc.: 0.027
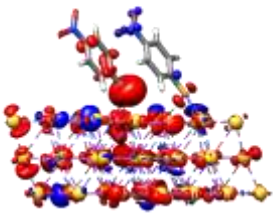<br>State 153<br>Energy: 2.880 eV<br>Osc.: 0.315	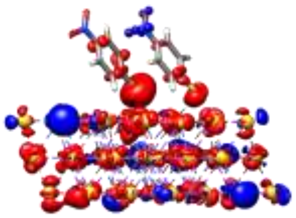<br>State 154<br>Energy: 2.891 eV<br>Osc.: 0.011	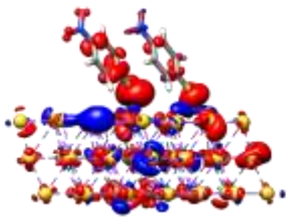<br>State 155<br>Energy: 2.898 eV<br>Osc.: 0.063	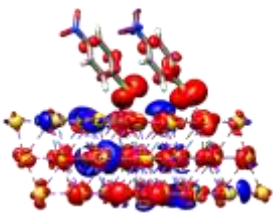<br>State 156<br>Energy: 2.911 eV<br>Osc.: 0.019
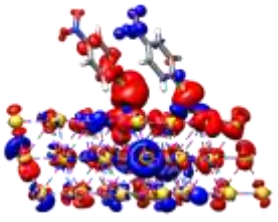<br>State 158<br>Energy: 2.923 eV<br>Osc.: 0.012	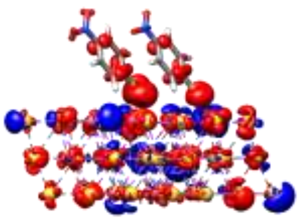<br>State 159<br>Energy: 2.931 eV<br>Osc.: 0.195	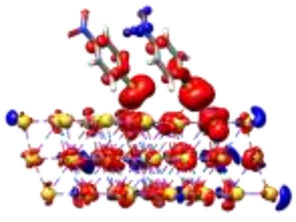<br>State 160<br>Energy: 2.932 eV<br>Osc.: 0.074	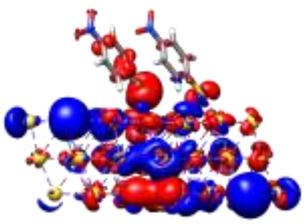<br>State 161<br>Energy: 2.940 eV<br>Osc.: 0.012

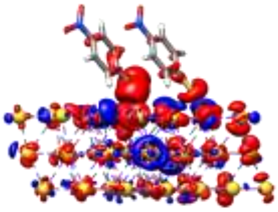<br>State 162<br>Energy: 2.949 eV<br>Osc.: 0.196	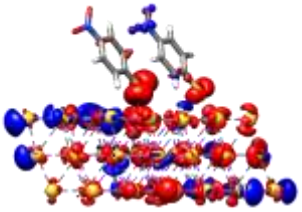<br>State 164<br>Energy: 2.962 eV<br>Osc.: 0.040	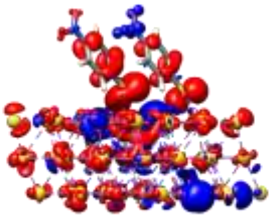<br>State 165<br>Energy: 2.966 eV<br>Osc.: 0.037	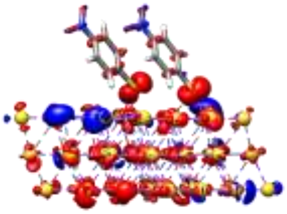<br>State 166<br>Energy: 2.976 eV<br>Osc.: 0.248
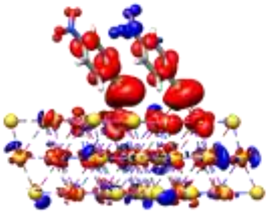<br>State 167<br>Energy: 2.991 eV<br>Osc.: 0.024	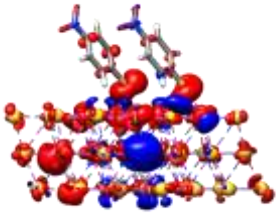<br>State 168<br>Energy: 2.995 eV<br>Osc.: 0.088	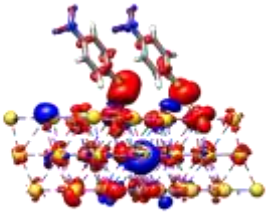<br>State 169<br>Energy: 3.003 eV<br>Osc.: 0.038	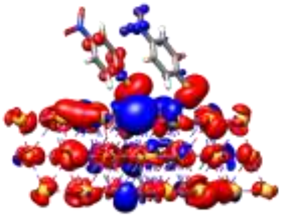<br>State 170<br>Energy: 3.006 eV<br>Osc.: 0.030

Table S3: Charge density differences (CDDs) illustrating the nature of the low-lying bright excitations of product 2 (DMAB). Charge transfer takes place from red to blue.

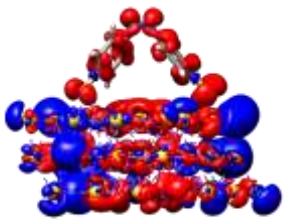<br>State 43<br>Energy: 1.627 eV<br>Osc.: 0.019	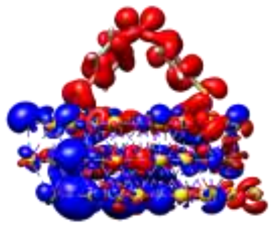<br>State 45<br>Energy: 1.651 eV<br>Osc.: 0.014	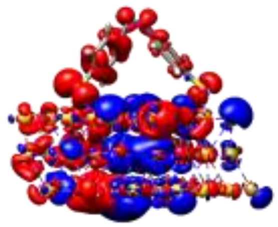<br>State 48<br>Energy: 1.705 eV<br>Osc.: 0.015	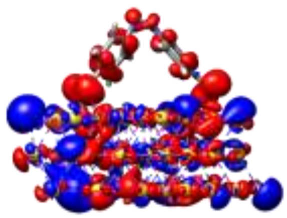<br>State 56<br>Energy: 1.840 eV<br>Osc.: 0.017
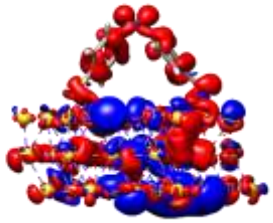<br>State 57<br>Energy: 1.870 eV<br>Osc.: 0.017	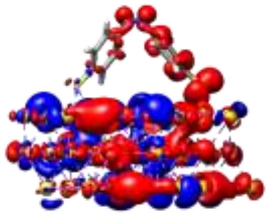<br>State 64<br>Energy: 1.963 eV<br>Osc.: 0.003	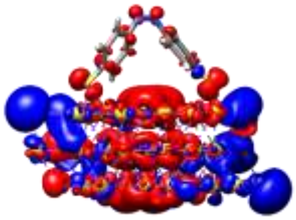<br>State 66<br>Energy: 1.986 eV<br>Osc.: 0.002	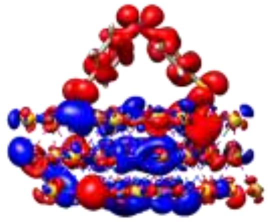<br>State 67<br>Energy: 1.989 eV<br>Osc.: 0.004
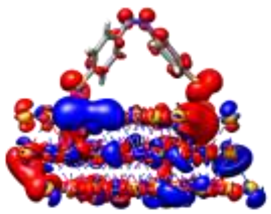<br>State 68<br>Energy: 2.003 eV<br>Osc.: 0.002	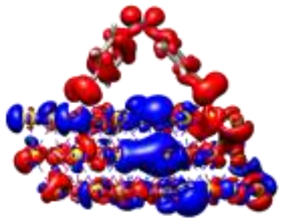<br>State 69<br>Energy: 2.016 eV<br>Osc.: 0.000	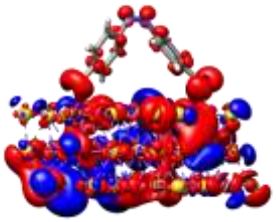<br>State 70<br>Energy: 2.035 eV<br>Osc.: 0.012	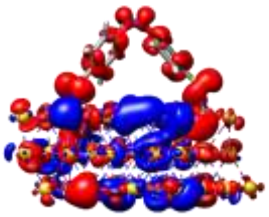<br>State 71<br>Energy: 2.039 eV<br>Osc.: 0.011

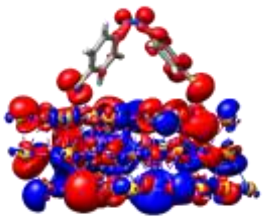<br>State 75<br>Energy: 2.096 eV<br>Osc.: 0.051	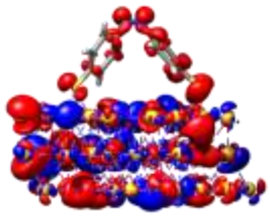<br>State 79<br>Energy: 2.140 eV<br>Osc.: 0.034	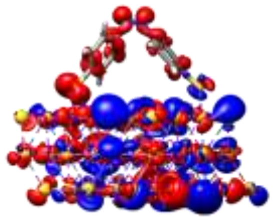<br>State 84<br>Energy: 2.198 eV<br>Osc.: 0.017	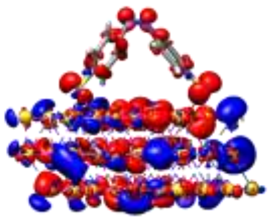<br>State 86<br>Energy: 2.224 eV<br>Osc.: 0.016
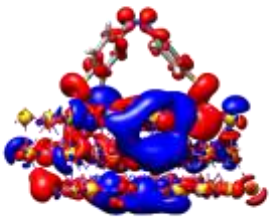<br>State 87<br>Energy: 2.243 eV<br>Osc.: 0.011	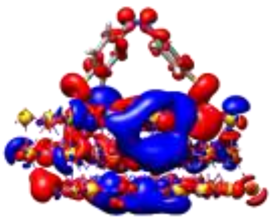<br>State 87<br>Energy: 2.243 eV<br>Osc.: 0.011	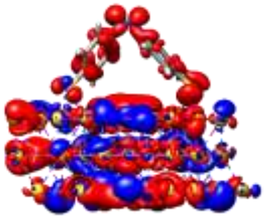<br>State 92<br>Energy: 2.305 eV<br>Osc.: 0.017	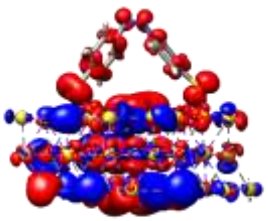<br>State 95<br>Energy: 2.337 eV<br>Osc.: 0.049
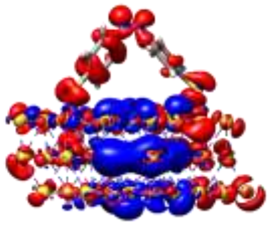<br>State 96<br>Energy: 2.347 eV<br>Osc.: 0.012	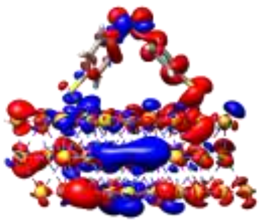<br>State 101<br>Energy: 2.400 eV<br>Osc.: 0.016	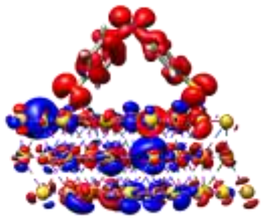<br>State 102<br>Energy: 2.414 eV<br>Osc.: 0.020	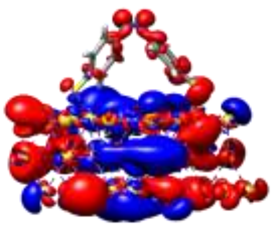<br>State 103<br>Energy: 2.418 eV<br>Osc.: 0.012
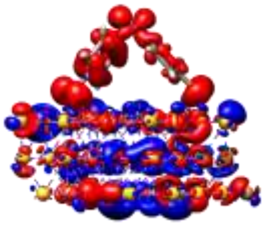<br>State 104<br>Energy: 2.434 eV<br>Osc.: 0.018	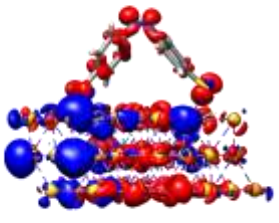<br>State 106<br>Energy: 2.444 eV<br>Osc.: 0.089	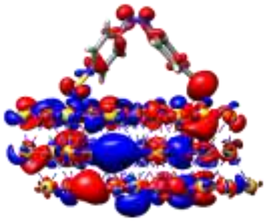<br>State 107<br>Energy: 2.462 eV<br>Osc.: 0.011	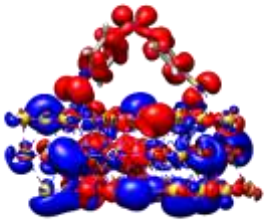<br>State 108<br>Energy: 2.476 eV<br>Osc.: 0.010

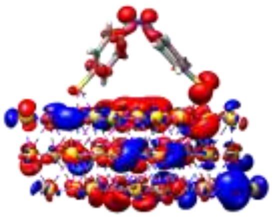

State 109
Energy: 2.480 eV
Osc.: 0.047

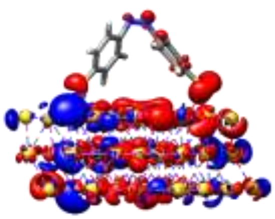

State 110
Energy: 2.493 eV
Osc.: 0.056

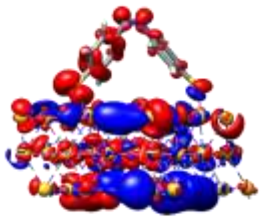

State 111
Energy: 2.511 eV
Osc.: 0.010

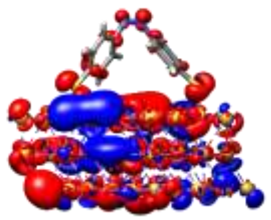

State 112
Energy: 2.517 eV
Osc.: 0.021

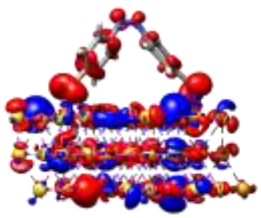

State 115
Energy: 2.553 eV
Osc.: 0.011

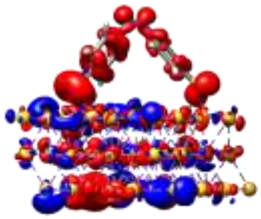

State 116
Energy: 2.561 eV
Osc.: 0.024

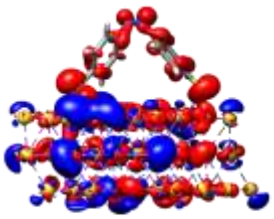

State 117
Energy: 2.575 eV
Osc.: 0.045

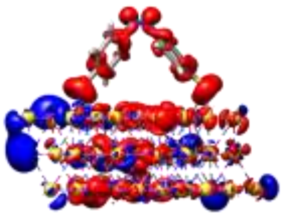

State 118
Energy: 2.584 eV
Osc.: 0.037

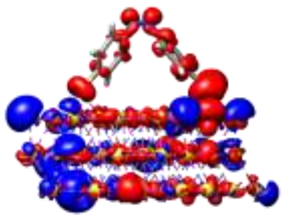

State 120
Energy: 2.609 eV
Osc.: 0.011

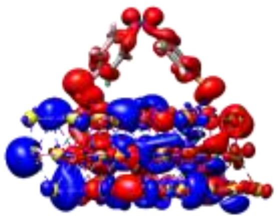

State 123
Energy: 2.635 eV
Osc.: 0.113

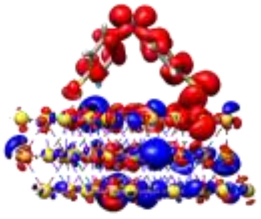

State 124
Energy: 2.641 eV
Osc.: 0.042

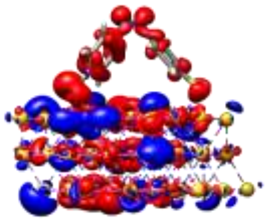

State 125
Energy: 2.646 eV
Osc.: 0.046

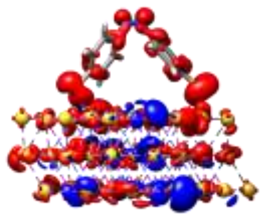

State 126
Energy: 2.658 eV
Osc.: 0.101

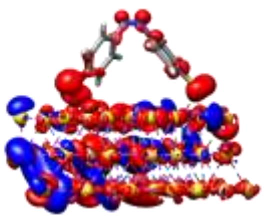

State 127
Energy: 2.663 eV
Osc.: 0.101

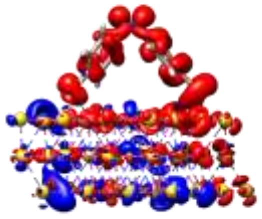

State 129
Energy: 2.683 eV
Osc.: 0.033

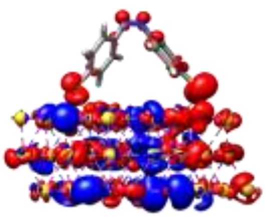

State 130
Energy: 2.694 eV
Osc.: 0.037

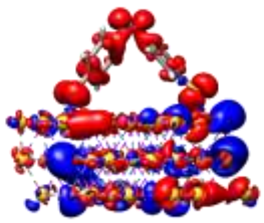	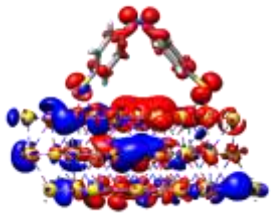	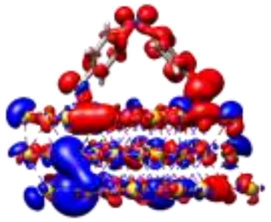	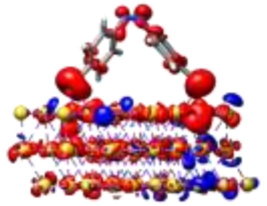
State 133<br>Energy: 2.718 eV<br>Osc.: 0.015	State 134<br>Energy: 2.727 eV<br>Osc.: 0.011	State 136<br>Energy: 2.735 eV<br>Osc.: 0.074	State 137<br>Energy: 2.745 eV<br>Osc.: 0.039
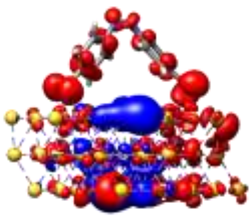	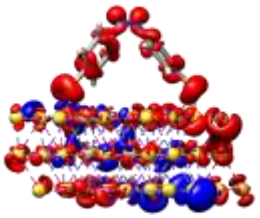	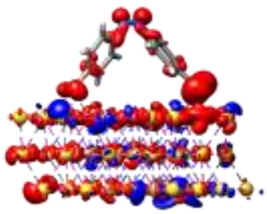	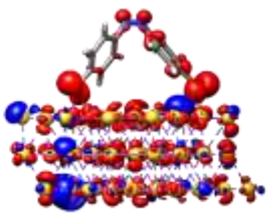
State 139<br>Energy: 2.764 eV<br>Osc.: 0.011	State 140<br>Energy: 2.770 eV<br>Osc.: 0.017	State 141<br>Energy: 2.789 eV<br>Osc.: 0.012	State 142<br>Energy: 2.808 eV<br>Osc.: 0.039
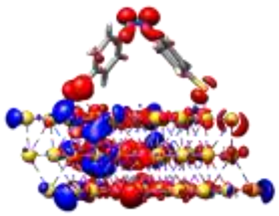	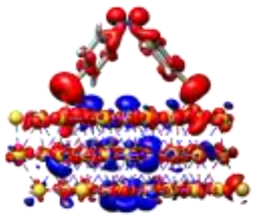	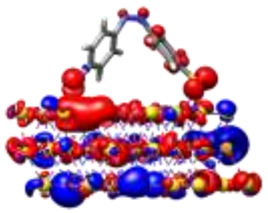	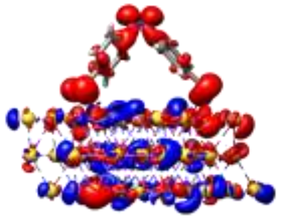
State 143<br>Energy: 2.812 eV<br>Osc.: 0.147	State 144<br>Energy: 2.819 eV<br>Osc.: 0.064	State 145<br>Energy: 2.827 eV<br>Osc.: 0.030	State 146<br>Energy: 2.834 eV<br>Osc.: 0.073
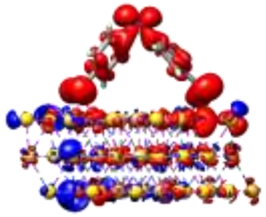	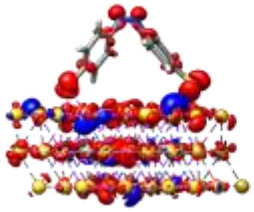	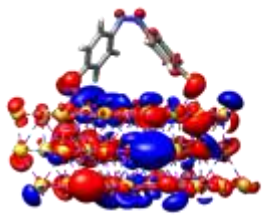	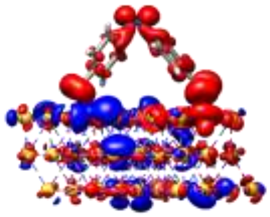
State 147<br>Energy: 2.846 eV<br>Osc.: 0.014	State 151<br>Energy: 2.868 eV<br>Osc.: 0.020	State 152<br>Energy: 2.883 eV<br>Osc.: 0.036	State 154<br>Energy: 2.902 eV<br>Osc.: 0.016

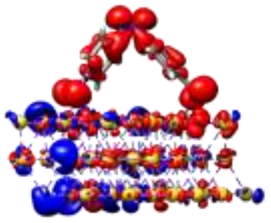<br>State 155<br>Energy: 2.918 eV<br>Osc.: 0.024	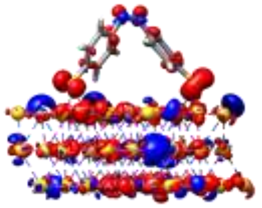<br>State 157<br>Energy: 2.928 eV<br>Osc.: 0.141	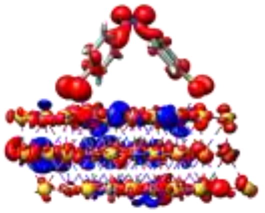<br>State 158<br>Energy: 2.937 eV<br>Osc.: 0.169	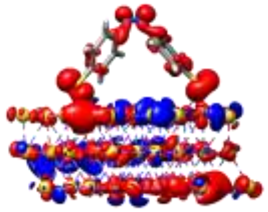<br>State 159<br>Energy: 2.943 eV<br>Osc.: 0.181
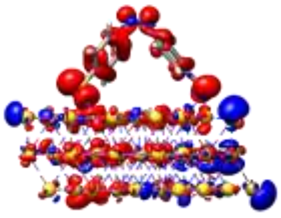<br>State 160<br>Energy: 2.952 eV<br>Osc.: 0.014	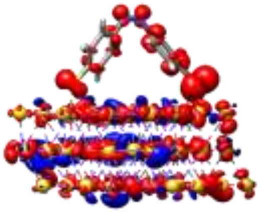<br>State 161<br>Energy: 2.958 eV<br>Osc.: 0.330	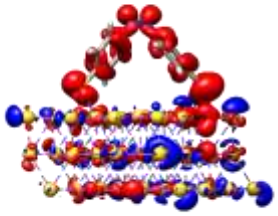<br>State 163<br>Energy: 2.972 eV<br>Osc.: 0.026	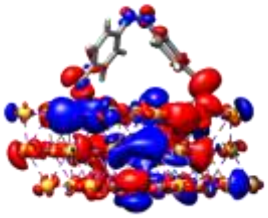<br>State 165<br>Energy: 2.992 eV<br>Osc.: 0.147
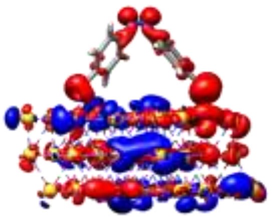<br>State 166<br>Energy: 3.000 eV<br>Osc.: 0.012	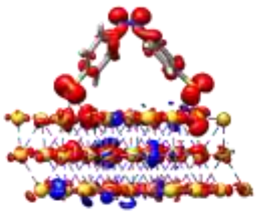<br>State 167<br>Energy: 3.003 eV<br>Osc.: 0.086	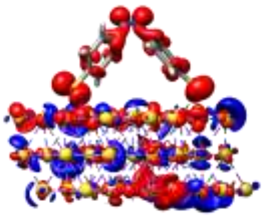<br>State 168<br>Energy: 3.012 eV<br>Osc.: 0.120	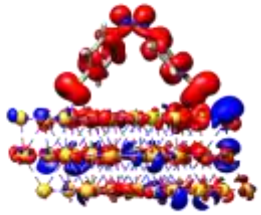<br>State 171<br>Energy: 3.031 eV<br>Osc.: 0.040
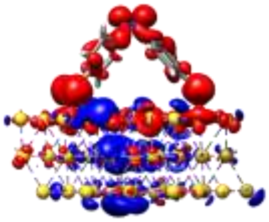<br>State 172<br>Energy: 3.038 eV<br>Osc.: 0.040	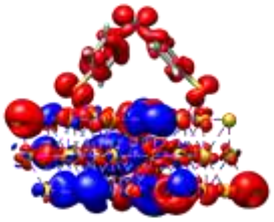<br>State 175<br>Energy: 3.053 eV<br>Osc.: 0.032	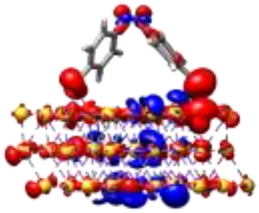<br>State 176<br>Energy: 3.060 eV<br>Osc.: 0.348	

Table S4: Charge density differences (CDDs) illustrating the nature of the low-lying bright excitations of product 3 (DMABBA). Charge transfer takes place from red to blue.

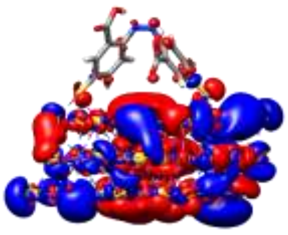 State 43<br>Energy: 1.648 eV<br>Osc.: 0.010	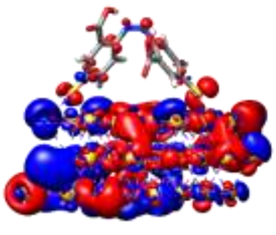 State 46<br>Energy: 1.701 eV<br>Osc.: 0.010	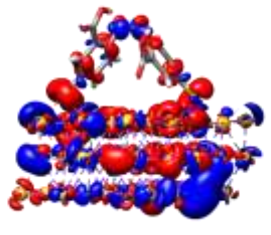 State 47<br>Energy: 1.716 eV<br>Osc.: 0.046	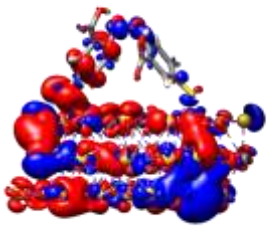 State 52<br>Energy: 1.813 eV<br>Osc.: 0.015
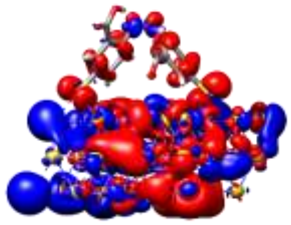 State 55<br>Energy: 1.851 eV<br>Osc.: 0.018	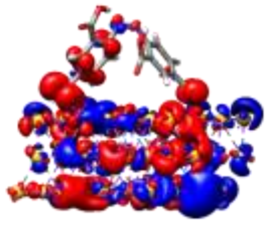 State 56<br>Energy: 1.871 eV<br>Osc.: 0.010	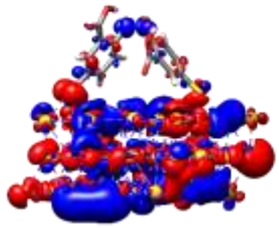 State 57<br>Energy: 1.894 eV<br>Osc.: 0.012	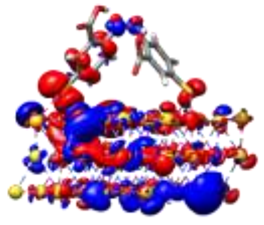 State 69<br>Energy: 2.056 eV<br>Osc.: 0.020
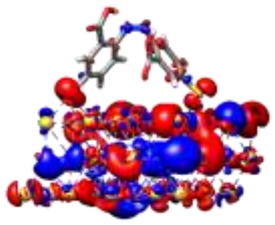 State 72<br>Energy: 2.084 eV<br>Osc.: 0.040	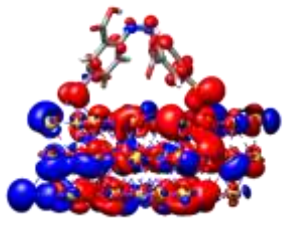 State 75<br>Energy: 2.136 eV<br>Osc.: 0.011	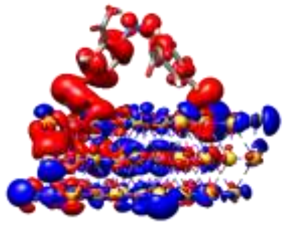 State 76<br>Energy: 2.147 eV<br>Osc.: 0.026	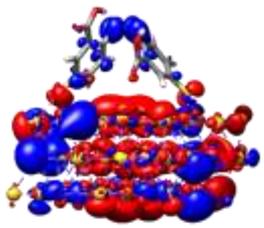 State 77<br>Energy: 2.158 eV<br>Osc.: 0.037

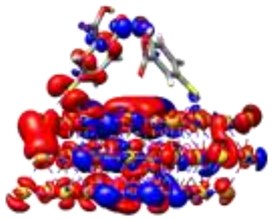

State 81
Energy: 2.200 eV
Osc.: 0.016

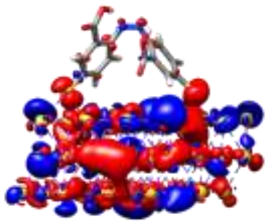

State 85
Energy: 2.241 eV
Osc.: 0.012

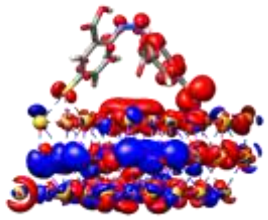

State 91
Energy: 2.319 eV
Osc.: 0.045

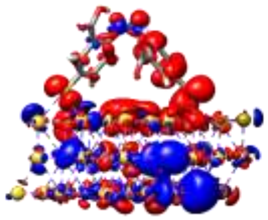

State 93
Energy: 2.339 eV
Osc.: 0.016

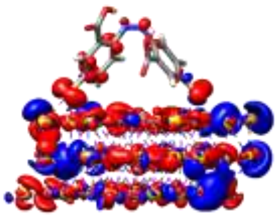

State 99
Energy: 2.404 eV
Osc.: 0.010

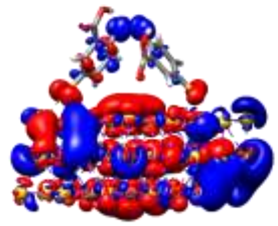

State 101
Energy: 2.429 eV
Osc.: 0.011

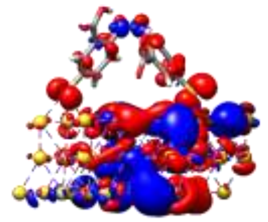

State 103
Energy: 2.449 eV
Osc.: 0.080

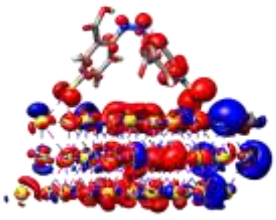

State 104
Energy: 2.460 eV
Osc.: 0.051

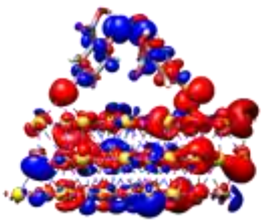

State 105
Energy: 2.471 eV
Osc.: 0.019

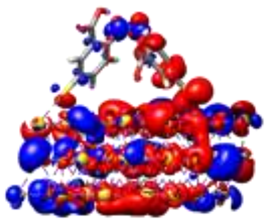

State 106
Energy: 2.477 eV
Osc.: 0.010

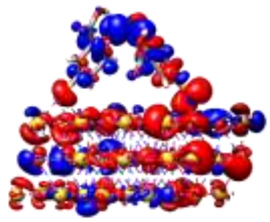

State 107
Energy: 2.481 eV
Osc.: 0.028

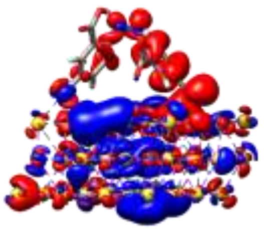

State 110
Energy: 2.528 eV
Osc.: 0.036

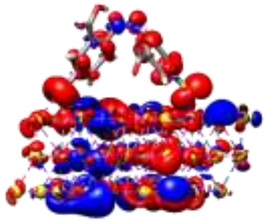

State 112
Energy: 2.541 eV
Osc.: 0.014

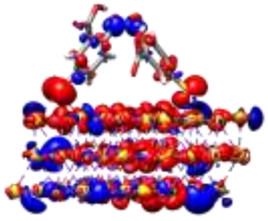

State 113
Energy: 2.554 eV
Osc.: 0.069

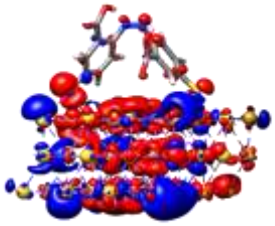

State 116
Energy: 2.584 eV
Osc.: 0.033

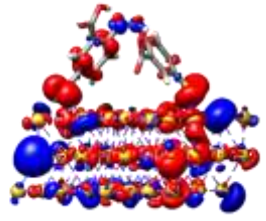

State 118
Energy: 2.597 eV
Osc.: 0.066

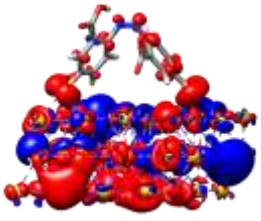

State 122
Energy: 2.635 eV
Osc.: 0.051

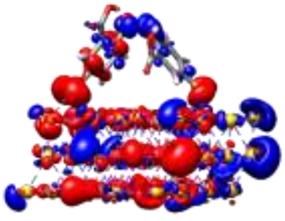

State 123
Energy: 2.646 eV
Osc.: 0.014

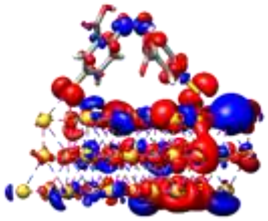

State 124
Energy: 2.656 eV
Osc.: 0.157

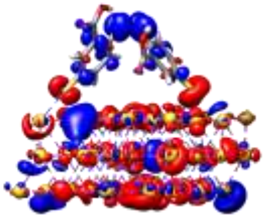

State 126
Energy: 2.674 eV
Osc.: 0.176

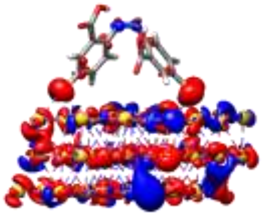

State 128
Energy: 2.687 eV
Osc.: 0.025

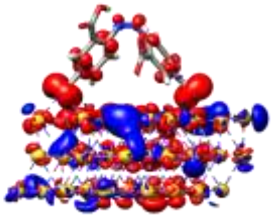

State 129
Energy: 2.701 eV
Osc.: 0.061

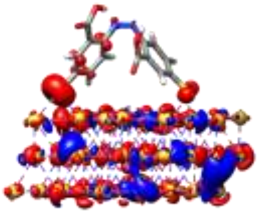

State 130
Energy: 2.708 eV
Osc.: 0.013

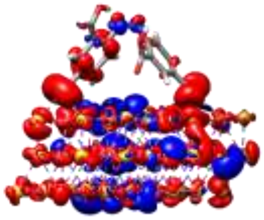

State 131
Energy: 2.714 eV
Osc.: 0.010

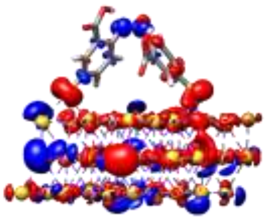<br>State 132<br>Energy: 2.722 eV<br>Osc.: 0.067	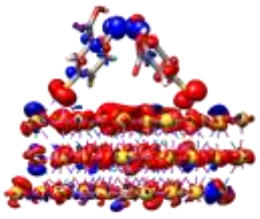<br>State 133<br>Energy: 2.739 eV<br>Osc.: 0.016	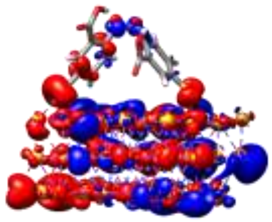<br>State 135<br>Energy: 2.749 eV<br>Osc.: 0.010	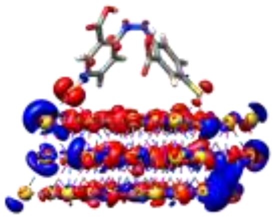<br>State 136<br>Energy: 2.760 eV<br>Osc.: 0.011
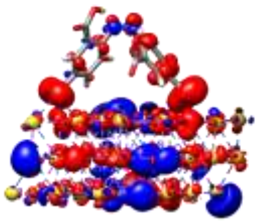<br>State 137<br>Energy: 2.769 eV<br>Osc.: 0.126	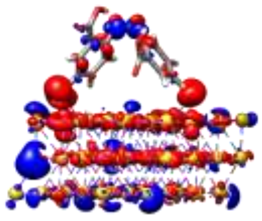<br>State 138<br>Energy: 2.778 eV<br>Osc.: 0.066	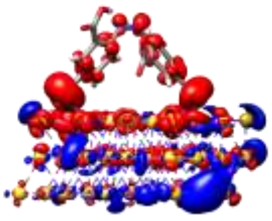<br>State 139<br>Energy: 2.788 eV<br>Osc.: 0.033	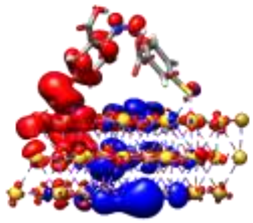<br>State 141<br>Energy: 2.812 eV<br>Osc.: 0.020
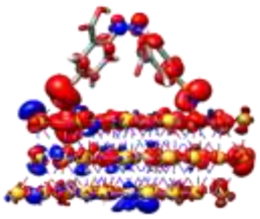<br>State 143<br>Energy: 2.826 eV<br>Osc.: 0.016	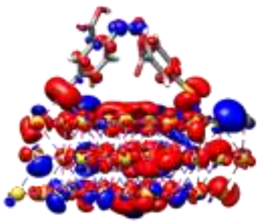<br>State 144<br>Energy: 2.842 eV<br>Osc.: 0.015	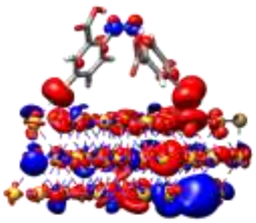<br>State 145<br>Energy: 2.851 eV<br>Osc.: 0.028	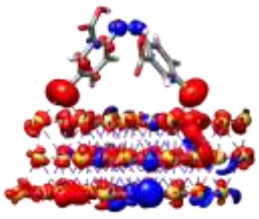<br>State 146<br>Energy: 2.860 eV<br>Osc.: 0.041

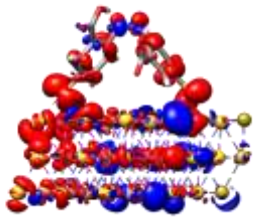<br>State 148<br>Energy: 2.866 eV<br>Osc.: 0.012	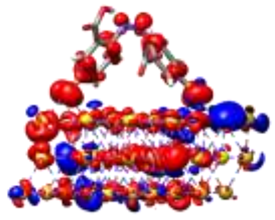<br>State 150<br>Energy: 2.889 eV<br>Osc.: 0.030	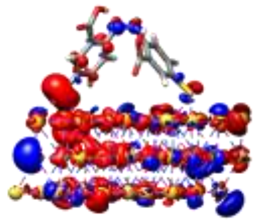<br>State 151<br>Energy: 2.900 eV<br>Osc.: 0.025	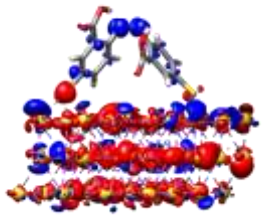<br>State 152<br>Energy: 2.910 eV<br>Osc.: 0.055
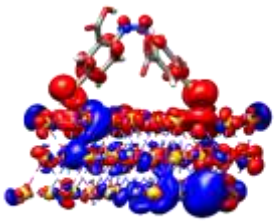<br>State 153<br>Energy: 2.912 eV<br>Osc.: 0.042	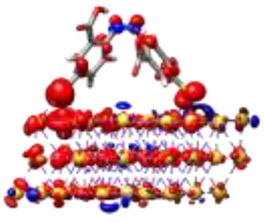<br>State 154<br>Energy: 2.916 eV<br>Osc.: 0.179	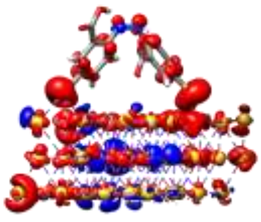<br>State 155<br>Energy: 2.929 eV<br>Osc.: 0.145	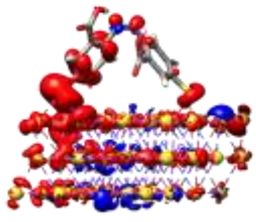<br>State 156<br>Energy: 2.935 eV<br>Osc.: 0.109
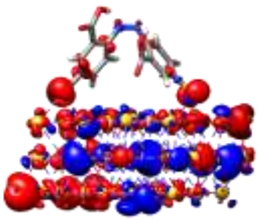<br>State 158<br>Energy: 2.953 eV<br>Osc.: 0.050	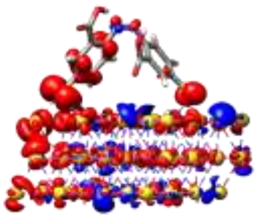<br>State 159<br>Energy: 2.957 eV<br>Osc.: 0.057	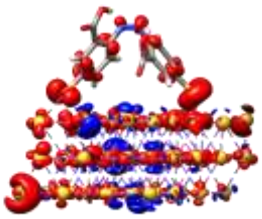<br>State 160<br>Energy: 2.969 eV<br>Osc.: 0.301	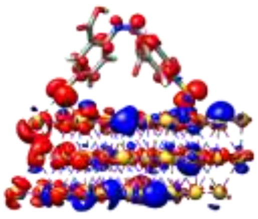<br>State 161<br>Energy: 2.979 eV<br>Osc.: 0.040

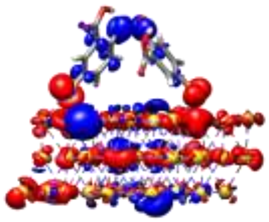 State 164 Energy: 2.995 eV Osc.: 0.102	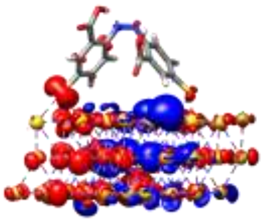 State 165 Energy: 3.002 eV Osc.: 0.030		

Table S5: Charge density differences (CDDs) illustrating the nature of the low-lying bright excitations of product (4). Charge transfer takes place from red to blue.

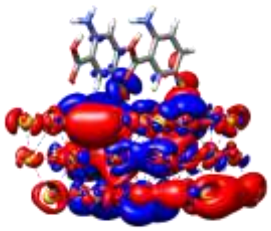<br>State 45<br>Energy: 1.538 eV<br>Osc.: 0.011	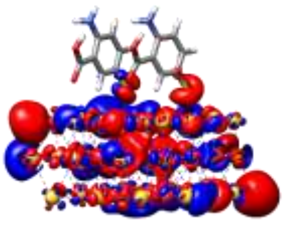<br>State 48<br>Energy: 1.602 eV<br>Osc.: 0.043	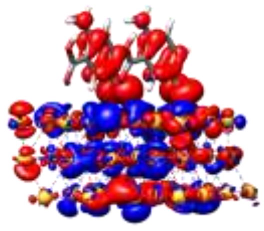<br>State 55<br>Energy: 1.727 eV<br>Osc.: 0.017	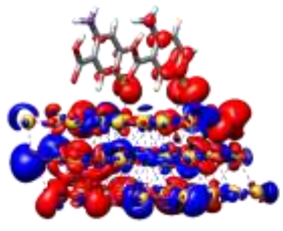<br>State 57<br>Energy: 1.758 eV<br>Osc.: 0.015
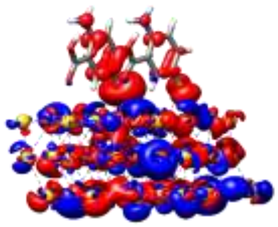<br>State 58<br>Energy: 1.781 eV<br>Osc.: 0.009	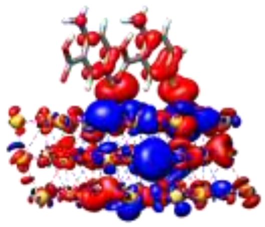<br>State 60<br>Energy: 1.808 eV<br>Osc.: 0.012	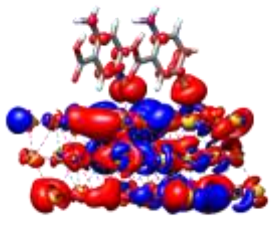<br>State 64<br>Energy: 1.859 eV<br>Osc.: 0.024	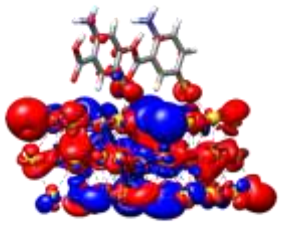<br>State 71<br>Energy: 1.960 eV<br>Osc.: 0.021
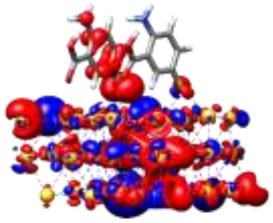<br>State 73<br>Energy: 1.991 eV<br>Osc.: 0.012	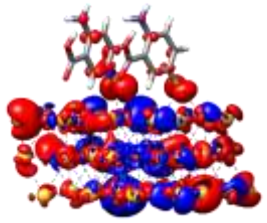<br>State 80<br>Energy: 2.078 eV<br>Osc.: 0.013	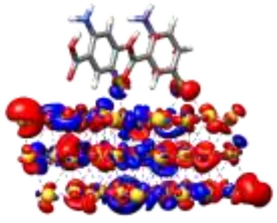<br>State 81<br>Energy: 2.107 eV<br>Osc.: 0.082	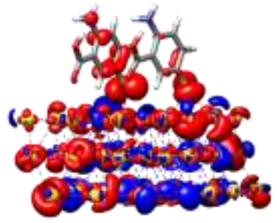<br>State 83<br>Energy: 2.127 eV<br>Osc.: 0.015

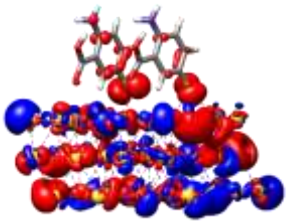<br>State 86<br>Energy: 2.159 eV<br>Osc.: 0.013	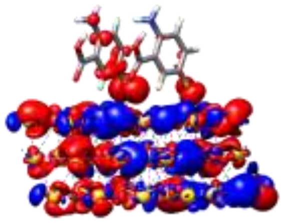<br>State 87<br>Energy: 2.169 eV<br>Osc.: 0.015	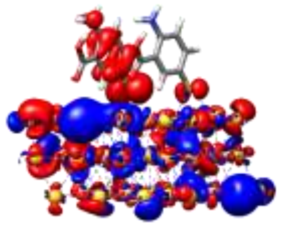<br>State 88<br>Energy: 2.186 eV<br>Osc.: 0.020	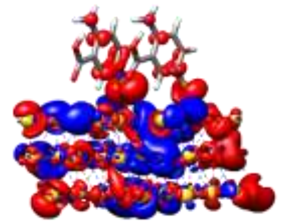<br>State 90<br>Energy: 2.232 eV<br>Osc.: 0.009
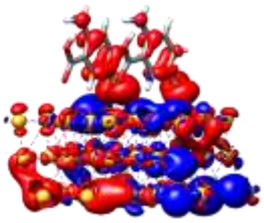<br>State 92<br>Energy: 2.245 eV<br>Osc.: 0.059	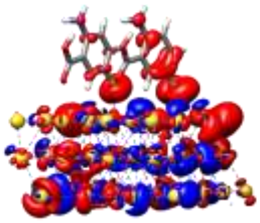<br>State 94<br>Energy: 2.274 eV<br>Osc.: 0.032	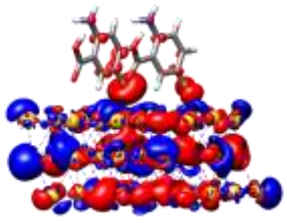<br>State 98<br>Energy: 2.326 eV<br>Osc.: 0.036	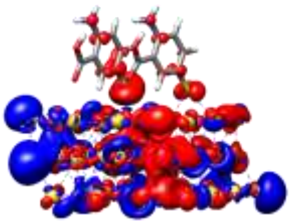<br>State 101<br>Energy: 2.369 eV<br>Osc.: 0.012
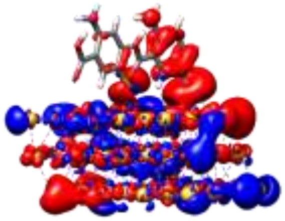<br>State 112<br>Energy: 2.500 eV<br>Osc.: 0.011	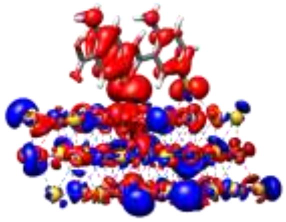<br>State 113<br>Energy: 2.511 eV<br>Osc.: 0.035	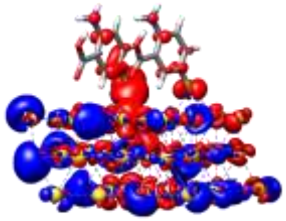<br>State 114<br>Energy: 2.516 eV<br>Osc.: 0.017	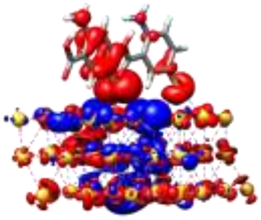<br>State 119<br>Energy: 2.560 eV<br>Osc.: 0.169

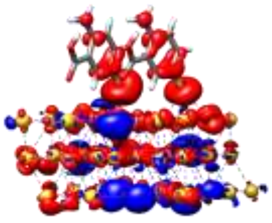	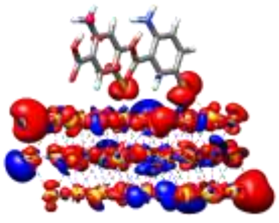	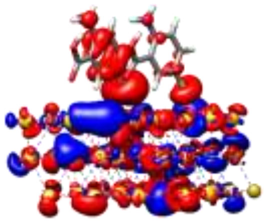	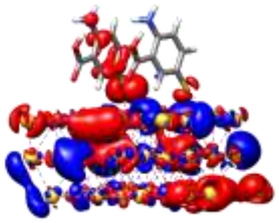
State 123<br>Energy: 2.597 eV<br>Osc.: 0.047	State 124<br>Energy: 2.607 eV<br>Osc.: 0.012	State 125<br>Energy: 2.626 eV<br>Osc.: 0.048	State 126<br>Energy: 2.627 eV<br>Osc.: 0.014
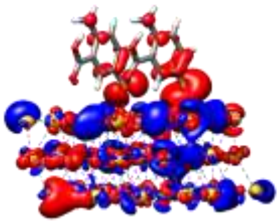	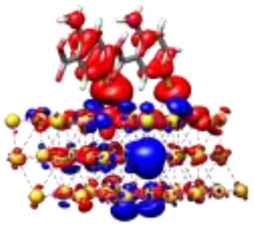	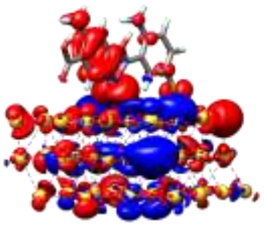	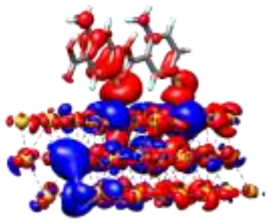
State 129<br>Energy: 2.653 eV<br>Osc.: 0.013	State 130<br>Energy: 2.661 eV<br>Osc.: 0.038	State 131<br>Energy: 2.673 eV<br>Osc.: 0.012	State 132<br>Energy: 2.689 eV<br>Osc.: 0.012
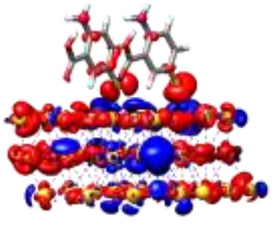	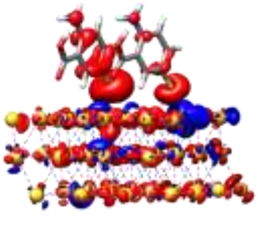	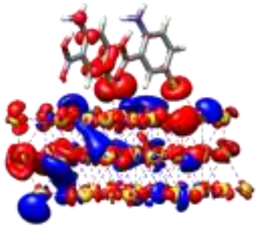	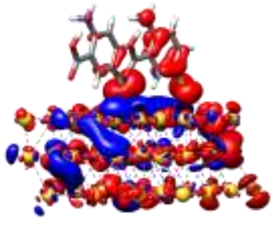
State 134<br>Energy: 2.701 eV<br>Osc.: 0.011	State 135<br>Energy: 2.708 eV<br>Osc.: 0.026	State 137<br>Energy: 2.730 eV<br>Osc.: 0.009	State 138<br>Energy: 2.743 eV<br>Osc.: 0.040

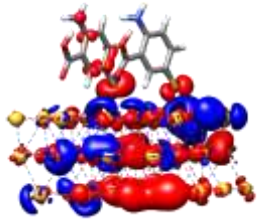

State 139
Energy: 2.746 eV
Osc.: 0.037

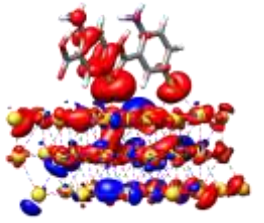

State 140
Energy: 2.751 eV
Osc.: 0.033

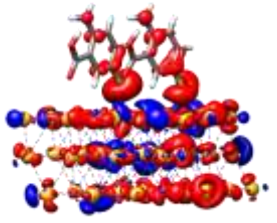

State 141
Energy: 2.763 eV
Osc.: 0.016

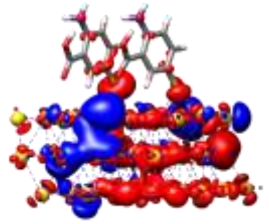

State 142
Energy: 2.771 eV
Osc.: 0.101

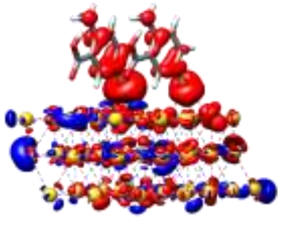

State 143
Energy: 2.785 eV
Osc.: 0.049

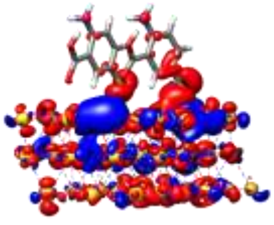

State 144
Energy: 2.791 eV
Osc.: 0.043

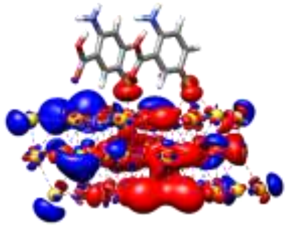

State 145
Energy: 2.806 eV
Osc.: 0.255

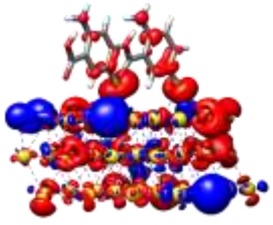

State 146
Energy: 2.811 eV
Osc.: 0.112

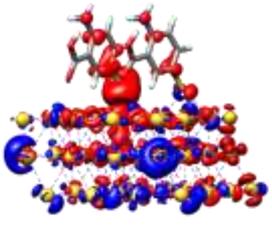

State 148
Energy: 2.832 eV
Osc.: 0.072

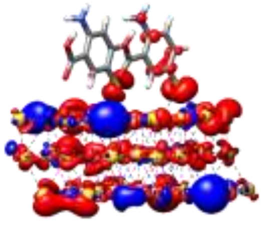

State 149
Energy: 2.832 eV
Osc.: 0.050

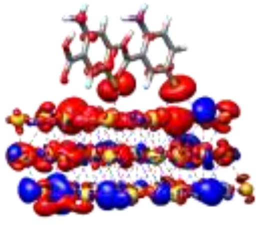

State 150
Energy: 2.837 eV
Osc.: 0.015

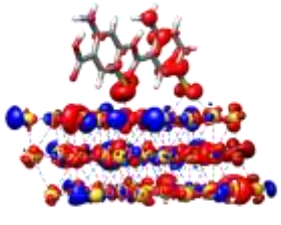

State 151
Energy: 2.845 eV
Osc.: 0.026

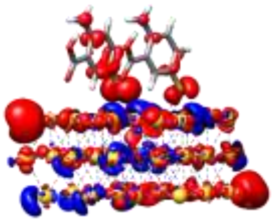<br>State 153<br>Energy: 2.871 eV<br>Osc.: 0.117	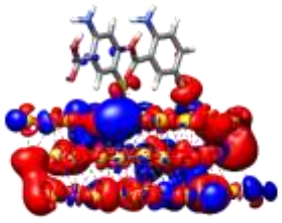<br>State 154<br>Energy: 2.879 eV<br>Osc.: 0.015	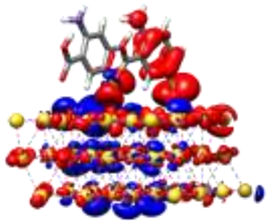<br>State 155<br>Energy: 2.886 eV<br>Osc.: 0.084	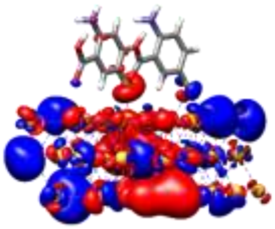<br>State 156<br>Energy: 2.895 eV<br>Osc.: 0.028
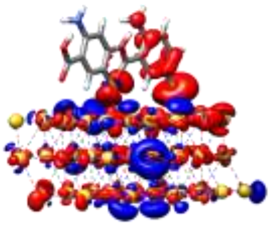<br>State 157<br>Energy: 2.902 eV<br>Osc.: 0.166	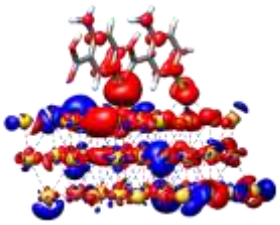<br>State 158<br>Energy: 2.908 eV<br>Osc.: 0.022	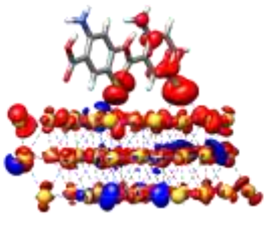<br>State 159<br>Energy: 2.915 eV<br>Osc.: 0.170	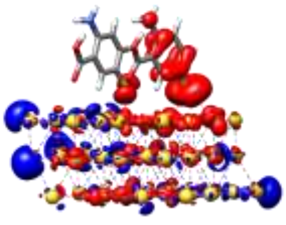<br>State 160<br>Energy: 2.932 eV<br>Osc.: 0.114
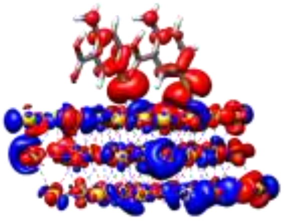<br>State 161<br>Energy: 2.935 eV<br>Osc.: 0.069	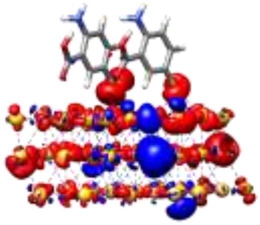<br>State 163<br>Energy: 2.952 eV<br>Osc.: 0.284	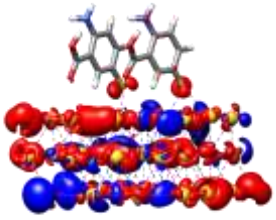<br>State 164<br>Energy: 2.956 eV<br>Osc.: 0.276	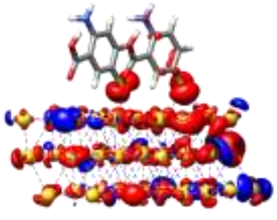<br>State 166<br>Energy: 2.971 eV<br>Osc.: 0.056

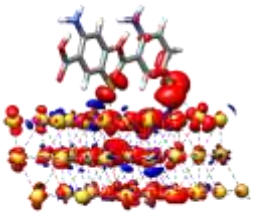<br>State 167<br>Energy: 2.986 eV<br>Osc.: 0.089	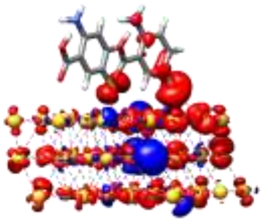<br>State 168<br>Energy: 2.998 eV<br>Osc.: 0.038	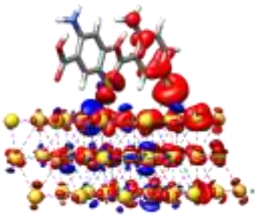<br>State 169<br>Energy: 2.999 eV<br>Osc.: 0.091	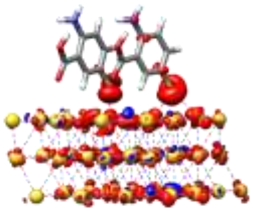<br>State 170<br>Energy: 3.012 eV<br>Osc.: 0.080

Table S6: Charge density differences (CDDs) illustrating the nature of the low-lying bright excitations of intermediate (a). Charge transfer takes place from red to blue.

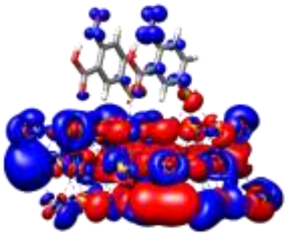<br>State 43<br>Energy: 1.488 eV<br>Osc.: 0.015	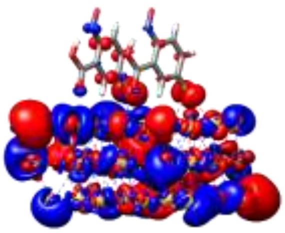<br>State 50<br>Energy: 1.614 eV<br>Osc.: 0.024	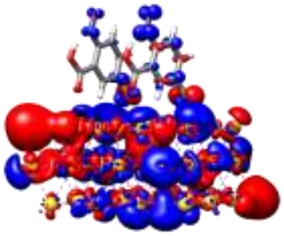<br>State 51<br>Energy: 1.626 eV<br>Osc.: 0.011	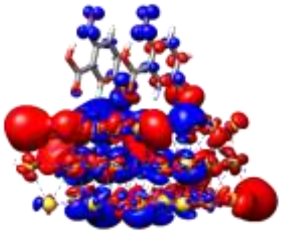<br>State 52<br>Energy: 1.658 eV<br>Osc.: 0.018
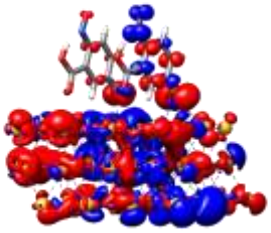<br>State 53<br>Energy: 1.678 eV<br>Osc.: 0.010	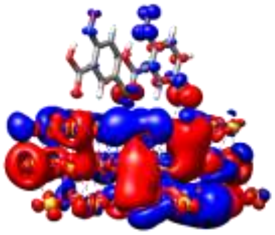<br>State 54<br>Energy: 1.683 eV<br>Osc.: 0.020	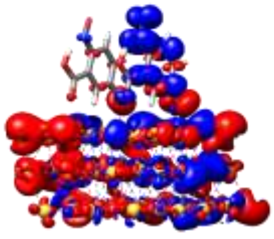<br>State 58<br>Energy: 1.775 eV<br>Osc.: 0.016	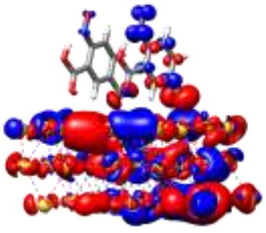<br>State 64<br>Energy: 1.857 eV<br>Osc.: 0.012
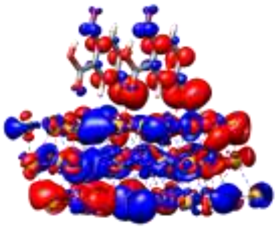<br>State 65<br>Energy: 1.867 eV<br>Osc.: 0.015	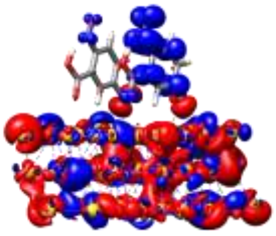<br>State 68<br>Energy: 1.904 eV<br>Osc.: 0.010	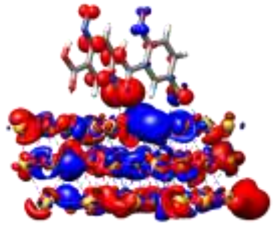<br>State 76<br>Energy: 2.006 eV<br>Osc.: 0.010	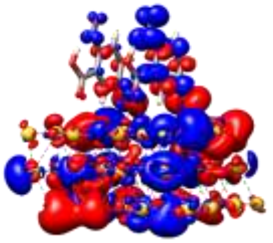<br>State 77<br>Energy: 2.023 eV<br>Osc.: 0.011

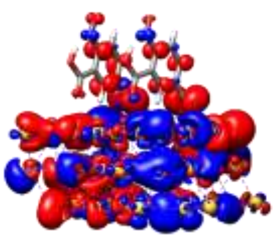

State 78
Energy: 2.037 eV
Osc.: 0.014

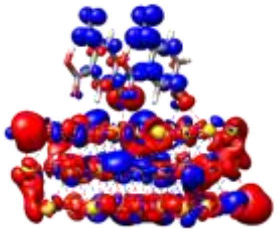

State 85
Energy: 2.124 eV
Osc.: 0.012

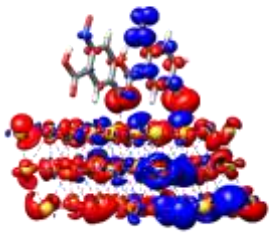

State 86
Energy: 2.133 eV
Osc.: 0.012

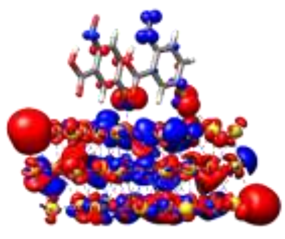

State 87
Energy: 2.149 eV
Osc.: 0.022

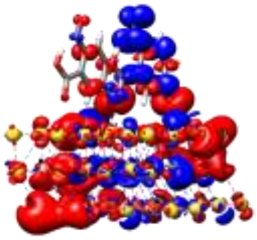

State 88
Energy: 2.161 eV
Osc.: 0.044

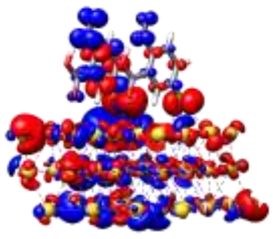

State 89
Energy: 2.173 eV
Osc.: 0.014

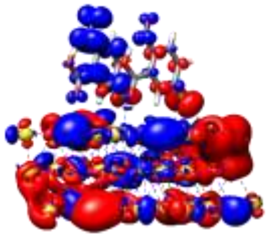

State 91
Energy: 2.195 eV
Osc.: 0.023

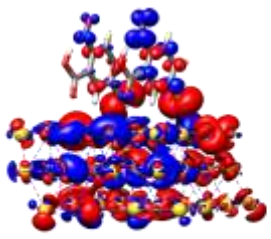

State 97
Energy: 2.259 eV
Osc.: 0.023

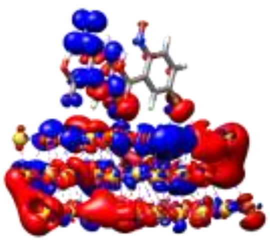

State 98
Energy: 2.288 eV
Osc.: 0.017

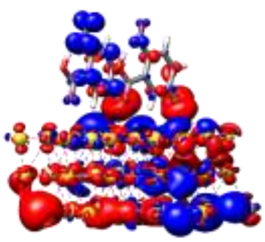

State 99
Energy: 2.294 eV
Osc.: 0.037

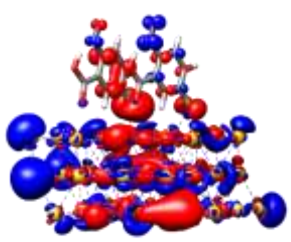

State 104
Energy: 2.365 eV
Osc.: 0.015

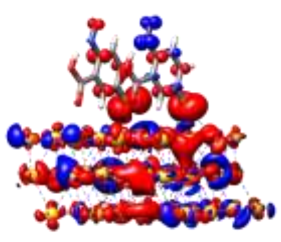

State 106
Energy: 2.382 eV
Osc.: 0.047

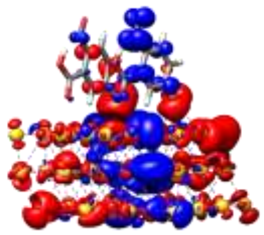<br>State 108<br>Energy: 2.397 eV<br>Osc.: 0.026	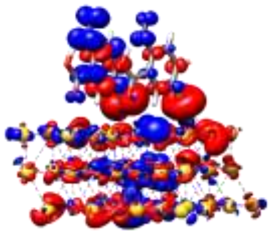<br>State 109<br>Energy: 2.408 eV<br>Osc.: 0.012	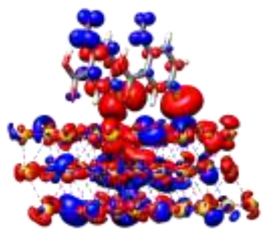<br>State 112<br>Energy: 2.444 eV<br>Osc.: 0.024	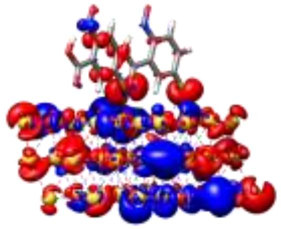<br>State 115<br>Energy: 2.473 eV<br>Osc.: 0.046
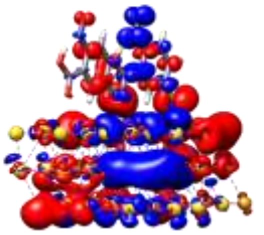<br>State 117<br>Energy: 2.495 eV<br>Osc.: 0.027	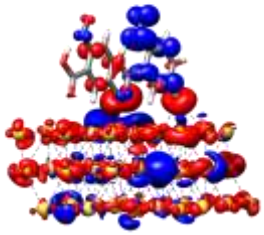<br>State 121<br>Energy: 2.544 eV<br>Osc.: 0.091	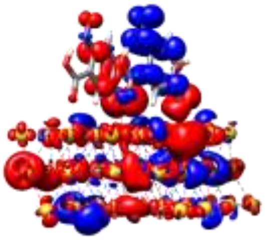<br>State 123<br>Energy: 2.558 eV<br>Osc.: 0.019	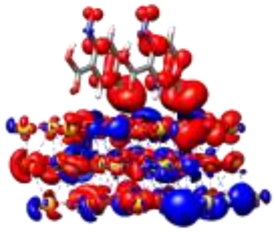<br>State 125<br>Energy: 2.576 eV<br>Osc.: 0.024
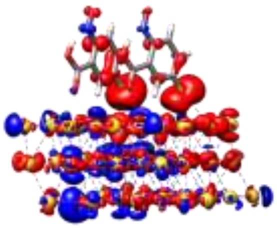<br>State 126<br>Energy: 2.593 eV<br>Osc.: 0.023	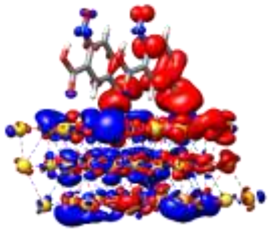<br>State 128<br>Energy: 2.602 eV<br>Osc.: 0.060	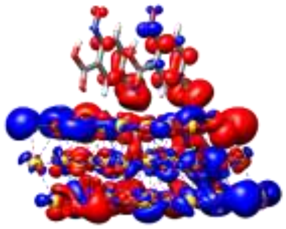<br>State 130<br>Energy: 2.614 eV<br>Osc.: 0.079	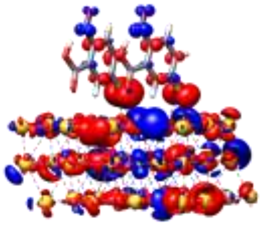<br>State 132<br>Energy: 2.638 eV<br>Osc.: 0.063

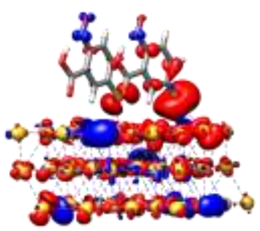

State 133
Energy: 2.639 eV
Osc.: 0.011

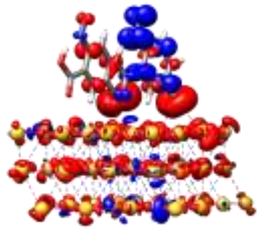

State 134
Energy: 2.657 eV
Osc.: 0.012

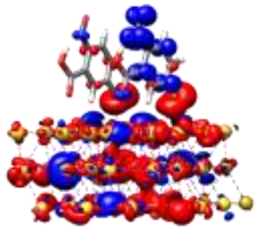

State 135
Energy: 2.668 eV
Osc.: 0.019

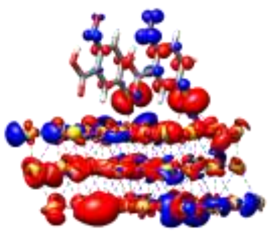

State 136
Energy: 2.670 eV
Osc.: 0.057

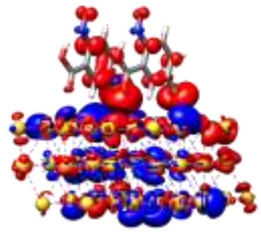

State 138
Energy: 2.685 eV
Osc.: 0.026

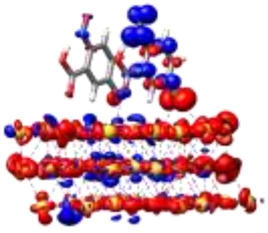

State 139
Energy: 2.693 eV
Osc.: 0.043

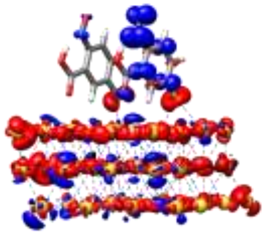

State 141
Energy: 2.711 eV
Osc.: 0.034

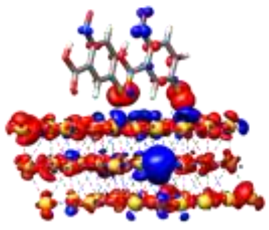

State 142
Energy: 2.726 eV
Osc.: 0.015

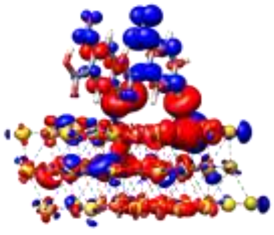

State 144
Energy: 2.738 eV
Osc.: 0.037

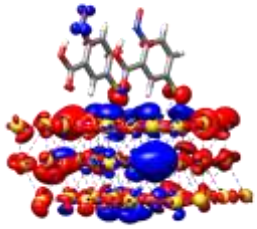

State 145
Energy: 2.744 eV
Osc.: 0.097

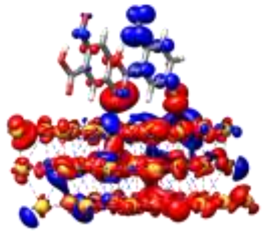

State 146
Energy: 2.759 eV
Osc.: 0.024

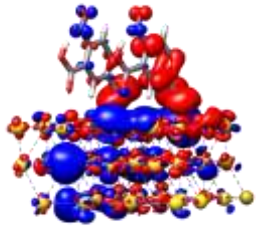

State 148
Energy: 2.774 eV
Osc.: 0.027

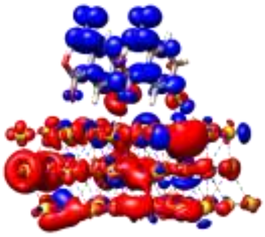<br>State 149<br>Energy: 2.778 eV<br>Osc.: 0.013	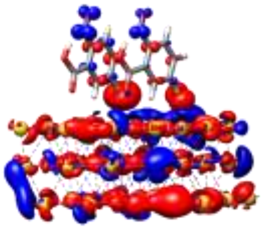<br>State 150<br>Energy: 2.795 eV<br>Osc.: 0.026	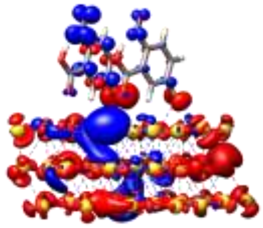<br>State 151<br>Energy: 2.801 eV<br>Osc.: 0.017	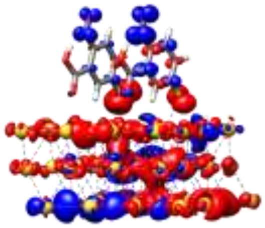<br>State 152<br>Energy: 2.803 eV<br>Osc.: 0.040
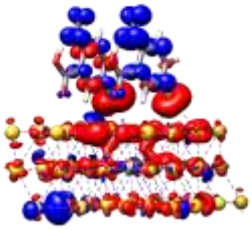<br>State 153<br>Energy: 2.810 eV<br>Osc.: 0.028	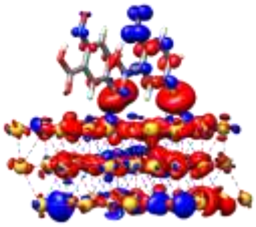<br>State 154<br>Energy: 2.815 eV<br>Osc.: 0.119	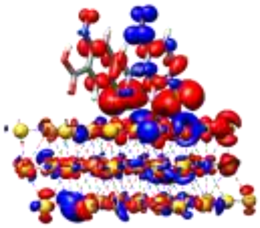<br>State 155<br>Energy: 2.820 eV<br>Osc.: 0.012	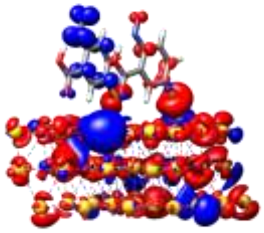<br>State 156<br>Energy: 2.821 eV<br>Osc.: 0.146
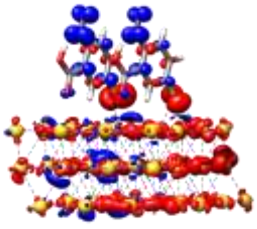<br>State 157<br>Energy: 2.834 eV<br>Osc.: 0.013	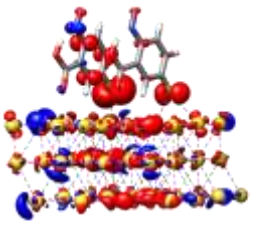<br>State 159<br>Energy: 2.847 eV<br>Osc.: 0.357	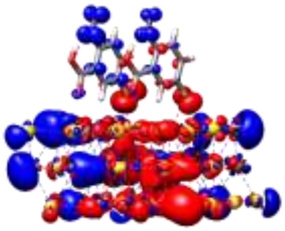<br>State 160<br>Energy: 2.862 eV<br>Osc.: 0.016	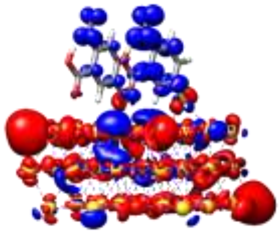<br>State 161<br>Energy: 2.872 eV<br>Osc.: 0.033

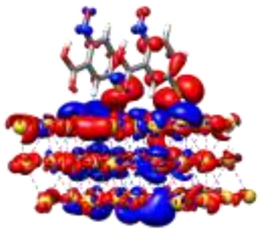 State 164<br>Energy: 2.883 eV<br>Osc.: 0.015	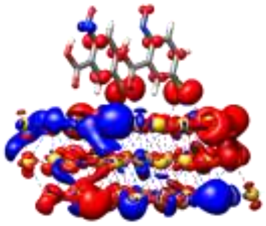 State 165<br>Energy: 2.894 eV<br>Osc.: 0.039	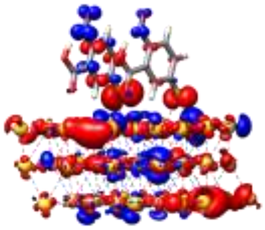 State 166<br>Energy: 2.904 eV<br>Osc.: 0.047	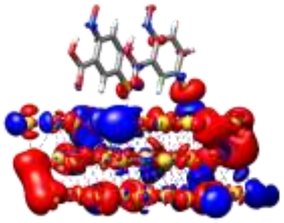 State 167<br>Energy: 2.906 eV<br>Osc.: 0.072
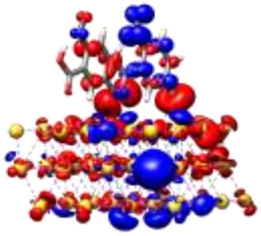 State 168<br>Energy: 2.917 eV<br>Osc.: 0.052	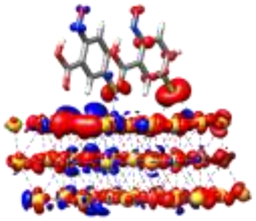 State 169<br>Energy: 2.934 eV<br>Osc.: 0.305	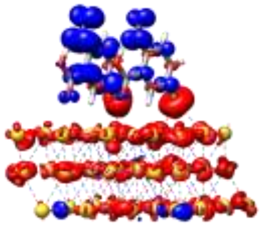 State 170<br>Energy: 2.942 eV<br>Osc.: 0.017	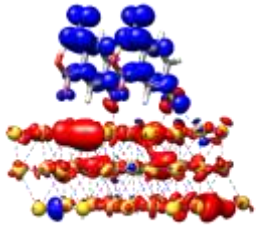 State 171<br>Energy: 2.945 eV<br>Osc.: 0.110
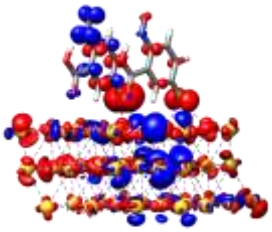 State 173<br>Energy: 2.960 eV<br>Osc.: 0.030	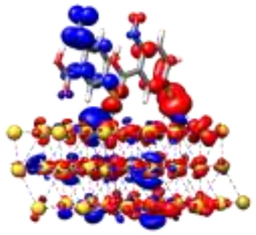 State 174<br>Energy: 2.972 eV<br>Osc.: 0.181	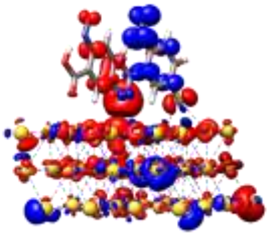 State 175<br>Energy: 2.974 eV<br>Osc.: 0.057	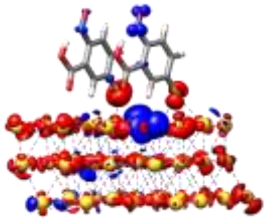 State 176<br>Energy: 2.984 eV<br>Osc.: 0.142

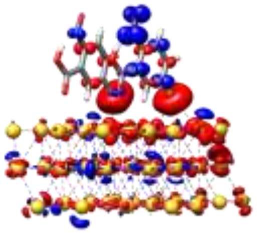<br>State 177<br>Energy: 2.988 eV<br>Osc.: 0.105	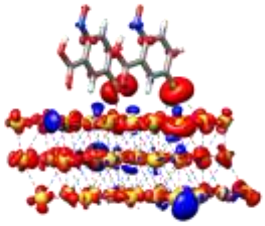<br>State 178<br>Energy: 2.997 eV<br>Osc.: 0.107	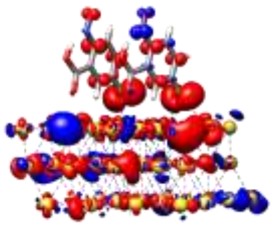<br>State 179<br>Energy: 3.001 eV<br>Osc.: 0.018	

Table S7: Charge density differences (CDDs) illustrating the nature of the low-lying bright excitations of intermediate (b). Charge transfer takes place from red to blue

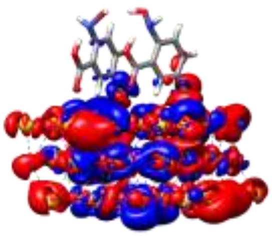<br>State 45<br>Energy: 1.538 eV<br>Osc.: 0.011	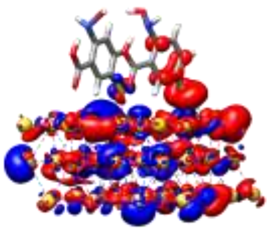<br>State 48<br>Energy: 1.602 eV<br>Osc.: 0.031	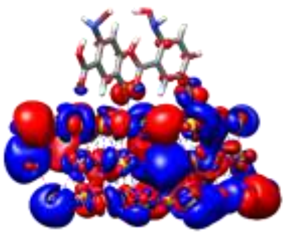<br>State 49<br>Energy: 1.620 eV<br>Osc.: 0.015	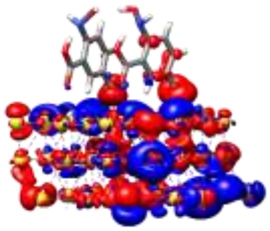<br>State 56<br>Energy: 1.758 eV<br>Osc.: 0.020
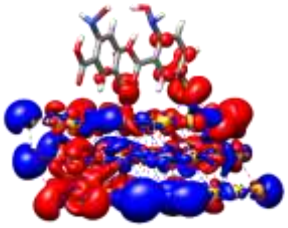<br>State 57<br>Energy: 1.760 eV<br>Osc.: 0.013	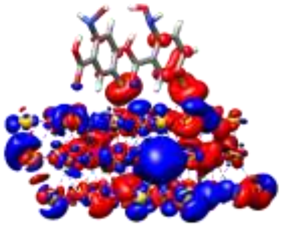<br>State 60<br>Energy: 1.805 eV<br>Osc.: 0.011	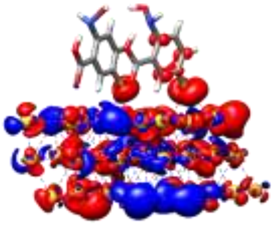<br>State 62<br>Energy: 1.840 eV<br>Osc.: 0.013	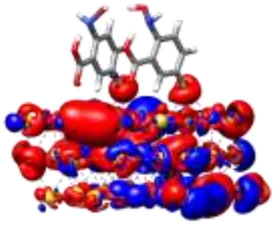<br>State 64<br>Energy: 1.862 eV<br>Osc.: 0.012
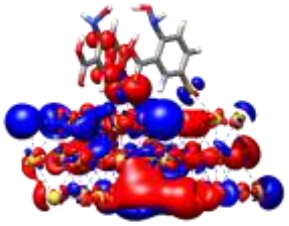<br>State 70<br>Energy: 1.951 eV<br>Osc.: 0.014	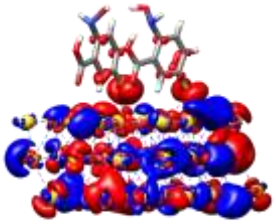<br>State 73<br>Energy: 1.997 eV<br>Osc.: 0.016	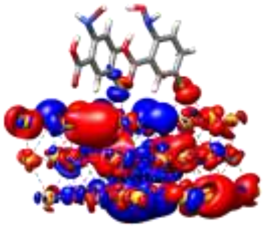<br>State 81<br>Energy: 2.110 eV<br>Osc.: 0.039	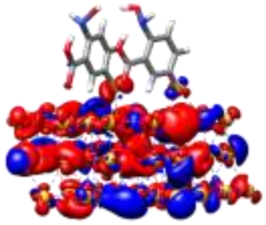<br>State 82<br>Energy: 2.117 eV<br>Osc.: 0.010

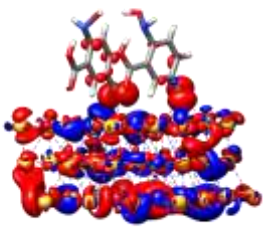

State 83
Energy: 2.124 eV
Osc.: 0.057

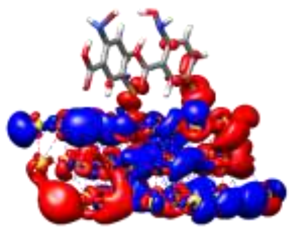

State 86
Energy: 2.168 eV
Osc.: 0.021

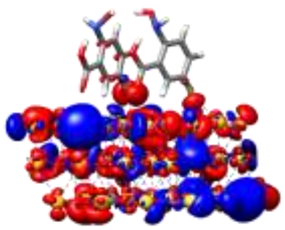

State 88
Energy: 2.209 eV
Osc.: 0.013

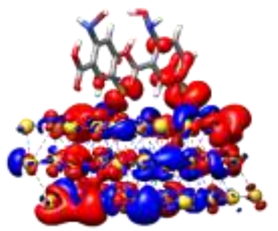

State 94
Energy: 2.286 eV
Osc.: 0.056

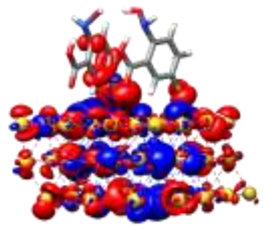

State 95
Energy: 2.293 eV
Osc.: 0.024

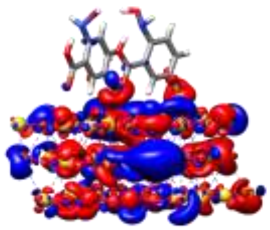

State 96
Energy: 2.321 eV
Osc.: 0.014

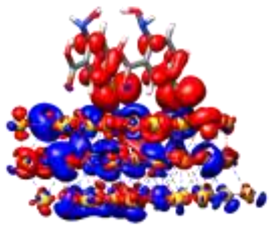

State 98
Energy: 2.345 eV
Osc.: 0.032

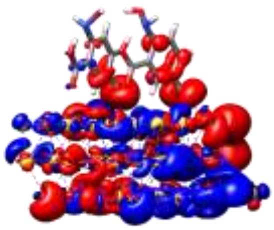

State 105
Energy: 2.422 eV
Osc.: 0.010

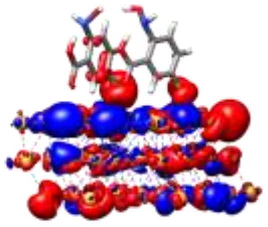

State 109
Energy: 2.479 eV
Osc.: 0.016

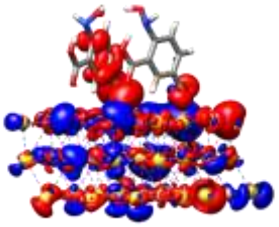

State 112
Energy: 2.501 eV
Osc.: 0.015

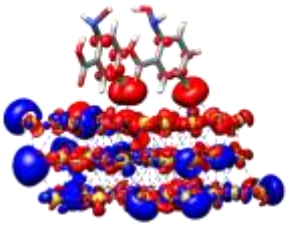

State 114
Energy: 2.529 eV
Osc.: 0.086

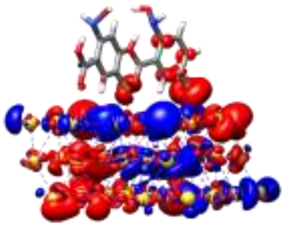

State 115
Energy: 2.545 eV
Osc.: 0.038

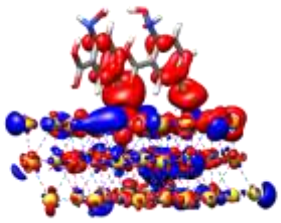

State 119
Energy: 2.580 eV
Osc.: 0.016

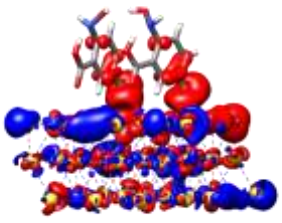

State 121
Energy: 2.592 eV
Osc.: 0.018

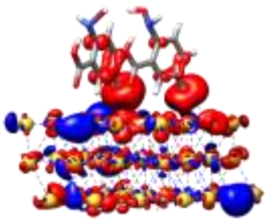

State 122
Energy: 2.604 eV
Osc.: 0.167

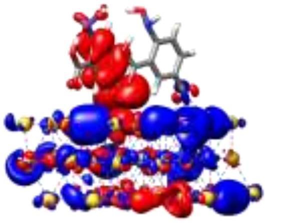

State 124
Energy: 2.624 eV
Osc.: 0.015

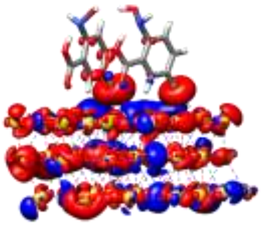

State 126
Energy: 2.638 eV
Osc.: 0.024

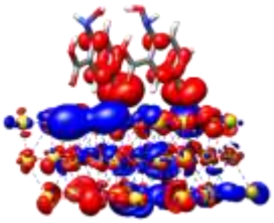

State 128
Energy: 2.660 eV
Osc.: 0.016

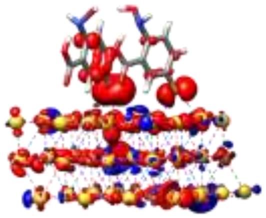

State 130
Energy: 2.682 eV
Osc.: 0.017

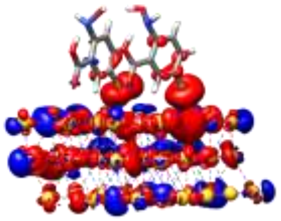

State 134
Energy: 2.720 eV
Osc.: 0.010

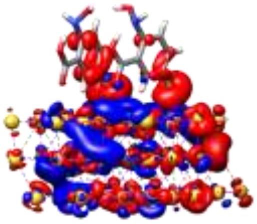

State 135
Energy: 2.725 eV
Osc.: 0.012

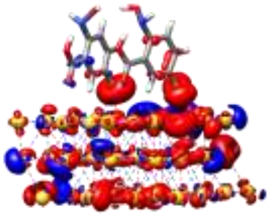

State 136
Energy: 2.738 eV
Osc.: 0.020

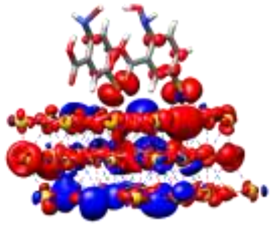

State 137
Energy: 2.742 eV
Osc.: 0.010

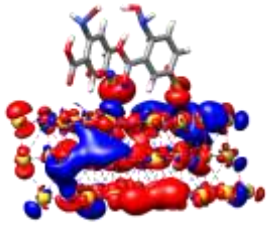

State 138
Energy: 2.749 eV
Osc.: 0.038

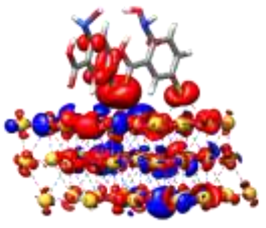

State 139
Energy: 2.764 eV
Osc.: 0.035

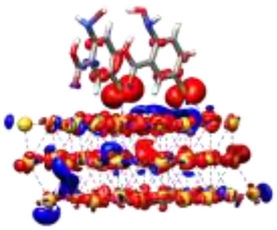

State 140
Energy: 2.765 eV
Osc.: 0.142

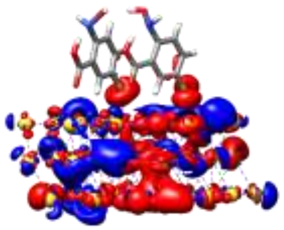

State 141
Energy: 2.775 eV
Osc.: 0.015

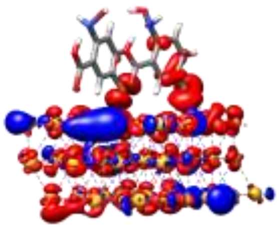

State 142
Energy: 2.793 eV
Osc.: 0.188

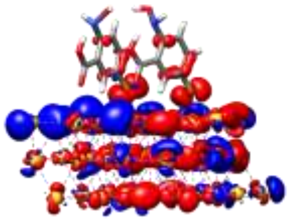

State 143
Energy: 2.798 eV
Osc.: 0.163

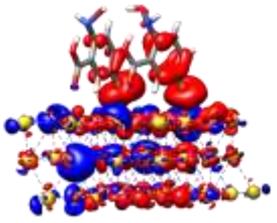

State 144
Energy: 2.806 eV
Osc.: 0.035

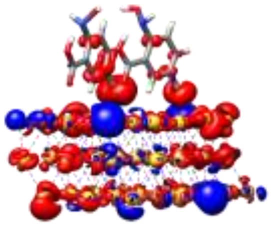

State 145
Energy: 2.817 eV
Osc.: 0.019

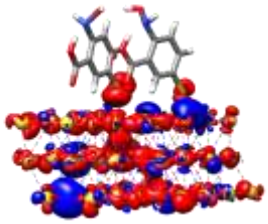

State 146
Energy: 2.821 eV
Osc.: 0.042

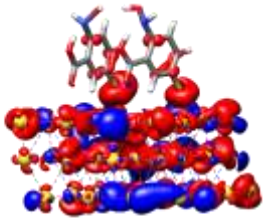

State 147
Energy: 2.829 eV
Osc.: 0.017

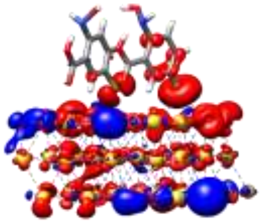

State 149
Energy: 2.838 eV
Osc.: 0.012

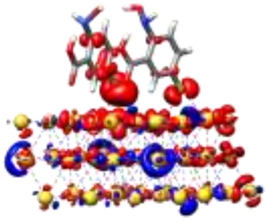

State 150
Energy: 2.853 eV
Osc.: 0.107

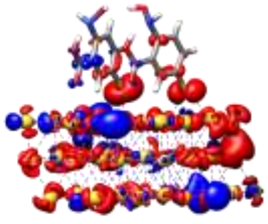

State 151
Energy: 2.860 eV
Osc.: 0.082

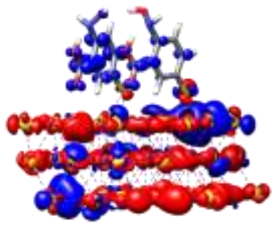<br>State 152<br>Energy: 2.873 eV<br>Osc.: 0.058	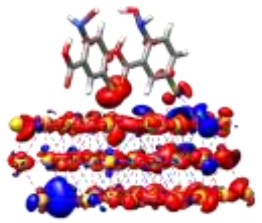<br>State 153<br>Energy: 2.877 eV<br>Osc.: 0.035	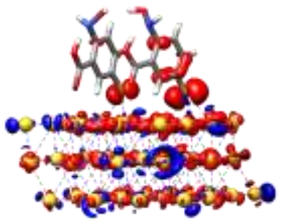<br>State 154<br>Energy: 2.889 eV<br>Osc.: 0.232	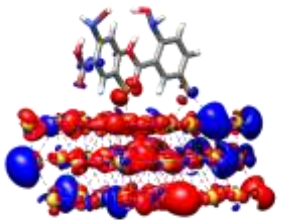<br>State 155<br>Energy: 2.895 eV<br>Osc.: 0.088
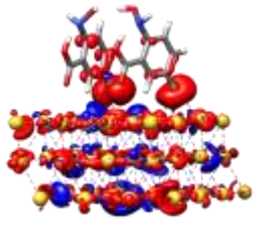<br>State 156<br>Energy: 2.906 eV<br>Osc.: 0.035	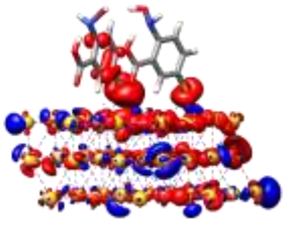<br>State 157<br>Energy: 2.920 eV<br>Osc.: 0.022	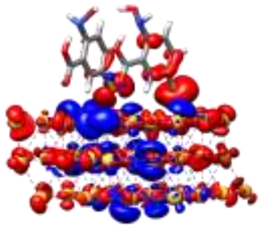<br>State 158<br>Energy: 2.927 eV<br>Osc.: 0.036	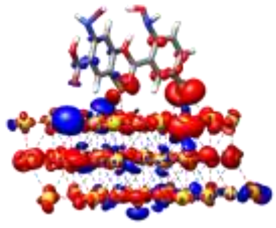<br>State 159<br>Energy: 2.927 eV<br>Osc.: 0.085
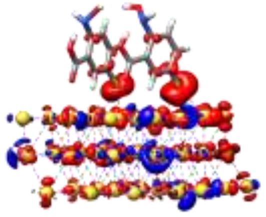<br>State 160<br>Energy: 2.939 eV<br>Osc.: 0.185	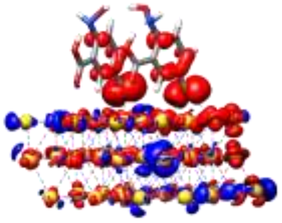<br>State 162<br>Energy: 2.949 eV<br>Osc.: 0.084	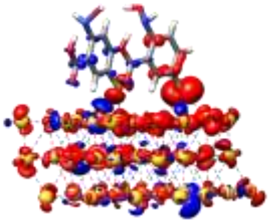<br>State 163<br>Energy: 2.955 eV<br>Osc.: 0.171	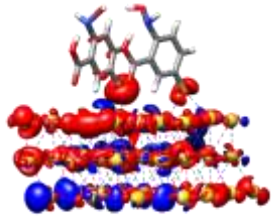<br>State 164<br>Energy: 2.959 eV<br>Osc.: 0.391

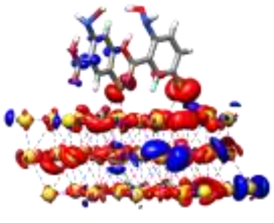<br>State 165<br>Energy: 2.965 eV<br>Osc.: 0.081	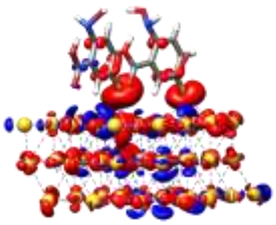<br>State 166<br>Energy: 2.973 eV<br>Osc.: 0.016	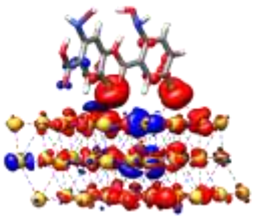<br>State 167<br>Energy: 2.990 eV<br>Osc.: 0.121	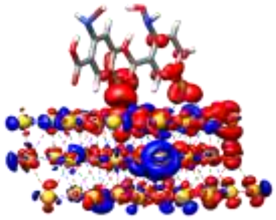<br>State 168<br>Energy: 3.002 eV<br>Osc.: 0.025

References

1. Klimes, J.; Bowler, D. R.; Michaelides, A., Chemical Accuracy for the Van Der Waals Density Functional. *J Phys Condens Matter* **2010**, *22*, 022201.
2. Mortensen, J. J.; Hansen, L. B.; Jacobsen, K. W., Real-Space Grid Implementation of the Projector Augmented Wave Method. *Phys . Rev. B* **2005**, *71*, 035109
3. Enkovaara, J., et al., Electronic Structure Calculations with Gpaw: A Real-Space Implementation of the Projector Augmented-Wave Method. *J Phys Condens Matter* **2010**, *22*, 253202.
4. Lehtola, S.; Steigemann, C.; Oliveira, M. J. T.; Marques, M. A. L., Recent Developments in Libxc — a Comprehensive Library of Functionals for Density Functional Theory. *SoftwareX* **2018**, *7*, 1-5.
5. Frisch, M. J., et al., Gaussian 09, Revision B.01. Gaussian Inc., Wallingford. **2010**.
6. Yanai, T.; Tew, D. P.; Handy, N. C., A New Hybrid Exchange–Correlation Functional Using the Coulomb-Attenuating Method (Cam-B3lyp). *Chem. Phys. Lett.* **2004**, *393*, 51-57.
7. Weigend, F.; Ahlrichs, R., Balanced Basis Sets of Split Valence, Triple Zeta Valence and Quadruple Zeta Valence Quality for H to Rn: Design and Assessment of Accuracy. *Phys Chem Chem Phys* **2005**, *7*, 3297-305.
8. Weigend, F., Accurate Coulomb-Fitting Basis Sets for H to Rn. *Phys. Chem. Chem. Phys.* **2006**, *8*, 1057-1065.
